# Synthesis, Structural Properties and Biological Activities of Novel Hydrazones of 2-, 3-, 4-Iodobenzoic Acid

**DOI:** 10.3390/molecules29163814

**Published:** 2024-08-11

**Authors:** Izabela Czyżewska, Liliana Mazur, Anna Biernasiuk, Anna Hordyjewska, Łukasz Popiołek

**Affiliations:** 1 Chair and Department of Organic Chemistry, Faculty of Pharmacy, Medical University of Lublin, 4A Chodźki Street, 20-093 Lublin, Poland; 50460@student.umlub.pl; 2 Institute of Chemical Sciences, Faculty of Chemistry, Maria Curie–Skłodowska University, Maria Curie–Skłodowska Square 2, 20-031 Lublin, Poland; liliana.mazur@mail.umcs.pl; 3 Chair and Department of Pharmaceutical Microbiology, Faculty of Pharmacy, Medical University of Lublin, 1 Chodźki Street, 20-093 Lublin, Poland; anna.biernasiuk@umlub.pl; 4 Chair and Department of Medicinal Chemistry, Faculty of Medical Sciences, Medical University of Lublin, 4A Chodźki Street, 20-093 Lublin, Poland; anna.hordyjewska@umlub.pl

**Keywords:** iodobenzoic acid derivatives, acylhydrazones, crystal structure, bioactivity, antimicrobial activity, cytotoxicity

## Abstract

Nowadays, searching for novel antimicrobial agents is crucial due to the increasing number of resistant bacterial strains. Moreover, cancer therapy is a major challenge for modern medicine. Currently used cytostatics have a large number of side effects and insufficient therapeutic effects. Due to the above-mentioned facts, we undertook research to synthesize novel compounds from the acylhydrazone group aimed at obtaining potential antimicrobial and anticancer agents. As a starting material, we employed hydrazides of 2-, 3- or 4-iodobenzoic acid, which gave three series of acylhydrazones in the condensation reaction with various aldehydes. The chemical structure of all obtained compounds was confirmed by IR, ^1^H NMR, and ^13^C NMR. The structure of selected compounds was determined by single-crystal X-ray diffraction analysis. Additionally, all samples were characterized using powder X-ray diffraction. The other issue in this research was to examine the possibility of the solvent-free synthesis of compounds using mechanochemical methods. The biological screening results revealed that some of the newly synthesized compounds indicated a beneficial antimicrobial effect even against MRSA—the methicillin-resistant *Staphylococcus aureus* ATCC 43300 strain. In many cases, the antibacterial activity of synthesized acylhydrazones was equal to or better than that of commercially available antibacterial agents that were used as reference substances in this research. Significantly, the tested compounds do not show toxicity to normal cell lines either.

## 1. Introduction

The number of drug-resistant bacterial strains that cause difficult-to-treat infections increases every year. This constitutes a serious problem in modern medicine because currently used antimicrobial agents do not meet expectations [1]. This situation stimulates many groups of researchers around the world to search for novel compounds with higher antimicrobial activity, a wider spectrum of action and lower toxicity [2].

Acylhydrazones constitute a group of compounds which are currently of interest to many scientists due to their varying bioactivity [3,4,5,6]. These compounds possess a –CO–NH–N=CH– moiety. Acylhydrazones can be synthesized by the condensation reaction of the hydrazide of carboxylic acid with aliphatic or (hetero)aromatic ketone or aldehyde [7]. Compounds from this group show various biological properties. They mainly possess antibacterial, antifungal, anti-inflammatory [8], anticancer, cardioprotective, antioxidant, and antiprotozoal activities [5,9,10]. Many examples of bioactive acylhydrazones are described in the scientific literature [1,3,4,5,6,7,8,9,10]. The acylhydrazone moiety is also present in the chemical structure of medicines already available as treatment options, including nitrofurazone, furazolidone and nitrofurantoin [11].

Studies proved that the antibacterial effect of acylhydrazones depends on the lipophilicity and the type of substituent present in the hydrazone part of the compound. The analysis of results published in the scientific literature showed that an increase in the lipophilicity of acylhydrazones is usually associated with an increase in their antibacterial activity [12,13]. Additionally, in our previous studies, the substitution of the iodine atom at the phenyl ring in the acylhydrazone structure was favorable for antimicrobial activity [14,15,16].

The aim of this research was the synthesis, chemical structure confirmation, assessment of antimicrobial activity and cytotoxicity of three series of acylhydrazones of 2-, 3- or 4-iodobenzoic acid. Another objective was to study the molecular and crystal structures of the compounds in terms of changes caused by the introduction of additional substituents (on the phenyl C2 group) or the replacement of halogen substituents I/Br/Cl.

Moreover, considering the literature reports on the mechanochemical synthesis of some groups of hydrazones [17,18,19], including nitrofurantoin and dantrolene [20], we decided to check the possibility of solvent-free preparation of these compounds via vibrational ball mill using the neat grinding (NG) and liquid-assisted grinding (LAG) techniques [21].

## 2. Results

### 2.1. Chemistry

#### 2.1.1. Synthesis and Chemical Characterization

**Scheme 1:**
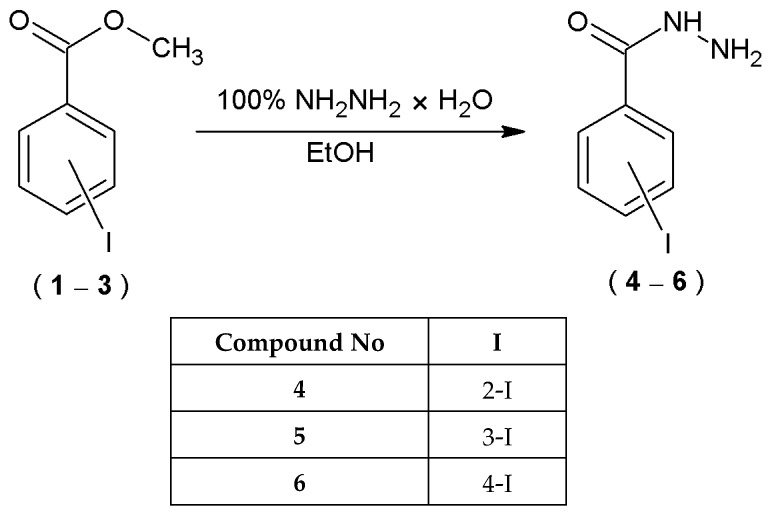
Synthesis of the hydrazides of 2-, 3- or 4-iodobenzoic acid.

**Scheme 2:**
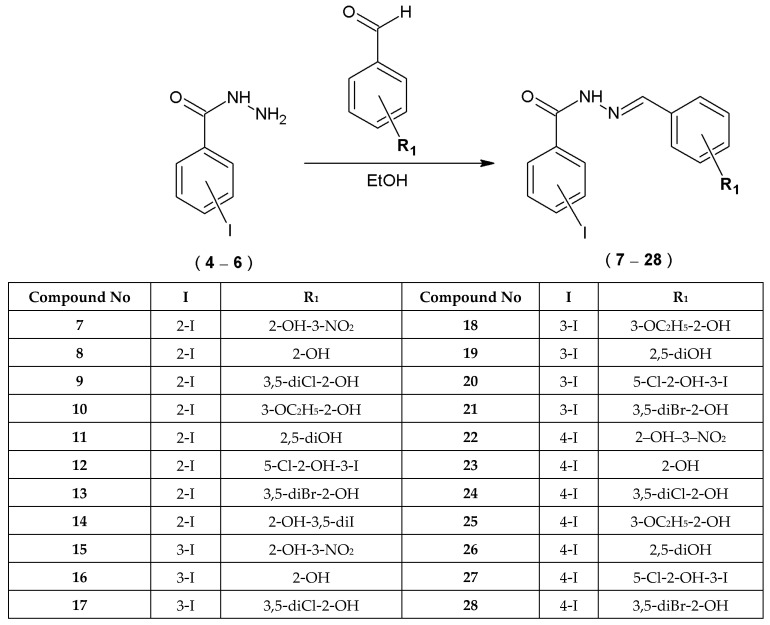
Synthesis of the acylhydrazones of 2-, 3- or 4-iodobenzoic acid.

In this research, a series of twenty-two novel acylhydrazones of 2-, 3- or 4-iodobenzoic acid (**7**–**28**) was synthesized with the use of standard solution. The reactions performed in this research were conducted according to Scheme 1 and Scheme 2. The synthesis was performed in two steps. Firstly, hydrazides of 2-, 3- or 4-iodobenzoic acid (**4**–**6**) were obtained in the reaction of appropriate methyl esters of 2-, 3- or 4-iodobenzoic acid (**1**–**3**) with 100% hydrazine hydrate (Scheme 1). The yields for these reactions were in the range of 67–72%. Then, acylhydrazones (**7**–**28**) were synthesized based on the condensation reaction of an appropriate hydrazide of 2-, 3- or 4-iodobenzoic acid (**4**–**6**) with various substituted aromatic aldehydes (Scheme 2). The yields of the condensation reactions were varied and depended on the aldehyde used. The highest yield (91%) was recorded in the case of the synthesis of *N*-[(3,5-dichloro-2-hydroxyphenyl)methylidene]-3-iodobenzohydrazide (**17**), whereas the lowest yield (51%) was recorded for compound **9**: *N*-[(3,5-dichloro-2-hydroxyphenyl)methylidene]-2-iodobenzohydrazide.

The resulting solids were recrystallized to isolate single crystals suitable for X-ray diffraction studies. For this purpose, organic solvents with different molecular polarities were used. Good-quality single crystals of **9**, **12**, **13**, **14**, **20**, **24**, **26a** were obtained using methanol or acetonitrile as a solvent. Solvate **13**·**ACN** was obtained after the recrystallization of the pure compound from acetonitrile. All remaining compounds were isolated as solid powder. Further attempts at crystallizing the solution while using ethanol and chloroform did not result in any new crystal forms. Single crystals of concomitant polymorphs **26b** and **26c** were isolated from a sample after recrystallization of **26a** from an ethanol with the addition of an equimolar amount of ZnCl_2_. Interestingly, none of the polymorphs **26b** and **26c** were detected after the recrystallization of pure acylhydrazone.

#### 2.1.2. Mechanochemical Preparation of Acylhydrazones

**Figure 1:**
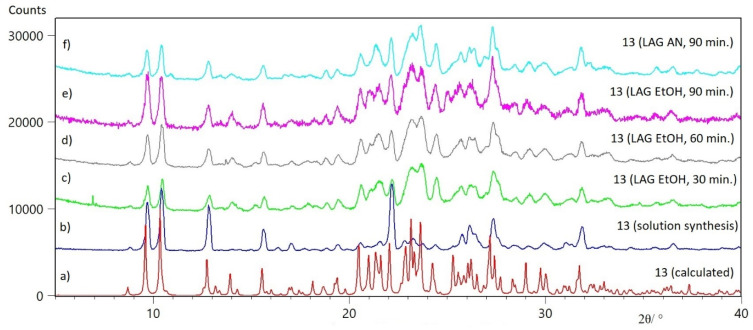
PXRD patterns of compound **13**: (**a**) simulated from the SCXRD data; (**b**) experimental after synthesis from solution; (**c**–**e**) experimental after liquid-assisted grinding (LAG) for 30, 60 and 90 min, respectively, using ethanol as a solvent; (**f**) experimental after LAG for 90 min using acetonitrile.

We attempted to perform a solvent-free synthesis (NG and LAG) of our compounds, taking into account the high efficiency and ease with which the condensation reactions of the reported hydrazides **4**–**6** with aldehydes take place in solution, leading to compounds **7**–**28** (Scheme 2), as well as the results of the mechanochemical synthesis of other acylhydrazones [17,18,19,20,21,22,23,24,25,26] reported in the literature. For this purpose, four hydrazones, i.e., **13**, **14**, **24**, and **26**, were selected as model systems. In each experiment, the equimolar amounts of the appropriate hydrazide and aldehyde, in the presence of a few drops (η = 0.25 µL·mg^−1^) of ethanol or acetonitrile (LAG) or without the solvent (NG), were ground for 90 min in stainless steel jars, using a vibrating ball mill. In all experiments, the resulting samples were obtained as solid powders, however, with a higher degree of crystallinity after LAG, in particular when acetonitrile was used as a solvent. NG led to microcrystalline (**26**) or amorphous solids. Our research confirmed quantitative conversion of the reactants in all LAG experiments on the basis of the comparison of powder X-ray diffraction (PXRD) patterns recorded for the substrates and the products, as well as the theoretical patterns of the products, calculated from the single-crystal X-ray diffraction (SCXRD) data (Figure 1 and Figure S1 in Supplementary Materials). Further optimization studies on a small reaction scale (80 mg), performed for acylhydrazone **13** revealed the complete conversion of the substrates even after 30 min of milling (LAG, ethanol). Moreover, for compounds **13**, **14** and **24** the same crystal forms were obtained after the LAG and solution synthesis (Figure 1 and Figure S1 in Supplementary Materials). In the case of polymorphic compound **26**, the PXRD data revealed the formation of the densest form **26c** (Table 1) in the LAG experiments. Interestingly, polymorph **26c** was available from the solution only after recrystallization from the mixture with ZnCl_2_; the solution synthesis and further recrystallization experiments of pure hydrazone led solely to the polymorph **26a** (with the smallest density), independent of the applied solvent.

#### 2.1.3. X-ray Diffraction Studies

**Figure 2:**
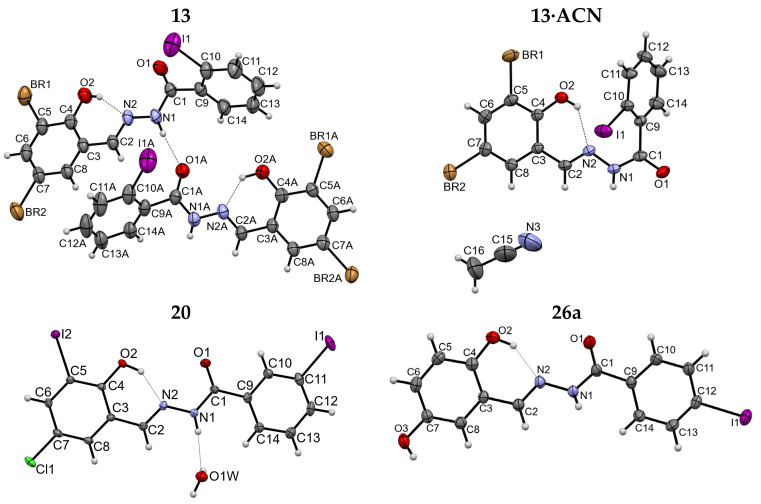
Perspective view of the molecules constituting the asymmetric part in crystals **13**, **13**∙**ACN**, **20** and **26a** with the atom-numbering scheme. Thermal ellipsoids are drawn at the 50% probability level. Dashed lines indicate the hydrogen bonds.

**Molecular structure.** The relevant geometric parameters for the studied crystals are given in Tables S2–S6 in Supplementary Materials. Molecular plots with the atom-labelling schemes are presented in Figure 2 and Figure S2 in Supplementary Materials. In general, the compounds crystallize in common centrosymmetric space groups (*P*2_1_/*c*, *C*2/*c*, *Pbca*), mostly with two (**9**, **12**, **13**, **14** and **24**) acylhydrazone molecules in the asymmetric unit of the crystal (Table 1). The only exception is the 2,5-dihydroxyphenyl derivative **26**, for which three polymorphs were detected; all of them crystallize in chiral space groups with one molecule in the asymmetric part of the unit cell. The frequently appearing lowest density polymorph **26a** crystallizes in the orthorhombic *Pca*2_1_ space group, giving needle-like crystals, whereas the denser monoclinic (*P*2_1_) polymorphs **26b** and **26c** form plate-shaped crystals. There are extensive similarities in the unit cell parameters, molecular conformations, and intermolecular interactions in crystals **9**, **13**, **14**, which indicates some extent of their isostructurality.

Unlike many other reported acylhydrazones [27,28,29], the compounds do not tend to incorporate water molecules into the crystals; the only exception is derivative **20**, which was obtained solely as a monohydrate. Compound **13** can exist in both its unsolvated crystal form and as a solvate **13**∙**ACN**, the latter being obtained by recrystallization of the crude product from acetonitrile at room temperature. It is worth noting that both crystal forms (**13** and **13**∙**ACN**) can grow simultaneously using the latter solvent.

**Figure 3:**
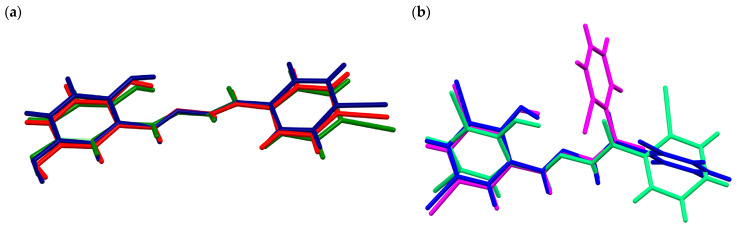
Molecular overlay of the conformers found in: (**a**) polymorphic modifications **26a** (red line), **26b** (green line) and **26c** (dark blue line); (**b**) unsolvated crystal **13** (molecule 13A—blue line, molecule 13B—green line) and its solvate **13**∙**ACN** (pink line).

**Figure 4:**
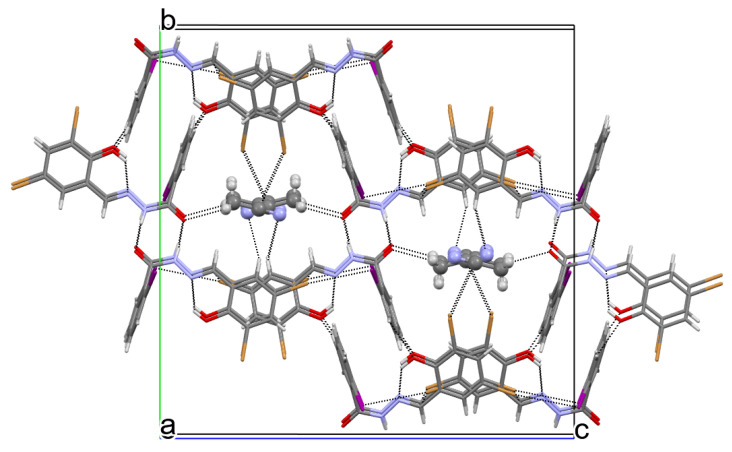
Part of the crystal structure of **13**·**ACN** in view along the *a* axis, showing the formation of channels filled in by the solvent molecules.

As follows from the X-ray data, in all studied crystals the acylhydrazone molecules appear in their keto-imino tautomeric form with the *trans* configuration around the C2=N2 double bond of the hydrazone unit (Figure 2), excluding crystal **13**∙**ACN** with the *cis* arrangement of the hydrazide C1(=O1)–N1–N2 function. The central C3–C2–N2–N1–C1–C8 spacer unit is effectively planar with the all-*trans* extended-chain conformation (apart from **13**∙**ACN**), as evidenced by the appropriate torsion angles being close to 180°. Small rotations in this unit are observed mainly around the N1–N2 bond (Table S4 in Supplementary Materials). This is in agreement with our previous findings concerning some other N1-acylhydrazones [28,29]. It is worth mentioning that the theoretical calculations performed for quite similar acylhydrazones [28] indicated the bent conformation as energetically privileged. The more frequent occurrence of the extended conformation results probably from more favorable energetics of intermolecular interactions and the total energy of the crystal lattice. Another common conformational feature in the molecules analyzed in this paper is the *cis* arrangement of hydroxyl O2 with respect to the imine N2 atom, as defined by the O2–C4–C3–C2 and C4–C3–C2–N3 torsion angles, which deviate by no more than ±7° from 0°. Such conformation enables the creation of a very short, directional O2–H2o···N2 hydrogen bond, which additionally stabilizes coplanarity of the phenyl ring and the hydrazone unit. Moreover, there are no significant differences in the bond lengths and angles (Tables S2 and S3 in Supplementary Materials) within the central spacer. In general, single and double bonds are easily distinguishable. What is more, the interatomic distances are within typical ranges for N1-aroylhydrazones [28,29]. The most visible conformational dissimilarities between the symmetry independent molecules in crystals **9**, **12**, **13**, **14** concern orientation of C9<<C14 phenyl ring with respect to the remaining fragments, mostly due to the rotation around the C1–C9 bond (Figure 3 and Figures S3–S5 in Supplementary Materials). This is evidenced by the torsion angle N1–C1–C9–C10 being in the range ±140.7(6)°–146.5(4)° in one molecule and ±116.6(6)°–122.0(9)° in the other one (Table S4, Figure S2 in Supplementary Materials). Except for the molecule **13**∙**ACN**, which adopts a bent conformation, all others show an extended conformation. However, apart from molecules **20** and **26a** other molecules are not flat, as evidenced by the dihedral angle values between the best planes of the phenyl rings, being mostly in the range 50–70° (Table S5 in Supplementary Materials). The observed changes point to a high conformational lability of the studied acylhydrazones as well as to strong influence of the crystal field, mainly intermolecular interactions, on the molecular conformation adopted in the solid state. This is also confirmed by the bent conformation of compound **13**, adopted in **13**∙**ACN** solvate (Figure 3b). As a result, the ‘host’ molecules are arranged to form channels in the crystal, with their hydrophobic units directed into the channels (Figure 4 and Figure S4 in Supplementary Materials). This assembling model enables effective binding of solvent molecules inside the channels and makes the solvate quite stable at room temperature.

The molecules in the non-centrosymmetric crystal structures of the polymorphs **26a**–**26c** are characterized by a similar geometry (Figure 3a). Small deviations are mostly due to rotation around the N1–N2 and C1–C9 bonds; however, the differences do not exceed 15°, which suggests the conformational adjustment, rather than conformational polymorphism [30] in this case.

**Figure 5:**
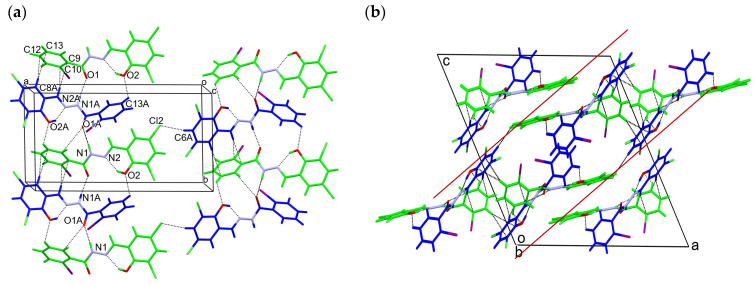
Part of the crystal structure of **9** showing (**a**) supramolecular chains stabilized via strong N1–H1n∙∙∙O1A/N1A–H1nA∙∙∙O1 (x, y + 1, *z*) hydrogen bonds and weak C–H∙∙∙O/π interactions; (**b**) crystal packing viewed along the *b* axis with marked 2D layer parallel to the (−102) crystallographic plane. Molecules **9-A** and **9-B** are marked in green and blue, respectively. Dashed lines indicate hydrogen bonds.

**Crystal structure.** Among the intermolecular interactions involved in the stabilization of solvent-free crystals of the reported acylhydrazones, hydrogen bonds of the N–H···O type involving the amide groups seem to play the dominant role (Table S6 in Supplementary Materials). In the series of isostructural crystals **9**, **13**, **14**, the adjacent A and B molecules are connected by relatively short N1–H1n···O1A and N1A–H1nA···O1 hydrogen bonds (symmetry operators in Table S6 in Supplementary Materials), which together with numerous weak C–H···O and C–H···π contacts, mostly between the phenyl rings and hydroxyl or carbonyl O-atoms, stabilize single molecular chains, propagated along the *b*-axis (Figure 5a). The chains are arranged around the 2_1_ axis, giving more complex 1D motif. The adjacent inversion-related helical motifs create supramolecular layers parallel to the (−102) crystallographic plane (Figure 5b). Among the intermolecular interactions, involved in stabilization of the layers, weak C_ar_–H···Hal and C_ar_–H···π_ar_ hydrogen bonds seem to be dominant. Interlayer stabilization is accomplished mainly through the C_ar_–H···O/N/Hal contacts.

**Figure 6:**
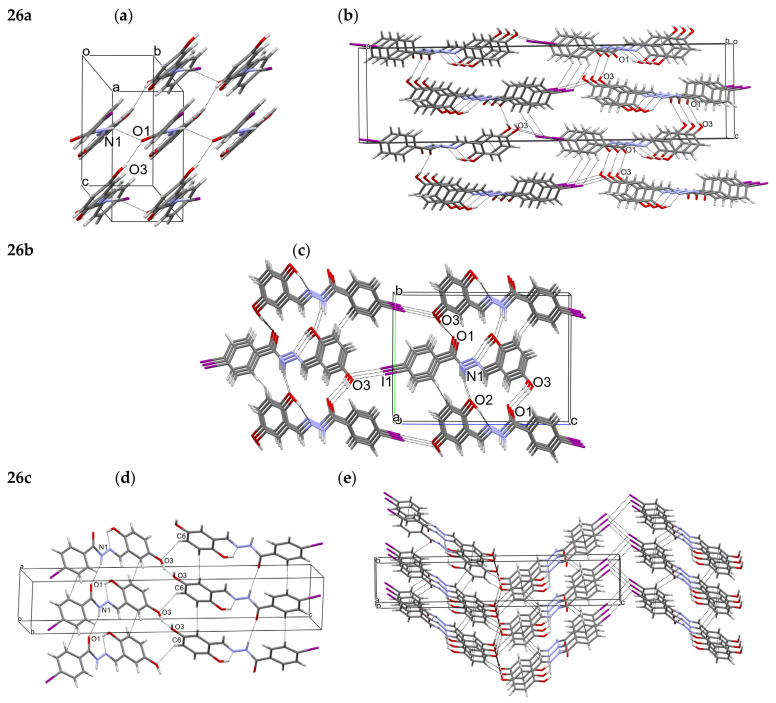
(**a**) Part of the crystal structure of **26a** showing hydrogen-bonding motifs; (**b**) crystal packing in **26a** viewed along the *b* axis; (**c**) crystal packing in **26b** viewed down the *a* axis; (**d**) hydrogen-bonding patterns in crystal **26c**, (**e**) part of the crystal structure of **26c** in view along the *a* axis. Dashed lines indicate inter- and intramolecular interactions.

In polymorphs **26a**, **26b** and **26c**, the molecules are arranged in a more diverse mode. For the densest form **26c** the main forces which promote the self-assembly of molecules seem to result from the N1–H1n∙∙∙O1(*x* − 1, *y*, *z*) and O3–H3o∙∙∙O3(*x* − 1, *y* + 1, *z*) hydrogen bonds (Table S6 in Supplementary Materials). As a result, double 2D supramolecular layers are formed (Figure 6d,e) parallel to (001) plane. Interlayer stabilization is provided by the halogen-bonding interactions between I atoms from the adjacent 2_1_-axis-related molecules, with the I1∙∙∙I1 distance of 3.903(8) Å and the C12–I1∙∙∙I1 angle of 106.0(5)°. The supramolecular (001) layers also accrue in crystal **26a**, but their structure is different. In this case, they result from the combination of overlapping (012) molecular ribbons, stabilized by O3–H3o∙∙∙O1(-*x* + 1/2, *y* + 1, *z* − 1/2) and interconnected through the N1–H1n∙∙∙O1(*x*, *y* + 1, *z*) hydrogen bonds (Table S5 in Supplementary Materials; Figure 6a,b). C–H∙∙∙I contacts dominate between the layers. Interestingly, in the last polymorph, **26b** there are no amide-amide hydrogen bonds. In this case, the main supramolecular motifs are based on strong O–H∙∙∙O hydrogen bonds, involving hydroxyl O2–H, O3–H groups and just amide N1-H and O1 atoms (Figure 6c). The resulting (001) layers are held together by I∙∙∙O contacts with the interatomic distances of 3.044(7) Å.

The incorporation of water molecules into the crystal of **20** results in the formation of a quite different supramolecular framework and a hydrogen-bonding pattern. In general, water molecules fill in the gaps and serve as a supramolecular linkage between the ‘host’ units arranged around the 2_1_ axis, thus creating molecular chains along the *b* axis. The chains are the components of the complex supramolecular layers (Figure S5 in Supplementary Materials).

### 2.2. Microbiology

The results of our study are presented in Table 2, Table 3 and Table 4 showing that some of the newly synthesized compounds **4**–**28** reveal antimicrobial effects against the reference bacteria and yeasts.

As follows from Table 2, it is indicated that synthesized compounds **7**–**14** showed antimicrobial effects against the reference Gram-positive bacteria and yeasts. However, no activity was found against the Gram-negative rods from *Enterobacterales* and non-fermenting bacteria (*Ps. aeruginosa* ATCC 9027). The widest spectrum of activity was indicated by compounds **12** and **14**. The microorganisms belonging to *Micrococcus luteus* ATCC 10240 were the most sensitive to both substances at the minimal inhibitory concentrations (MIC) of 31.25 µg/mL and 62.5 µg/mL, respectively. Moreover, compound **14** exhibited a good or moderate effect towards all Gram-positive bacteria with MIC = 62.5–500 µg/mL and MBC = 125–1000 µg/mL. Additionally, this compound showed a favorable bactericidal effect with MBC/MIC = 1–4. In the case of substance **12**, the MIC values ranged from 31.25 to 1000 µg/mL (MBC = 250–>1000 µg/mL). The remaining compounds **4** and **7** were slightly weaker. Some staphylococci and bacilli were insensitive to them. In turn, compounds **10** and **11** had no antimicrobial effect against the studied bacteria from ATCC.

Moreover, compounds **12** and **14** showed a moderate or mild effect and lower activity against the reference strains of yeasts from *Candida* spp. (MIC = 250–1000 µg/mL and MFC ≥ 1000 µg/mL). Unfortunately, the studied compounds **4** (hydrazide), **7**, **10**, and **11** (acylhydrazones) showed a mild activity or were inactive towards fungi.

The values of MBC/MIC or MFC/MIC were from 1 to 8. However, for most compounds, MBCs or MFCs were >1000 µg/mL and it was not possible to determine either their index MBC/MIC or MFC/MIC, nor a bactericidal/bacteriostatic or fungicidal/fungistatic effect.

As shown in Table 3, the highest activity among compounds **15**–**21** was exhibited by compounds **20** (very strong), and **21** (strong or very strong) with MIC = 1.95–7.81 µg/mL and MIC = 3.91–15.62 µg/mL, respectively. The MBC values ranged from 3.91 to 62.5 µg/mL. That activity was very beneficial with the bactericidal effect (MBC/MIC = 1–4) against the Gram-positive bacteria. A similar antibacterial effect towards these microorganisms was revealed for compound **17** with MIC ranging from 7.81 to 31.25 µg/mL. Substance **15** was slightly less active (MIC = 31.25–500 µg/mL) compared with compounds **17**, **20**, and **21**. The bacteria belonging to: *Staphylococcus epidermidis* ATCC 12228, *Micrococcus luteus* ATCC 10240, and *Bacillus subtilis* ATCC 6633 were only sensitive to compounds **5** and **18** at the minimal inhibitory concentrations from 31.25 to 1000 µg/mL. In the case of substances **16** and **19**, no activity was demonstrated.

It is worth adding that acylhydrazone **20**, as the only one among the investigated substances, had the widest spectrum of activity towards all reference Gram-positive or Gram-negative bacteria and fungi. For this substance (with MIC = 500–1000 µg/mL), a mild or moderate effect was demonstrated towards the reference Gram-negative rods from *Enterobacterales* (*Bordetella bronchiseptica* ATCC 4617, *Escherichia coli* ATCC 25922, *Klebsiella pneumoniae* ATCC 13883, *Proteus mirabilis* ATCC 12453, *Salmonella typhimurium* ATCC 14028) and the non-fermenting bacteria, such as *Pseudomonas aeruginosa* ATCC 9027. The Gram-negative bacteria were susceptible only to substance **20**. Unfortunately, the remaining synthesized compounds were inactive against these microorganisms.

Moreover, compounds **19**, **20**, and **21** indicated an inhibitory effect on the growth of all reference strains of yeasts from *Candida* spp. at MIC = 125–500 µg/mL with good and moderate antifungal activity. Among fungi, *C. parapsilosis* ATCC 22019 strains were the most sensitive to compounds **19** and **20** and *C. glabrata* ATCC 90030 to compounds **20** and **21** with MIC = 125 µg/mL and MFC > 1000 µg/mL, respectively. These substances showed mostly a fungistatic effect (MFC/MIC > 4).

Among newly synthesized compounds **22**–**28**, a lower antimicrobial activity (Table 4) compared with substances **15**–**21** (Table 3) was found. Compound **27** was the most active and had the widest spectrum of action towards all studied microorganisms. The inhibitory effect of this substance on the growth of Gram-positive bacteria was shown at MIC ranging from 31.25 to 125 µg/mL. The MBC values were the same or from two- to four-times higher (31.25–500 µg/mL). These data confirm the bactericidal effect of compound **27** towards these bacteria (MBC/MIC = 1–4) and its good activity. Another substance among these acylhydrazones, i.e., compound **23**, indicated a moderate or mild antimicrobial effect towards Gram-positive bacteria with MIC = 500–1000 µg/mL and MBC > 1000 µg/mL. The remaining compounds, e.g., **6** (hydrazide), **24**, **25**, and **26** (acylhydrazones) showed a similar activity to compound **16** or no activity. No antimicrobial effect against these bacteria was demonstrated for substances **22** and **28**.

Only one substance **27**, showed activity against Gram-negative bacteria. This activity was moderate with MIC = 500 µg/mL for all rods from *Enterobacterales*, and mild with MIC = 1000 µg/mL for *Ps. aeruginosa* ATCC 9027 (MBC = 500–≥1000 µg/mL). The other compounds were not active towards these bacteria.

The studies of antifungal activity proved that acylhydrazone **27** exhibited the most satisfactory effect. Its activity was good towards all reference *Candida* spp. (MIC = 31.25–62.5 µg/mL, and MFC = 62.5 µg/mL), except *C. albicans* ATCC 10231. In this case, the effect was strong (MIC = 15.62 µg/mL and MFC = 31.25 µg/mL). Additionally, compound **27**, showed a fungicidal activity against these yeasts (MFC/MIC = 1–2). Substances **26** and **28** also exhibited an antifungal action towards these fungi, but their activity was lower (MIC = 250–1000 µg/mL and MFC > 1000 µg/mL). Some compounds, e.g., **6** (hydrazide), **23**, **24**, and **25** (acylhydrazones) showed a similar activity to compounds **26** and **28** (acylhydrazones) or were inactive. Acylhydrazone **22** had no antifungal activity.

### 2.3. Cytotoxicity Study

The 24 h culture of the L929 line showed that the tested compounds did not affect the proliferation potential to a great extent. The cytotoxicity of tested compounds **14**, **20**, **21** and **27** remained at the level of approximately 20%. Only compound **14** at a dose of 75 µM caused cytotoxicity at around 40%. The same compound, during a 48 h culture, at a dose of 25 μM, behaved in an antagonistic way—it increased viability by over 100%. The cytotoxicity of the remaining compounds during the 48 h culture was comparable to the 24 h culture (Tables S7 and S8, Figures S6–S13 in Supplementary Materials).

The 24 h culture of the A549 line showed that the tested acylhydrazones **14**, **20**, **21** and **27** did not cause high cytotoxic effects on the cells of this line either. Compound **27** at a dose of 100 µM caused even an increase in the proliferation of cancer cells. During 48 h culture with the tested compounds, cell viability remained at the level of 85% (Tables S9 and S10, Figures S14–S21 in Supplementary Materials).

The 24 h culture of the HeLa line showed that the most active compound that caused the highest cytotoxicity towards this cell line was **27**, which at a dose of 50 µM and at a dose of 100 µM caused 37% and 41% cell cytotoxicity, respectively. The remaining compounds caused cytotoxicity ranging from 15% to 25%. During a 48 h culture, the compound **27** was also the most effective, causing cytotoxicity of approximately 50% at doses of 50 µM and 75 µM (Tables S11 and S12, Figures S22–S29 in Supplementary Materials).

The 24 h and 48 h cultures of the T47D cell line showed that the cytotoxicity of all tested compounds remained at a similar level and amounted to approximately 20–30%. Compound **27** had the greatest cytotoxic potential against the cervical cancer cells for 48 h culture during the initial screening (Tables S13 and S14, Figures S30–S37 in Supplementary Materials).

The IC_50_ dose could not be determined in this study because, no compound (apart from compound **27** against the HeLa cell line) caused a 50% decrease in viability of any cell line.

The lack of a highly cytotoxic effect of the tested compounds may be due to their high molecular weight, and therefore poor penetration into the cell and poor action at the level of the cytoplasm and nucleus. Moreover, the concentrations used in the experiment (10–100 µM) may have turned out to be too poor to cause a cytotoxic effect. The only exception is compound **27** in relation to the HeLa cell line, which caused cytotoxicity of up to 50% (48 h culture—at doses of 50 µM and 75 µM).

## 3. Discussion

### 3.1. Chemistry

The synthesis of hydrazides and acylhydrazones is shown in Scheme 1 and Scheme 2. Hydrazides of 2-, 3-, or 4-iodobenzoic acid (**4**–**6**) were obtained by heating commercially available methyl esters of 2-, 3-, or 4-iodobenzoic acid (**1**–**3**) dissolved in ethanol with 100% hydrazine hydrate (Scheme 1). The obtained hydrazides were subjected to condensation reactions with appropriate aldehydes and the described acylhydrazones (**7**–**28**) were obtained (Scheme 2).

The chemical structure of synthesized compounds (**4**–**28**) was fully impenitent by means of IR, ^1^H NMR, ^13^C NMR data, X-ray crystallography and powder diffraction studies. Suitable spectral and physico-chemical properties are included in the Experimental section.

In the IR spectra of hydrazides of 2-, 3-, or 4-iodobenzoic acid (**4**–**6**), there were peaks for the NH and NH_2_ groups at 3177–3203 and 3294–3312 cm^−1^, respectively, and for the C=N bond at 1618–1624 cm^−1^. Similarly, singlet peaks for the hydrogen atom of NH group at δ 9.52–9.86 ppm and for the proton of the NH_2_ group in the range of δ 4.51–4.54 ppm were found in ^1^H NMR spectra. The presence of the above-mentioned signals in both IR and ^1^H NMR spectra confirms the correctness of the substitution reaction and the replacement of the OCH_3_ group present in the esters (**1**–**3**) by the hydrazine fragment NHNH_2_ present in the hydrazides of 2-, 3-, or 4-iodobenzoic acid (**4**–**6**).

Acylhydrazones of 2-, 3-, or 4-iodobenzoic acid (**7**–**28**) gave also typical signals for this group of compounds in the IR, ^1^H NMR and ^13^C NMR spectra. In the IR spectra, we observed peaks corresponding to the NH group and the C=N bond at 3099–3324 cm^−1^ and 1574–1652 cm^−1^, respectively. In the ^1^H NMR, a typical singlet signal for the proton of the =CH group within δ 8.26–8.72 ppm and for the hydrogen atom of the NH group in the range of 10.30–12.87 ppm was observed. Similarly, in ^13^C NMR spectra, the signal for the carbon atom of the =CH group appeared in the range of δ 144.52–148.92 ppm, whereas the carbon atom of the carbonyl group (C=O) was found at δ 164.02–165.49 ppm. Other aliphatic and aromatic fragments of synthesized acylhydrazones were found in the registered spectra (IR, ^1^H NMR and ^13^C NMR) at predicted values. The examples of IR, ^1^H NMR and ^13^C NMR spectra of synthesized hydrazides (**4**, **5**, **6**) and acylhydrazones (**20**, **24**, **26**) are presented in Supplementary Materials (Figures S38–S55).

### 3.2. Microbiology

Considering the in vitro screening results (Table 2, Table 3 and Table 4) obtained in this research it can be concluded that some of the synthesized novel acylhydrazones of 2-, 3- or 4-iodobenzoic acid had a significant activity which, in many cases, was higher or equal to the activity of commonly used antimicrobial agents.

Acylhydrazone **20** showed a thirty times higher activity (MIC = 1.95 µg/mL) towards *Micrococcus luteus* ATCC 10240, compound **21** possessed an activity which was fifteen times higher (MIC = 3.91 µg/mL), compound **17** was four times more active (MIC = 15.62 µg/mL), whereas compounds **12**, **15**, **18**, and **25** displayed a twice higher activity (MIC = 31.25 µg/mL), and **14** showed a similar activity (MIC = 62.5 µg/mL) to nitrofurantoin (MIC = 62.5 µg/mL). The activity against *Micrococcus luteus* ATCC 10240 was not dependent on the substitution of the iodine atom in iodobenzoic acid. Some activity was shown for acylhydrazones of 2-, 3- as well as 4-iodobenzoic acid derivatives. The substitution at the phenyl ring from the hydrazone part was crucial for the activity against *Micrococcus luteus* ATCC 10240. The most significant activity was displayed when phenyl ring was substituted with: 5-chloro-2-hydroxy-3-iodo (**12**, **20**), 2-hydroxy-3,5-diiodo (**14**), 2-hydroxy-3–nitro (**15**), 3,5-dichloro-2-hydroxy (**17**), 3-ethoxy-2-hydroxy (**18**, **25**) and 3,5-dibromo-2-hydroxy (**21**) groups.

Considering the antibacterial activity against *Bacillus subtilis* ATCC 6633 acylhydrazones **17**, **20** and **21** were detected to have a twice higher activity (MIC = 7.81 µg/mL) than cefuroxime (MIC = 15.62 µg/mL) and compound **18** showed the activity equal to that of ampicillin against this bacterium (MIC = 62.5 µg/mL). Compound **20** showed four times higher activity (MIC = 7.81 µg/mL) towards the other bacterium from this genus *Bacillus cereus* ATCC 10876 and compounds **17** and **21** displayed the activity twice higher (MIC = 15.62 µg/mL) than cefuroxime (MIC = 31.25 µg/mL). As far as the *Bacillus* species strains are concerned, the highest activity was displayed by acylhydrazones of 3-iodobenzoic acid substituted with 3,5-dichloro-2-hydroxy (**17**), 3-ethoxy-2-hydroxy (**18**), 5-chloro-2-hydroxy-3-iodo (**20**) and 3,5-dibromo-2-hydroxy (**21**) substituents at the phenyl ring.

The activity of the tested acylhydrazones towards the bacteria from *Staphylococcus* spp is particularly worth emphasizing. Compound **20** showed an eight times higher activity (MIC = 3.91 µg/mL) than nitrofurantoin (MIC = 7.81 µg/mL) against the MRSA strain of *Staphylococcus* (*S. aureus* ATCC 43300) and against *Staphylococcus epidermidis* ATCC 12228 it had an activity twice higher (MIC = 1.95 µg/mL) than nitrofurantoin (MIC = 3.91 µg/mL). Additionally, acylhydrazone **21** had a twice higher activity (MIC = 7.81 µg/mL) against *Staphylococcus aureus* ATCC 25923 than nitrofurantoin (MIC = 15.62 µg/mL). In terms of the antibacterial activity against *Staphylococcus* spp., the most favorable was the substitution with the iodine atom at position 3 at the phenyl ring of benzoic acid and with 5-chloro-2-hydroxy-3-iodo (**20**) and 3,5-dibromo-2-hydroxy (**21**) substituents at the phenyl ring from the hydrazone part.

The antibacterial activity of synthesized acylhydrazones of 2-, 3- or 4-iodobenzoic acid (**7**–**28**) was much higher in comparison with similar acylhydrazones but obtained from non-iodine substituted benzoic acid, as reported by Siddique et al. and Manikandan et al. [31,32]. An analysis of the values of the zone of inhibition growth of the compounds synthesized by Manikandan et al. revealed that among obtained acylhydrazones, only *N*-(4-fluorobenzylidene)benzohydrazide showed satisfactory antibacterial activity mainly against Gram-positive bacterial strains (the zone of bacterial growth inhibition: ZOI = 6–7 mm) [32], whereas in the research performed by Siddique et al., only acylhydrazone with 4-chlorophenyl substituent displayed activity towards Gram-positive bacteria, especially *Bacillus subtilis* strain (ZOI = 8 mm) [31].

Backes et al. reported synthesis and antifungal activity screening of acylhydrazones of benzoic acid [33]. The most significant activity in that report was displayed acylhydrazones substituted with: 2-hydroxyphenyl, 2-hydroxy-5-methylphenyl, 2-hydroxy-5-methoxyphenyl, and 5-chloro-2-hydroxyphenyl from hydrazone part against *Candida* spp.: *C. albicans* and *C. glabrata* (MIC_80_ = 0.5–4 µg/mL) [33]. In general comparison with acylhydrazones described in current research, the activity was lower but we used different substituents. Compound **27** (MIC = 31.25–62.5 µg/mL, and MFC = 62.5 µg/mL) showed the highest antifungal activity towards all reference *Candida* spp. among acylhydrazones of 2-, 3- or 4-iodobenzoic acid (**7**–**28**). In the case of the activity towards *C. albicans* ATCC 10231, the fungicidal effect was even better (MIC = 15.62 µg/mL and MFC = 31.25 µg/mL, MFC/MIC = 1–2).

It is also worth to mention that in comparison with starting compounds (hydrazides of 2-, 3- or 4-iodobenzoic acid—compounds numbered **4**, **5**, **6**) acylhydrazones (**7**–**28**) displayed much higher bioactivity in terms both antibacterial and antifungal properties, which proves that hydrazone the moiety and substituents from hydrazone part are essential to show antimicrobial activity (Table 2, Table 3 and Table 4).

Additionally, according to the literature cited in the Introduction section [12,13], the structures containing hydrophilic substituents, e.g., OH, do not show a good activity while the compounds with the hydrophobic substituents such as Cl, I, Br reveal a medium or high antibacterial activity (**12**, **14**, **17**, **20**, **21**, **27**).

Based on the scientific literature findings and the results of our current research presented above, acylhydrazones display a significant activity, especially against the Gram-positive bacteria, but worse against the Gram-negative bacteria and fungi.

### 3.3. Cytotoxicity Study

As follows from our study the cytotoxicity results of the tested compounds showed that the newly synthesized acylhydrazones did not cause statistically significant changes in cell proliferation in the range of the tested doses. The problem may be their high molar mass. One of the Lipiński’s rules that must be met for a compound to qualify as a drug is that the molar mass cannot exceed 500 Da. In our study, half of the compounds had a molecular mass higher than 500 Da. (examples **14**, **20**, **21** and **27**). Thus, the lack of a high cytotoxic effect of the tested compounds may be due to their high molecular weight, resulting in poor penetration into the cell and poor action at the level of the cytoplasm and nucleus. Moreover, the concentrations used in the experiment (10–100 µM) may have turned out to be too weak to cause a cytotoxic effect. The only exception was compound **27** in relation to the HeLa line, which caused cytotoxicity of up to 50% (48 h culture—doses of 50 µM and 75 µM). Our results were similar to those obtained by other researchers like Puskullu et al. [34]. Their compounds also showed weaker cytotoxic activity on the A549 and MCF–7 cell line (reduced cell viability to 70% and 75%, respectively) [34]. On the other hand, Al Rasheed et al. [35] stated in their paper that some of the compounds they tested had an IC_50_ value of 1.0 µM against breast cancer lines [35].

In turn, the lack of a negative effect on fibroblasts (L929 line) means that these compounds can be used in further antibacterial or antiviral tests. This does not eliminate their antiproliferative potential against other types of cancer. In this study, only the three most common types of cancer in Poland were selected (cervical cancer, breast cancer and lung cancer). For example, the research carried out by Castrillón-López et al. [36] on hydrazones in relation to the SW620 and SW480 lines (colorectal cancer) showed that the tested compounds exhibit high cytotoxicity towards the cells of these lines [36]. Similar results were obtained by Al Rasheed et al. [37] in their next study examining hepatocellular carcinoma (HepG2) and colon cancer (HCT116). The IC_50_ values of the best compound against the tested HepG2 and HCT–116 cell lines were 3.8 ± 0.3 and 1.9 ± 0.4 µg/mL, respectively. These results indicate that thiobarbiturate-based *s*-triazine hydrazones can be an excellent scaffold for development of an anticancer drug candidate [37].

## 4. Materials and Methods

### 4.1. Chemistry

The chemicals and solvents were purchased from the commercial sources Sigma-Aldrich Co. (St. Louis, MO, USA), Merck Co. (Darmstadt, Germany), Polish Chemical Reagents (Mielec, Poland) and used without further purification. In order to examine the purity of the obtained compounds as well as the progress of the reactions, thin-layer chromatography (TLC) on the aluminum plates covered with silica gel (aluminum oxide 60 F-254, Merck Co.) with the chloroform-ethanol mixture 10:1 (*v*/*v*) used as the mobile phase were applied. The spots on the chromatograms were detected by irradiation with UV light at a λ = 254 nm. The Bruker Avance 300 and 600 apparatus (Bruker BioSpin GmbH, Rheinstetten, Germany) were used to register the ^1^H NMR and ^13^C NMR spectra. The IR spectra of the obtained acylhydrazones and the samples after the recrystallization experiments were recorded on a Nicolet 6700 FT-IR spectrophotometer in the ATR mode. The Fisher-Johns apparatus (Fisher Scientific, Schwerte, Germany) was used to establish melting points of acylhydrazones.

### 4.2. Synthesis and Chemical Characterization

#### 4.2.1. Preparation of Hydrazides of 2-, 3-, or 4-Iodobenzoic Acid (**4**–**6**)

Methyl ester of 2-, 3-, or 4-iodobenzoic acid (CAS Number: 88-67-5, 618-51-9, 619-58-9) (0.001 mole) (**1**, **2**, **3**) was placed in a round-bottomed flask and dissolved in 2 mL of anhydrous ethanol (99.8%) by heating under reflux. After that 0.0011 mole of 100% hydrazine hydrate was added. The solution was heated under reflux for 3 h. Then, it was allowed to cool and was placed in a refrigerator for 24 h. The formed precipitate was filtered off under the reduced pressure and recrystallized from ethanol (96%).

##### Physico-Chemical Data of Hydrazides of 2-, 3-, or 4-Iodobenzoic Acid (**4**–**6**)

2-iodobenzohydrazide (**4**)

CAS Number: 88-67-5. Yield: 70%. Color: white powder. M.p.: 182–189 °C; IR (cm^−1^): 3298 (NH_2_), 3203 (NH), 3076, 3036 (CH, arom.), 1639 (C=O), 1621 (C=N); ^1^H NMR (600 MHz, DMSO–*d_6_*) δ (ppm): 4.51 (s, 2H, NH_2_), 7.16–7.19 (m, 1H, ArH), 7.28–7.29 (m, 1H, ArH), 7.42–7.45 (m, 1H, ArH), 7.87–7.89 (m, 1H, ArH), 9.52 (s, 1H, NH); ^13^C NMR (150 MHz, DMSO–*d_6_*) δ (ppm): 94.56, 128.39, 128.84, 131.35, 139.60, 142.11 (6C_ar_), 168.61 (C=O).

3-iodobenzohydrazide (**5**)

CAS Number: 618-51-9. Yield: 67%. Color: white powder. M.p.: 134–140 °C; IR (cm^−1^): 3312 (NH_2_), 3177 (NH), 3040 (CH, arom.), 1651 (C=O), 1618 (C=N); ^1^H NMR (600 MHz, DMSO–*d_6_*) δ (ppm): 4.53 (s, 2H, NH_2_), 7.25–7.28 (t, 1H, ArH, *J* = 12 Hz, *J* = 6 Hz), 7.82–7.89 (m, 2H, ArH), 8.15–8.16 (m, 1H, ArH), 9.86 (s, 1H, NH); ^13^C NMR (150 MHz, DMSO–*d_6_*) δ (ppm): 95.17, 126.77, 131.00, 135.79, 135.95, 140.07 (6C_ar_), 164.74 (C=O).

4-iodobenzohydrazide (**6**)

CAS Number: 619-58-9. Yield: 72%. Color: white powder. M.p.: 161–162 °C; IR (cm^−1^): 3294 (NH_2_), 3183 (NH), 3075, 3050 (CH, arom.), 1645 (C=O), 1624 (C=N); ^1^H NMR (600 MHz, DMSO–*d_6_*) δ (ppm): 4.54 (s, 2H, NH_2_), 7.59–7.61 (d, 2H, ArH, *J* = 12 Hz), 7.83–7.84 (d, 2H, ArH, *J* = 6 Hz), 9.84 (s, 1H, NH); ^13^C NMR (150 MHz, DMSO–*d_6_*) δ (ppm): 99.06, 129.39, 133.20, 137.67 (6C_ar_), 165.61 (C=O).

#### 4.2.2. Preparation of Novel 2-, 3-, or 4-Iodobenzoic Acid Hydrazide Derivatives—Acylhydrazones of 2-, 3-, or 4-Iodobenzoic Acid (**7**–**28**)

The 0.001 mole of 2-, 3-, or 4-iodobenzoic acid hydrazides (**4**, **5**, **6**) and 3 mL of anhydrous ethanol (99.8%) were added to the round-bottom flask and heated under reflux until the hydrazide dissolved. Next, 0.0011 mole of suitable aldehyde was added to the flask and the content of the flask was heated under reflux for 3 h. Subsequently, the solution was cooled under a jet of cold water and then placed in the refrigerator for 24 h. After that, the precipitate formed was filtered off under the reduced pressure, dried and re-crystallized from ethanol (96%).

##### Physico-Chemical Data of Novel Acylhydrazones of 2-, 3-, or 4-Iodobenzoic Acid (**7**–**28**)

*N*-[(2-hydroxy-3-nitrophenyl)methylidene]-2-iodobenzohydrazide (**7**)

Yield: 65%. Color: yellow powder. M.p.: 240–245 °C; IR (cm^−1^): 3192 (NH), 3090 (CH, arom.), 2851 (CH, aliph.), 1658 (C=O), 1615 (C=N); ^1^H NMR (600 MHz, DMSO–*d_6_*) δ (ppm): 7.12–7.13 (d, 1H, ArH, *J* = 6 Hz), 7.17–7.18 (d, 1H, ArH, *J* = 6 Hz), 7.22–7.29 (m, 1H, ArH), 7.50–7.55 (m, 1H, ArH), 7.96–7.97 (d, 1H, ArH, *J* = 6 Hz), 8.16–8.20 (m, 1H, ArH), 8.61–8.62 (d, 1H, ArH, *J* = 6 Hz), 8.59 (s, 1H, =CH), 9.10 (s, 1H, OH), 12.22 (s, 1H, NH); ^13^C NMR (150 MHz, DMSO–*d_6_*) δ (ppm): 94.52, 117.56, 120.52, 123.91, 127.21, 128.64, 129.10, 132.05, 139.71, 140.44 (10C_ar_), 144.52 (=CH), 160.46, 162.91 (2C_ar_), 165.49 (C=O).

*N*-[(2-hydroxyphenyl)methylidene]-2-iodobenzohydrazide (**8**)

Yield: 65%. Color: white powder. M.p.: 198–202 °C; IR (cm^−1^): 3222 (OH), 3195 (NH), 3168, 3059 (CH, arom.), 2834 (CH, aliph.), 1667 (C=O), 1652 (C=N); ^1^H NMR (600 MHz, DMSO–*d_6_*) δ (ppm): 6.78–6.81 (m, 1H, ArH), 6.92–6.96 (m, 1H, ArH), 7.19–7.23 (m, 1H, ArH), 7.25–7.29 (m, 1H, ArH), 7.30–7.36 (m, 1H, ArH), 7.48–7.54 (m, 1H, ArH), 7.57–7.58 (m, 1H, ArH), 7.96–7.97 (d, 1H, ArH, *J* = 6 Hz), 8.49 (s, 1H, =CH), 11.05 (s, 1H, OH), 12.07 (s, 1H, NH); ^13^C NMR (150 MHz, DMSO–*d_6_*) δ (ppm): 94.57, 116.87, 119.88, 128.63, 129.12, 129.69, 131.99, 139.70, 141.29, 144.46 (10C_ar_), 148.51 (=CH), 157.02, 157.83 (2C_ar_), 165.17 (C=O).

*N*-[(3,5-dichloro-2-hydroxyphenyl)methylidene]-2-iodobenzohydrazide (**9**)

Yield: 51%. Color: white powder. M.p.: 205–207 °C; IR (cm^−1^): 3299 (OH), 3192 (NH), 3072 (CH, arom.), 2966, 2847, 2606 (CH, aliph.), 1642 (C=O), 1623 (C=N); ^1^H NMR (600 MHz, DMSO–*d_6_*) δ (ppm): 7.24–7.30 (m, 1H, ArH), 7.38–7.40 (m, 1H, ArH), 7.52–7.55 (m, 1H, ArH), 7.74–7.75 (d, 1H, ArH, *J* = 6 Hz), 7.78–7.79 (d 1H, ArH, *J* = 6 Hz), 7.97–7.98 (d, 1H, ArH, *J* = 6 Hz), 8.45 (s, 1H, =CH), 9.09 (s, 1H, OH), 11.83 (s, 1H, NH); ^13^C NMR (150 MHz, DMSO–*d_6_*) δ (ppm): 94.50, 120.76, 121.30, 122.46, 123.83, 128.77, 130.00, 130.94, 132.91, 139.77 (10C_ar_), 147.54 (=CH), 152.63, 153.79 (2C_ar_), 164.02 (C=O).

*N*-[(3-ethoxy-2-hydroxyphenyl)methylidene]-2-iodobenzohydrazide (**10**)

Yield: 82%. Color: yellowish powder. M.p.: 167–170 °C; IR (cm^−1^): 3171 (OH), 3158 (NH), 3059 (CH arom.), 2980 (CH, aliph.) 1621 (C=O), 1574 (C=N); ^1^H NMR (600 MHz, DMSO–*d_6_*) δ (ppm): 1.35–1.37 (t, 3H, CH_3_, *J* = 6 Hz), 4.06–4.10 (q, 2H, CH_2_, *J* = 6 Hz, *J* = 12 Hz), 6.72–6.74 (t, 1H, ArH, *J* = 6 Hz), 6.84–6.87 (t, 1H, ArH, *J* = 6 Hz, *J* = 12 Hz), 7.03–7.05 (m, 1H, ArH), 7.16–7.18 (m, 1H, ArH), 7.21–7.29 (m, 1H, ArH), 7.49–7.54 (m, 1H, ArH), 7.95–7.97 (d, 1H, ArH, *J* = 12 Hz), 8.50 (s, 1H, =CH), 10.65 (s, 1H, OH), 12.05 (s, 1H, NH); ^13^C NMR (150 MHz, DMSO–*d_6_*) δ (ppm): 15.21 (CH_3_), 64.68 (CH_2_), 94.57, 115.86, 119.60, 121.15, 128.62, 129.13, 131.98, 139.70, 141.31, 144.67, 147.51, 147.88 (12C_ar_), 148.45 (=CH), 165.13 (C=O).

*N*-[(2,5-dihydroxyphenyl)methylidene]-2-iodobenzohydrazide (**11**)

Yield: 79%. Color: green powder. M.p.: 211–214 °C; IR (cm^−1^): 3254 (OH), 3207 (NH), 3169, 3068 (CH arom.) 1655 (C=O), 1614 (C=N); ^1^H NMR (600 MHz, DMSO–*d_6_*) δ (ppm): 6.61–6.65 (m, 1H, ArH), 6.73–6.77 (m, 1H, ArH), 6.97–7.01 (m, 1H, ArH), 7.19–7.27 (m, 1H, ArH), 7.47–7.53 (m, 1H, ArH), 7.95–7.96 (d, 1H, ArH, *J* = 6 Hz), 8.22 (s, 1H, ArH), 8.42 (s, 1H, =CH), 9.00 (s, 1H, OH), 10.17 (s, 1H, OH), 11.95 (s, 1H, NH); ^13^C NMR (150 MHz, DMSO–*d_6_*) δ (ppm): 94.57, 113.94, 117.56, 119.61, 128.61, 129.09, 131.91, 139.67, 141.44, 144.21 (10C_ar_), 147.86 (=CH), 150.38, 150.61 (2C_ar_), 165.14 (C=O).

*N*-[(5-chloro-2-hydroxy-3-iodophenyl)methylidene]-2-iodobenzohydrazide (**12**)

Yield: 70%. Color: yellowish powder. M.p.: 215–220 °C; IR (cm^−1^): 3290 (OH), 3153 (NH), 3068 (CH arom.), 2992 2953 (CH, aliph.), 1654 (C=O), 1621 (C=N); ^1^H NMR (600 MHz, DMSO–*d_6_*) δ (ppm): 7.26–7.31 (m, 1H, ArH), 7.52–7.55 (m, 1H, ArH), 7.72–7.73 (d, 1H, ArH, *J* = 6 Hz), 7.87–7.88 (d, 1H, ArH, *J* = 6 Hz), 7.97–7.99 (d, 1H, ArH, *J* = 12 Hz), 8.17 (s, 1H, ArH), 8.35 (s, 1H, =CH), 9.05 (s, 1H, OH), 12.71 (s, 1H, NH); ^13^C NMR (150 MHz, DMSO–*d_6_*) δ (ppm): 87.24, 94.51, 119.31, 124.21, 129.18, 130.71, 132.26, 139.31, 139.78, 140.59 (10C_ar_), 148.05 (=CH), 156.23, 157.38 (2C_ar_), 165.39 (C=O).

*N*-[(3,5-dibromo-2-hydroxyphenyl)methylidene]-2-iodobenzohydrazide (**13**)

Yield: 83%. Color: yellowish powder. M.p.: 220–222 °C; IR (cm^−1^): 3250 (OH), 3150 (NH), 3073 (CH arom.), 2992, 2958, 2822 (CH aliph.), 1653 (C=O), 1610 (C=N); ^1^H NMR (600 MHz, DMSO–*d_6_*) δ (ppm): 7.25–7.31 (m, 1H, ArH), 7.52–7.54 (m, 1H, ArH), 7.83–7.84 (d, 1H, ArH, *J* = 6 Hz), 7.86–7.87 (d, 1H, ArH, *J* = 6 Hz), 7.97–7.98 (d, 1H, ArH, *J* = 6 Hz), 8.23 (s, 1H, ArH), 8.42 (s, 1H, =CH), 9.09 (s, 1H, OH), 12.54 (s, 1H, NH); ^13^C NMR (150 MHz, DMSO–*d_6_*) δ (ppm): 94.51, 111.01, 111.79, 121.42, 128.65, 129.18, 132.25, 132.59, 136.25, 139.78, 140.62 (11C_ar_), 147.76 (=CH), 154.06 (C_ar_), 165.40 (C=O).

*N*-[(3,5-diiodo-2-hydroxyphenyl)methylidene]-2-iodobenzohydrazide (**14**)

Yield: 85%. Color: brown powder. M.p.: 241–245 °C; IR (cm^−1^): 3647 (OH), 3168 (NH), 3045 (CH, arom.), 2990 (CH, aliph.), 1655 (C=O), 1611 (C=N); ^1^H NMR (600 MHz, DMSO–*d_6_*) δ (ppm): 7.25–7.31 (m, 1H, ArH), 7.52–7.55 (m, 1H, ArH), 7.65–7.66 (d, 1H, ArH, *J* = 6 Hz), 7.69–7.70 (d, 1H, ArH, *J* = 6 Hz), 7.97–7.98 (d, 1H, ArH, *J* = 6 Hz), 8.26 (s, 1H, ArH), 8.45 (s, 1H, =CH), 12.25 (s, 1H, OH), 12.50 (s, 1H, NH); ^13^C NMR (150 MHz, DMSO–*d_6_*) δ (ppm): 94.50, 121.29, 122.07, 123.55, 128.65, 128.78, 129.18, 130.94, 132.23, 139.77, 140.68 (11C_ar_), 147.54 (=CH), 152.64 (C_ar_), 165.40 (C=O).

*N*-[(2-hydroxy-3-nitrophenyl)methylidene]-3-iodobenzohydrazide (**15**)

Yield: 80%. Color: yellow powder. M.p.: 190–199 °C; IR (cm^−1^): 3586 (OH), 3324 (NH), 3079 (CH, arom.), 2849 (CH, aliph.), 1684 (C=O), 1615 (C=N); ^1^H NMR (600 MHz, DMSO–*d_6_*) δ (ppm): 7.13–7.16 (t, 1H, ArH, *J* = 12 Hz, *J* = 6 Hz), 7.17–7.20 (t, 1H, ArH, *J* = 12 Hz, *J* = 6 Hz), 7.37–7.40 (t, 1H, ArH, *J* = 6 Hz, *J* = 12 Hz), 7.96–7.98 (m, 1H, ArH), 8.00–8.06 (m, 2H, ArH), 8.26–8.30 (m, 1H, ArH), 8.72 (s, 1H, =CH), 8.72 (s, 1H, OH), 10.30 (s, 1H, NH); ^13^C NMR (150 MHz, DMSO–*d_6_*) δ (ppm): 95.39, 119.76, 127.20, 127.78, 131.30, 134.86, 135.48, 136.39, 138.10, 141.26 (10C_ar_), 147.65 (=CH), 152.06, 162.02 (2C_ar_), 191.59 (C=O).

*N*-[(2-hydroxyphenyl)methylidene]-3-iodobenzohydrazide (**16**) [38]

Physico-chemical properties of this compound are consistent with those reported earlier [38]. Yield: 57%. Color: white crystals. M.p.: 201–203 °C; IR (cm^−1^): 3369 (OH), 3219 (NH), 3062 (CH arom.), 2858 (CH aliph.), 1619 (C=O), 1605 (C=N); ^1^H NMR (600 MHz, DMSO–*d_6_*) δ (ppm): 6.92–6.95 (m, 2H, ArH), 7.32–7.33 (m, 1H, ArH), 7.35–7.38 (t, 1H, ArH, *J* = 12 Hz, *J* = 6 Hz), 7.57–7.58 (m, 1H, ArH), 7.95–7.99 (m, 2H, ArH), 8.29–8.30 (m, 1H, ArH), 8.65 (s, 1H, =CH), 11.18 (s, 1H, OH), 12.15 (s, 1H, NH); ^13^C NMR (150 MHz, DMSO–*d_6_*) δ (ppm): 95.29, 116.90, 119.18, 119.86, 127.67, 129.80. 131.22, 132.01, 135,37, 136,31, 140.94 (11C_ar_), 148.92 (=CH), 157.93 (C_ar_), 161.76 (C=O).

*N*-[(3,5-dichloro-2-hydroxyphenyl)methylidene]-3-iodobenzohydrazide (**17**)

Yield: 91%. Color: white powder. M.p.: 210–212 °C; IR (cm^−1^): 3207 (OH), 3099 (NH), 3049, (CH, arom.), 2838 (CH, aliph.), 1640 (C=O), 1610 (C=N); ^1^H NMR (600 MHz, DMSO–*d_6_*) δ (ppm): 7.36–7.39 (t, 1H, ArH, *J* = 12 Hz, *J* = 6 Hz), 7.64–7.65 (d, 1H, ArH, *J* = 6 Hz), 7.69–7.70 (d, 1H, ArH, *J* = 6 Hz), 7.96–7.98 (m, 1H, ArH), 8.00–8.01 (m, 1H, ArH), 8.29–8.30 (m, 1H, ArH), 8.57 (s, 1H, =CH), 12.41 (s, 1H, OH), 12.55 (s, 1H, NH); ^13^C NMR (150 MHz, DMSO–*d_6_*) δ (ppm): 95.36, 121.26, 122.02, 123.47, 127.80, 128.86, 130.87, 131.27, 134.72, 136.41, 141.29 (11C_ar_), 147.79 (=CH), 152.73 (C_ar_), 162.03 (C=O).

*N*-[(3-ethoxy-2-hydroxyphenyl)methylidene]-3-iodobenzohydrazide (**18**)

Yield: 88%. Color: white powder. M.p.: 115–121 °C; IR (cm^−1^): 3416 (OH), 3219 (NH), 3077 (CH, arom.), 2991, 2925 (CH, aliph.), 1639 (C=O), 1604 (C=N); ^1^H NMR (600 MHz, DMSO–*d_6_*) δ (ppm): 1.35–1.37 (t, 3H, CH_3_, *J* = 6 Hz), 4.05–4.09 (q, 2H, CH_2_, *J* = 6 Hz), 6.84–6.87 (t, 1H, ArH, *J* =6 Hz, *J* = 12 Hz), 7.02–7.04 (m, 1H, ArH), 7.15–7.16 (m, 1H, ArH), 7.35–7.37 (t, 1H, ArH, *J* = 6 Hz), 7.95–7.99 (m, 2H, ArH), 8.29–8.30 (m, 1H, ArH), 8.66 (s, 1H, =CH), 10.83 (s, 1H, OH), 12.13 (s, 1H, NH); ^13^C NMR (150 MHz, DMSO–*d_6_*) δ (ppm): 15.22 (CH_3_), 64.61 (CH_2_), 95.30, 115.74, 119.47, 119.56, 121.30, 127.67, 131.21, 135.36, 136.32, 140.93 (10C_ar_), 147.55 (=CH), 147.95, 148.99 (2C_ar_), 161.72 (C=O).

*N*-[(2,5-dihydroxyphenyl)methylidene]-3-iodobenzohydrazide (**19**)

Yield: 75%. Color: yellowish powder. M.p.: 220–224 °C; IR (cm^−1^): 3400 (OH), 3208 (NH), 3065 (CH, arom.), 2917, 2851 (CH, aliph.), 1627 (C=O), 1616 (C=N); ^1^H NMR (600 MHz, DMSO–*d_6_*) δ (ppm): 6.73–6.77 (m, 2H, ArH), 7.01–7.02 (d, 1H, ArH, *J* = 6 Hz), 7.34–7.37 (t, 1H, ArH, *J* = 6 Hz, *J* = 12 Hz), 7.94–7.98 (m, 2H, ArH), 8.28–8.29 (m, 1H, ArH), 8.58 (s, 1H, =CH), 9.01 (s, 1H, OH), 10.28 (s, 1H, OH), 12.03 (s, 1H, NH); ^13^C NMR (150 MHz, DMSO–*d_6_*) δ (ppm): 95.29, 114.01, 117.51, 119.52, 119.56, 127.65, 131.19, 135.49, 136.30, 140.85 (10C_ar_), 148.27 (=CH), 150.37, 150.69 (2C_ar_), 161.69 (C=O).

*N*-[(5-chloro-2-hydroxy-3-iodophenyl)methylidene]-3-iodobenzohydrazide (**20**)

Yield: 84%. Color: white powder. M.p.: 205–208 °C; IR (cm^−1^): 3247 (OH), 3174 (NH), 3011 (CH arom.), 2849 (CH aliph.), 1641 (C=O), 1605 (C=N); ^1^H NMR (600 MHz, DMSO–*d_6_*) δ (ppm): 7.36–7.39 (t, 1H, ArH, *J* = 12 Hz, *J* = 6 Hz), 7.70 (m, 1H, ArH), 7.85 (m, 1H, ArH), 7.96–7.97 (m, 1H, ArH), 7.99–8.01 (m, 1H, ArH), 8.29–8.30 (m, 1H, ArH), 8.46 (s, 1H, =CH), 12.59 (s, 1H, OH), 12.82 (s, 1H, NH); ^13^C NMR (150 MHz, DMSO–*d_6_*) δ (ppm): 87.19, 95.34, 119.34, 124.13, 127.78, 130.66, 131.25, 134.63, 136.40, 139.20, 141.29 (11C_ar_), 148.14 (=CH), 156.27 (C_ar_), 161.89 (C=O).

*N*-[(3,5-dibromo-2-hydroxyphenyl)methylidene]-3-iodobenzohydrazide (**21**)

Yield: 80%. Color: white powder. M.p.: 210–211 °C; IR (cm^−1^): 3182 (NH), 3049 (CH arom.), 2829 (CH aliph.), 1640 (C=O), 1609 (C=N); ^1^H NMR (600 MHz, DMSO–*d_6_*) δ (ppm): 7.36–7.39 (t, 1H, ArH, *J* = 12 Hz, *J* = 6 Hz), 7.83–7.84 (d, 1H, ArH, *J* = 6 Hz), 7.84–7.85 (d, 1H, ArH. *J* = 6 Hz), 7.96–7.97 (m, 1H, ArH), 8.00–8.01 (m, 1H, ArH), 8.29–8.30 (m, 1H, ArH), 8.53 (s, 1H, =CH), 12.59 (s, 1H, OH), 12.63 (s, 1H, NH); ^13^C NMR (150 MHz, DMSO–*d_6_*) δ (ppm): 95.34, 110.91, 111.74, 121.42, 127.79, 131.26, 132.59, 134.67, 136.16, 136.41, 141.29 (11C_ar_), 147.92 (=CH), 154.13 (C_ar_), 162.03 (C=O).

*N*-[(2-hydroxy-3-nitrophenyl)methylidene]-4-iodobenzohydrazide (**22**)

Yield: 85%. Color: yellow powder. M.p.: 198–202 °C; IR (cm^−1^): 3292 (NH), 3081, 3065 (CH, arom.), 2853 (CH, aliph.), 1660 (C=O), 1617 (C=N); ^1^H NMR (600 MHz, DMSO–*d_6_*) δ (ppm): 7.12–7.15 (t, 1H, ArH, *J* = 12 Hz, *J* = 6 Hz), 7.74–7.75 (d, 2H, ArH, *J* = 6 Hz), 7.94–7.95 (m, 1H, ArH), 7.96–7.97 (d, 2H, ArH, *J* = 6 Hz), 8.02–8.05 (m, 1H, ArH), 8.72 (s, 1H, =CH), 10.30 (s, 1H, OH), 12.48 (s, 1H, NH); ^13^C NMR (150 MHz, DMSO–*d_6_*) δ (ppm): 100.74, 119.69, 122.15, 127.12, 130.03, 132.12, 135.50, 136.34, 138.00 (11C_ar_), 147.58 (=CH), 152.06 (C_ar_), 162.84 (C=O).

*N*-[(2-hydroxyphenyl)methylidene]-4-iodobenzohydrazide (**23**)

Yield: 82%. Color: white powder. M.p.: 195–205 °C; IR (cm^−1^): 3378 (OH), 3208 (NH), 3033 (CH, arom.), 2865 (CH, aliph.), 1647 (C=O), 1618 (C=N); ^1^H NMR (600 MHz, DMSO–*d_6_*) δ (ppm): 6.92–6.95 (m, 2H, ArH), 7.30–7.33 (m, 1H, ArH), 7.56–7.57 (m, 1H, ArH), 7.72–7.74 (d, 2H, ArH, *J* = 12 Hz), 7.94–7.96 (d, 2H, ArH, *J* = 12 Hz), 8.65 (s, 1H, =CH), 11.22 (s, 1H, OH), 12.15 (s, 1H, NH); ^13^C NMR (150 MHz, DMSO–*d_6_*) δ (ppm): 100.33, 116.90, 119.16, 119.85, 129.89, 129.99, 131.97, 132.66, 137.93 (11C_ar_), 148.88 (=CH), 157.93 (C_ar_), 162.62 (C=O).

*N*-[(3,5-dichloro-2-hydroxyphenyl)methylidene]-4-iodobenzohydrazide (**24**)

Yield: 90%. Color: white powder. M.p.: 281–284 °C; IR (cm^−1^): 3214 (OH), 3176 (NH), 3085, 3047 (CH, arom.), 2838 (CH, aliph.), 1653 (C=O), 1607 (C=N); ^1^H NMR (600 MHz, DMSO–*d_6_*) δ (ppm): 7.64–7.65 (d, 1H, ArH, *J* = 6 Hz), 7.69–7.70 (d, 1H, ArH, *J* = 6 Hz), 7.74–7.75 (d, 2H, ArH, *J* = 6 Hz), 7.96–7.97 (d, 2H, ArH, *J* = 6 Hz), 8.58 (s, 1H, =CH), 12.46 (s, 1H, OH), 12.57 (s, 1H, NH); ^13^C NMR (150 MHz, DMSO–*d_6_*) δ (ppm): 100.85, 121.24, 121.98, 123.44, 128.91, 130.08, 130.83, 132.01, 138.01 (11C_ar_), 147.68 (=CH), 152.74 (C_ar_), 162.88 (C=O).

*N*-[(3-ethoxy-2-hydroxyphenyl)methylidene]-4-iodobenzohydrazide (**25**)

Yield: 87%. Color: white powder. M.p.: 200–207 °C; IR (cm^−1^): 3212 (OH), 3201 (NH), 3082, 3053 (CH, arom.), 2977, 2930 (CH, aliph.), 1648 (C=O), 1607 (C=N); ^1^H NMR (600 MHz, DMSO–*d_6_*) δ (ppm): 1.35–1.37 (t, 3H, CH_3_, *J* = 6 Hz), 4.05–4.09 (q, 2H, CH_2_, *J* = 6 Hz), 6.84–6.87 (t, 1H, ArH, *J* = 6 Hz, *J* = 12 Hz), 7.02–7.04 (m, 1H, ArH), 7.14–7.16 (m, 1H, ArH), 7.73–7.74 (d, 2H, ArH, *J* = 6 Hz), 7.94–7.95 (d, 2H, ArH, *J* = 6 Hz), 8.65 (s, 1H, =CH), 10.89 (s, 1H, OH), 12.13 (s, 1H, NH); ^13^C NMR (150 MHz, DMSO–*d_6_*) δ (ppm): 15.23 (CH_3_), 64.60 (CH_2_), 100.32, 115.73, 119.45, 119.55, 121.38, 130.00, 132.67, 137.92, 147.54, 147.95 (12C_ar_), 148.91 (=CH), 162.57 (C=O).

*N*-[(2,5-dihydroxyphenyl)methylidene]-4-iodobenzohydrazide (**26**)

Yield: 79%. Color: yellowish powder. M.p.: 261–265 °C; IR (cm^−1^): 3330 (OH), 3242 (NH), 3054 (CH, arom.), 2873 (CH, aliph.), 1609 (C=O), 1582 (C=N); ^1^H NMR (600 MHz, DMSO–*d_6_*) δ (ppm): 6.73–6.77 (m, 2H, ArH), 6.99–7.00 (d, 1H, ArH, *J* = 6 Hz), 7.71–7.73 (d, 2H, ArH, *J* = 12 Hz), 7.93–7.95 (d, 2H, ArH, *J* = 12 Hz), 8.58 (s, 1H, =CH), 9.00 (s, 1H, OH), 10.32 (s, 1H, OH), 12.03 (s, 1H, NH); ^13^C NMR (150 MHz, DMSO–*d_6_*) δ (ppm): 100.20, 114.09, 117.55, 119.50, 119.51, 129.98, 132.81, 137.89 (10C_ar_), 148.19 (=CH), 150.36, 150.68 (2C_ar_), 162.55 (C=O).

*N*-[(5-chloro-2-hydroxy-3-iodophenyl)methylidene]-4-iodobenzohydrazide (**27**)

Yield: 87%. Color: yellowish powder. M.p.: 200–201 °C; IR (cm^−1^): 3220 (NH), 3055 (CH arom.), 2831 (CH aliph.), 1654 (C=O), 1602 (C=N); ^1^H NMR (600 MHz, DMSO–*d_6_*) δ (ppm): 7.74–7.75 (d, 2H, ArH, *J* = 6 Hz), 7.85–7.86 (m, 1H, ArH), 7.96–7.98 (d, 2H, ArH, *J* = 12 Hz), 8.14–8.15 (d, 1H, ArH, *J* = 6 Hz), 8.47 (s, 1H, =CH), 9.95 (s, 1H, OH), 12.87 (s, 1H, NH); ^13^C NMR (150 MHz, DMSO–*d_6_*) δ (ppm): 87.18, 89.33, 100.85, 119.38, 122.55, 124.12, 124.97, 130.07, 132.07, 138.01, 144.34 (11C_ar_), 147.88 (=CH), 158.55 (C_ar_), 162.85 (C=O).

*N*-[(3,5-dibromo-2-hydroxyphenyl)methylidene]-4-iodobenzohydrazide (**28**)

Yield: 80%. Color: white powder. M.p.: 210–211 °C; IR (cm^−1^): 3219 (NH), 3049 (CH arom.), 2829 (CH aliph.), 1653 (C=O), 1604 (C=N); ^1^H NMR (600 MHz, DMSO–*d_6_*) δ (ppm): 7.59–7.61 (d, 1H, ArH, *J* = 18 Hz), 7.73–7.74 (d, 2H, ArH, *J* = 12 Hz), 7.81–7.83 (m, 1H, ArH), 7.94–7.96 (d, 2H, ArH, *J* = 12 Hz), 8.52 (s, 1H, =CH), 9.85 (s, 1H, OH), 12.59 (s, 1H, NH); ^13^C NMR (150 MHz, DMSO–*d_6_*) δ (ppm): 100.82, 110.86, 111.70, 121.39, 129.38, 130.05, 131.93, 132.59, 136.08, 137.64, 137.97 (11C_ar_), 147.74 (=CH), 154.13 (C_ar_), 162.82 (C=O).

### 4.3. Mechanochemistry

The mechanochemical synthesis of hydrazones **13**, **14**, **24** and **26** was conducted by neat (NG) and liquid-assisted grinding (LAG) of the respective substrates (hydrazides and aldehydes) in a ball mill (MM200, Retsch, Haan, Germany) at 30 Hz for 90 min. In the case of compound **24**, shorter grinding times, i.e., 60 and 30 min, were also tested. The 10 mL steel vessels with two steel balls (5 mm diameter) were used for a total quantity of 80 mg (1:1 molar ratio) of the reactants. For LAG, 20 μL of pure ethanol (99.9%) or acetonitrile was added. In all experiments, the product was recovered by scratching out the powder from the jar without further treatment. The identity of the final products was assessed comparing the experimental powder patterns of the starting materials, the products, and the theoretical powder patterns of the expected hydrazones, calculated from the SCXRD data.

### 4.4. Crystallization Experiments

Single crystals of the reported hydrazones (Table 1), suitable for the X-ray diffraction studies, were obtained by recrystallization of the resulting samples from common organic solvents, such as methanol, ethanol (99.9%), acetonitrile and chloroform. Solution-crystallization experiments were conducted by dissolving the sample in the minimum amount of the appropriate solvent mixing at 55–65 °C until a clear solution was obtained. Then, the solutions were cooled to room temperature and allowed to evaporate slowly under the ambient conditions.

### 4.5. X-ray Crystallography

The single-crystal X-ray diffraction measurements were conducted at 293(2) K using the Rigaku XtaLAB MM7HFMR diffractometer equipped with the “quarter-chi single” goniometer, the rotating anode generator (graphite monochromated Cu Kα radiation, *λ* = 1.5418 Å) and the Pilatus 200K detector. The *CrysAlisPro* 1.171.40.45a software was used for data collection, cell refinement, and data reduction [39]. The multi-scan absorption correction was applied. The structures were solved using the direct methods implemented in the *SHELXS*-*97* program and were refined with the *SHELXL*-*2018/3*, [40] both operating under *WinGX* 2020.2 [41]. All non-H atoms were refined with anisotropic displacement parameters. The hydrogen atoms attached to water molecules, hydrazide and hydroxyl groups were found from the difference Fourier maps and, where possible, refined with the isotropic displacement parameters. All remaining H-atoms were positioned geometrically and refined using the riding model with *U_iso_*(H) = 1.2*U_eq_*(CH, CH_2_) or *U_iso_*(H) = 1.5*U_eq_*(CH_3_). The *Mercury 2022.3.0* [42] software was used for crystal structure analysis, preparation of the molecular plots and simulation of powder patterns, based on the SCXRD data.

CCDC Nos 2357466-2357475 contain the supplementary crystallographic data for the studied structures (Table 1). Copies of the data can be obtained free of charge based on application to CCDC, 12 Union Road, Cambridge CB21EZ, UK (https://www.ccdc.cam.ac.uk/ or e-mail: deposit@ccdc.cam.ac.uk).

### 4.6. Powder X-ray Diffraction

Powder X-ray diffraction data were collected using the PANalytical Empyrean automated diffractometer with the Bragg-Brentano geometry (Malvern Panalytical, Malvern, UK) using Ni-filtered Cu Kα radiation (*λ* = 1.5418 Å). The intensity data were registered at room temperature with the PIXcel^3D^ detector (Malvern Panalytical, Malvern, UK) over the 2*θ* range of 3−50°, with the step size of Δθ = 0.013° and the scan speed of 0.0847°/s. The overlays of diffraction patterns were generated using the *HighScore Plus* 3.0e software [43].

### 4.7. Microbiology

#### In Vitro Antimicrobial Assay

The in vitro antibacterial and antifungal activities for synthesized compounds **4**–**6**, **7**–**14**, **15**–**21**, **22**–**28** were established with the use of the broth microdilution method according to the procedures recommended by the European Committee on Antimicrobial Susceptibility Testing (EUCAST) [44] and Clinical and Laboratory Standards Institute [45] against a panel of reference and clinical or saprophytic strains of microorganisms. Procedures of in vitro antimicrobial assays are described in Supplementary Materials.

### 4.8. Cytotoxicity Study

#### 4.8.1. In Vitro Cytotoxicity Assay

Among all the newly synthesized compounds, four compounds designated as: **14**, **20**, **21** and **27** were selected for further testing of their cytotoxic properties at the doses of 10, 25, 50, 75 and 100 µM. The cytotoxic properties of the above compounds were tested on the cancer cell lines, i.e., A549 (ECACC 86012804) (non-small-cell lung cancer), T47D (ECACC 85102201) (breast cancer), HeLa (ATCC^®^ CCL-2™) (cervical cancer) and normal line L929 (NCTC clone 929, ATCC^®^ CCL-1™) (mouse fibroblasts) using the MTT test for 24- and 48 h cultures.

#### 4.8.2. Cell Lines

A549 (ECACC 86012804) (non-small-cell lung cancer) and T47D (ECACC 85102201) (breast cancer) were obtained from ECACC and propagated in Dulbecco’s Minimal Essential Medium, supplemented with 2 mM of glutamine, antibiotics (100 mg/mL streptomycin, 1% of 100 U/L penicillin) and 10% Fetal Bovine Serum. HeLa (ATCC^®^ CCL-2™) (cervical cancer) and L929 (NCTC clone 929, ATCC^®^ CCL-1™) (mouse fibroblasts) cell lines were obtained from ATCC and propagated in the Eagle Minimal Essential Medium supplemented with antibiotics (100 mg/mL streptomycin, 1% of 100 U/L penicillin) and with 10% and 5% FBS, respectively. The cell line was routinely grown in 75 cm^2^ tissue culture flasks and kept in an incubator (humidified atmosphere of 5% CO_2_ at 37 °C). All examined cell lines were tested against mycoplasma contamination with microbiological assays.

#### 4.8.3. MTT Analysis

The cell viability of tested compounds (**14**, **20**, **21** and **27**) was assessed by the MTT assay. In brief, the exponentially growing cells were plated in 100 μL/well in 96-well plates. After 24 h culturing, the cells were treated with different concentrations of compounds (range 10–100 μM). After 24 and 48 h of incubation, 10 μL of 5 mg/mL MTT was added to each well and the plates were incubated at 37 °C for additional 4 h. The medium was subsequently removed, the purple-colored precipitates of formazan were dissolved in 100 μL of DMSO. The color absorbance was recorded at 570 nm using a BioTek model EPOCH ELISA plate reader. All experiments were performed in triplicate. The absorbance values were in the range of linearity of the Lambert–Beer law.

## 5. Conclusions

In this research, the chemical structure of obtained acylhydrazones of 2-, 3- or 4-iodobenzoic was confirmed with the use of spectral analysis and X-ray crystallography. In addition, it was possible to synthesize the selected acylhydrazones with the use of mechanochemical synthesis.

The antimicrobial activity results proved that some of the newly synthesized compounds indicated a favorable antimicrobial effect, even against MRSA—methicillin-resistant *Staphylococcus aureus* ATCC 43300 strain. In many cases, their antibacterial activity was equal or higher than that of the reference substances, especially against the reference Gram-positive bacterial strains. The antibacterial activity of synthesized acylhydrazones was dependent on the nature of substituents present in their chemical structure. The widest spectrum and highest antimicrobial activity was shown by acylhydrazones of 3-iodobenzoic acid which were substituted with 5-chloro-2-hydroxy-3-iodophenyl (**20**) (towards Gram-positive and Gram-negative bacteria as well as fungi), 3,5-dibromo-2-hydroxyphenyl (**21**) (against Gram-positive bacteria and fungi) and acylhydrazone of 4-iodobenzoic acid which was substituted with 5-chloro-2-hydroxy-3-iodophenyl (**27**) (towards Gram-positive and Gram-negative bacteria as well as fungi). Among Gram-positive bacteria, the most susceptible to the tested acylhydrazones were *Staphylococcus epidermidis* ATCC 12228 and *Micrococcus luteus* ATCC 10240 strains, whereas among fungi, those were *Candida albicans* ATCC 10231and *Candida parapsilosis* ATCC 22019.

Due to these facts, it seems practical to use these compounds with further structure modifications in the future for prevention and treatment of infections, particularly caused by the Gram-positive bacteria and yeasts. Moreover, the tested compounds did not show toxicity to normal cell lines, which is very desirable in designing bioactive compounds.

## Figures and Tables

**Table 1:** Selected crystal data and experimental details for the studied acylhydrazones (more details in Supplementary Materials).

Crystal Structure	9	12	13	13∙ACN	14
Chemical formula	C_14_H_9_N_2_O_2_ICl_2_	C_14_H_9_N_2_O_2_I_2_Cl	C_14_H_9_N_2_O_2_IBr_2_	C_14_H_9_N_2_O_2_IBr_2_ ∙CH_3_CN	C_14_H_9_N_2_O_2_I_3_
Formula weight	435.03	526.48	523.95	565.01	617.93
Crystal system	monoclinic	monoclinic	monoclinic	orthorhombic	monoclinic
Space group	*P*2_1_/*c*	*P*2_1_/*n*	*P*2_1_/*c*	*Pbca*	*P*2_1_/*c*
*a*/Å	18.7006(4)	18.9192(4)	18.9820(7)	7.2352(1)	19.4686(3)
*b*/Å	9.5210(2)	9.5250(1)	9.5183(3)	22.9495(2)	9.6874(1)
*c*/Å	20.2936(4)	20.7505(4)	20.4590(6)	23.2354(3)	20.4776(3)
*β*/°	115.229(2)	116.442(3)	115.753(4)	90	115.951(2)
*V*/Å^3^	3268.6(1)	3348.2(1)	3329.3(2)	3858.10(8)	3472.7(1)
*Z/Z’*	8/2	8/2	8/2	8/1	8/2
*d*_calc/_g·cm^−3^	1.768	2.089	2.091	1.945	2.364
*R*_1_ [*F*^2^ > 2*σ*(*F*^2^)]	0.060	0.060	0.053	0.048	0.055
*wR*_2_ (all data)	0.170	0.171	0.138	0.123	0.157
CCDC number	2357466	2357467	2357468	2357469	2357470
**Crystal Structure**	**20**	**24**	**26a**	**26b**	**26c**
Chemical formula	C_14_H_9_N_2_O_2_I_2_Cl H_2_O	C_14_H_9_N_2_O_2_ICl_2_	C_14_H_11_N_2_O_3_I	C_14_H_11_N_2_O_3_I	C_14_H_11_N_2_O_3_I
Formula weight	544.50	435.03	382.15	382.15	382.15
Crystal system	monoclinic	monoclinic	orthorhombic	monoclinic	monoclinic
Space group	*P*2_1_/*c*	*C*2/*c*	*Pca*2_1_	*P*2_1_	*P*2_1_
*a*/Å	15.3068(3)	21.3618(3)	33.8455(4)	4.5303(1)	4.8826(1)
*b*/Å	7.6017(1)	9.9728(2)	4.7071(1)	10.5368(2)	5.0674(1)
*c*/Å	15.2558(3)	29.6378(4)	8.6261(1)	14.3691(3)	27.2710(4)
*β*/°	110.106(2)	101.705(1)	90	90.737(2)	90.047(1)
*V*/Å^3^	1666.95(5)	6182.7(2)	1374.26(4)	685.85(2)	674.74(2)
*Z/Z’*	4/1	16/2	4/1	2/1	2/1
*d*_calc/_g·cm^−3^	2.170	1.869	1.847	1.850	1.881
*R*_1_ [*F*^2^ > 2σ(*F*^2^)]	0.043	0.036	0.029	0.031	0.038
*wR*_2_ (all data)	0.118	0.095	0.073	0.082	0.101
CCDC number	2357471	2357472	2357473	2357474	2357475

**Table 2:** The activity data of studied compounds **4**, **7**–**14** expressed as MIC (MBC or MFC) [µg/mL] and {MBC/MIC or MFC/MIC} values against the reference strains of bacteria and fungi.

Species		MIC (MBC or MFC) [µg/mL] and {MBC/MIC or MFC/MIC} Values of the Studied Compounds and Positive Controls									
		4	7	10	11	12	14	CIP/NY*	NIT	CFX	APE
**Gram-positive bacteria**	*Staphylococcus* *aureus* ATCC 25923	500 (>1000) {>2}	500 (>1000) {>2}	–	–	125 (>1000) {>8}	125 (250) {2}	0.48 (0.48) {1}	15.62 (15.62)	0.49	nd
	*Staphylococcus* *aureus* ATCC 29213	–	–	–	–	1000 (>1000) {>1}	125 (500) {4}	0.48 (0.48) {1}	nd	nd	nd
	*Staphylococcus* *aureus* ATCC 43300	–	–	–	–	1000 (>1000) {>1}	250 (1000) {4}	0.24 (0.24) {1}	7.81	nd	nd
	*Staphylococcus* *epidermidis* ATCC 12228	1000 (>1000) {>1}	125 (1000) {8}	–	–	250 (500) {2}	125 (250) {2}	0.12 (0.12) {1}	3.91 (7.81)	0.24	nd
	*Micrococcus* *luteus* ATCC 10240	1000 (>1000) {>1}	1000 (>1000) {>1}	–	–	31.25 (250) {8}	62.5 (125) {2}	0.98 (1.95) {2}	62.5 (62.5)	0.98	nd
	*Bacillus* *subtilis* ATCC 6633	–	1000 (>1000) {>1}	–	–	1000 (>1000) {>1}	500 (500) {1}	0.03 (0.03) {1}	3.91 (3.91)	15.62	62.5
	*Bacillus* *cereus* ATCC 10876	–	–	–	–	500 (>1000) {>2}	125 (250) {2}	0.06 (0.12) {2}	7.81 (15,62)	31.25	nd
**Fungi**	*Candida* *albicans* ATCC 10231	1000 (>1000) {>1}	1000 (>1000) {>1}	–	1000 (>1000) {>1}	500 (>1000) {>2}	500 (1000) {2}	0.48 * (0.48) {1}	na	na	na
	*Candida* *albicans* ATCC 2091	–	–	–	–	500 (>1000) {>2}	500 (>1000) {>2}	0.24 * (0.24) {1}	na	na	na
	*Candida* *parapsilosis* ATCC 22019	–	–	–	–	1000 (>1000) {>1}	1000 (>1000) {>1}	0.24 * (0.48) {2}	na	na	na
	*Candida* *glabrata* ATCC 90030	–	–	1000 (>1000) {>1}	1000 (>1000) {>1}	250 (>1000) {>4}	250 (1000) {4}	0.24 * (0.48) {2}	na	na	na
	*Candida* *krusei* ATCC 14243	1000 (>1000) {>1}	–	1000 (>1000) {>1}	–	500 (>1000) {>2}	500 (>1000) {>2}	0.24 * (0.24) {1}	na	na	na

The standard antimicrobial drugs used as positive controls: ciprofloxacin (CIP), nitrofurantoin (NIT), cefuroxime (CFX), ampicillin (APC) for bacteria and nystatin (NY*) for fungi; “–” no activity; nd, not determined; na, not applicable.

**Table 3:** The activity data of the studied compounds **5**, **15**–**21** expressed as MIC (MBC or MFC) [µg/mL] and {MBC/MIC or MFC/MIC} values against the reference strains of bacteria and fungi.

Species		MIC (MBC or MFC) [µg/mL] and {MBC/MIC or MFC/MIC} Values of the Studied Compounds and Positive Controls										
		5	15	17	18	19	20	21	CIP/NY*	NIT	CFX	APC
**Gram-positive bacteria**	*Staphylococcus* *aureus* ATCC 25923	–	250 (>1000) {>4}	31.25 (31.25) {1}	–	–	1.95 (3.91) {2}	7.81 (31.25) {4}	0.48 (0.48) {1}	15.62 (15.62)	0.49	nd
	*Staphylococcus* *aureus* ATCC 29213	–	250 (>1000) {>4}	15.62 (15.62) {1}	–	–	3.91 (3.91) {1}	15.62 (15.62) {1}	0.48 (0.48) {1}	nd	nd	nd
	*Staphylococcus* *aureus* ATCC 43300	–	1000 (>1000) {>1}	15.62 (15.62) {1}	–	–	3.91 (7.81) {2}	15.62 (62.5) {4}	0.24 (0.24) {1}	7.81	nd	nd
	*Staphylococcus* *epidermidis* ATCC 12228	1000 (>1000) {>1}	31.25 (125) {4}	15.62 (62.5) {4}	500 (>1000) {>2}	–	1.95 (7.81) {4}	7.81 (31.25) {4}	0.12 (0.12) {1}	3.91 (7.81)	0.24	nd
	*Micrococcus* *luteus* ATCC 10240	1000 (>1000) {>1}	31.25 (125) {4}	15.62 (15.62) {1}	31.25 (250) {8}	–	1.95 (7.81) {4}	3.91 (15.62) {4}	0.98 (1.95) {2}	62.5 (62.5)	0.98	nd
	*Bacillus* *subtilis* ATCC 6633	1000 (>1000) {>1}	125 (500) {4}	7.81 (15.62) {1}	62.5 (>1000) {>16}	–	7.81 (31.25) {4}	7.81 (31.25) {4}	0.03 (0.03) {1}	3.91 (3.91)	15.62	62.5
	*Bacillus* *cereus* ATCC 10876	–	125 (250) {2}	15.62 (15.62) {1}	–	–	7.81 (31.25) {4}	15.62 (62.5) {4}	0.06 (0.12) {2}	7.81 (15.62)	31.25	nd
**Gram-negative bacteria**	*Bordetella* *bronchiseptica* ATCC 4617	–	–	–	–	–	500 (>1000) {>2}	–	0.98 (0.98) {1}	125	nd	nd
	*Klebsiella* *pneumoniae* ATCC 13883	–	–	–	–	–	500 (>1000) {>2}	–	0.12 (0.24) {2}	15.62	nd	nd
	*Proteus* *mirabilis* ATCC 12453	–	–	–	–	–	500 (1000) {2}	–	0.03 (0.03) {1)	62.5	nd	nd
	*Escherichia* *coli* ATCC 25922	–	–	–	–	–	1000 (>1000) {>1}	–	0.06 (0.06) {1}	7.81	nd	nd
	*Salmonella* *typhimurium* ATCC 14028	–	–	–	–	–	1000 (>1000) {>1}	–	0.004 (0.008) {2}	31.25	nd	nd
	*Pseudomonas* *aeruginosa* ATCC 9027	–	–	–	–	–	500 (>1000) {>2}	–	0.48 (0.98) {2}	nd	nd	nd
**Fungi**	*Candida* *albicans* ATCC 10231	–	–	–	–	250 (>1000) {>4}	250 (>1000) {>4}	250 (>1000) {>4}	0.48 * (0.48) {1}	na	na	na
	*Candida* *albicans* ATCC 2091	–	–	–	–	500 (>1000) {>2}	500 (>1000) {>2}	500 (>1000) {>2}	0.24 * (0.24) {1}	na	na	na
	*Candida* *parapsilosis* ATCC 22019	–	–	–	–	125 (>1000) {>8}	125 (>1000) {>8}	250 (>1000) {>4}	0.24 * (0.48) {2}	na	na	na
	*Candida* *glabrata* ATCC 90030	–	–	–	–	500 (>1000) {>2}	125 (>1000) {>8}	125 (>1000) {>8}	0.24 * (0.48) {2}	na	na	na
	*Candida* *krusei* ATCC 14243	–	–	–	–	250 (>1000) {>4}	500 (>1000) {>2}	500 (>1000) {>2}	0.24 * (0.24) {1}	na	na	na

The standard antimicrobial drugs used as positive controls: ciprofloxacin (CIP), nitrofurantoin (NIT), cefuroxime (CFX), ampicillin (APC) for bacteria and nystatin (NY*) for fungi; “–” no activity; nd, not determined; na, not applicable.

**Table 4:** The activity data of the studied compounds **6**, **22**–**28** expressed as MIC (MBC or MFC) [µg/mL] and {MBC/MIC or MFC/MIC} values against the reference strains of bacteria and fungi.

Species		MIC (MBC or MFC) [µg/mL] and {MBC/MIC or MFC/MIC} Values of the Studied Compounds and Positive Controls										
		6	23	24	25	26	27	28	CIP/ NY*	NIT	CFX	APC
**Gram-positive bacteria**	*Staphylococcus* *aureus* ATCC 25923	–	1000 (>1000) {>1}	–	–	–	62.5 (250) {4}	–	0.48 (0.48) {1}	15.62 (15.62)	0.49	nd
	*Staphylococcus* *aureus* ATCC 29213	–	500 (>1000) {>2}	1000 (>2000)	–	–	62.5 (125) {2}	–	0.48 (0.48) {1}	nd	nd	nd
	*Staphylococcus* *aureus* ATCC 43300	–	500 (>1000) {>2}	–	–	1000 (>1000) {>1}	62.5 (250) {4}	–	0.24 (0.24) {1}	7.81	nd	nd
	*Staphylococcus* *epidermidis* ATCC 12228	1000 (>1000) {>1}	1000 (>1000) {>1}	–	1000 (>1000) {>1}	1000 (>1000) {>1}	31.25 (31.25) {1}	–	0.12 (0.12) {1}	3.91 (7.81)	0.24	nd
	*Micrococcus* *luteus* ATCC 10240	500 (>1000) {>2}	500 (>1000) {>2}	500 (>1000) {>2}	31.25 (62.5) {2}	500 (>1000) {>2}	125 (250) {2}	–	0.98 (1.95) {2}	62.5 (62.5)	0.98	nd
	*Bacillus* *subtilis* ATCC 6633	1000 (>1000) {>1}	1000 (>1000) {>1}	500 (>1000) {>2}	–	500 (>1000) {>2}	125 (500) {4}	–	0.03 (0.03) {1}	3.91 (3.91)	15.62	62.5
	*Bacillus* *cereus* ATCC 10876	–	1000 (>1000) {>1}	–	–	–	62.5 (250) {4}	–	0.06 (0.12) {2}	7.81 (15,62)	31.25	nd
**Gram-negative bacteria**	*Bordetella* *bronchiseptica* ATCC 4617	–	–	–	–	–	500 (1000) {2}	–	0.98 (0.98) {1}	125	nd	nd
	*Klebsiella* *Pneumoniae* ATCC 13883	–	–	–	–	–	500 (>1000) {>2}	–	0.12 (0.24) {2}	15.62	nd	nd
	*Proteus* *mirabilis* ATCC 12453	–	–	–	–	–	500 (1000) {2}	–	0.03 (0.03) {1)	62.5	nd	nd
	*Escherichia* *coli* ATCC 25922	–	–	–	–	–	500 (500) {1}	–	0.06 (0.06) {1}	7.81	nd	nd
	*Salmonella* *typhimurium* ATCC 14028	–	–	–	–	–	500 (1000) {2}	–	0.004 (0.008) {2}	31.25	nd	nd
	*Pseudomonas* *aeruginosa* ATCC 9027	–	–	–	–	–	1000 (>1000) {>1}	–	0.48 (0.98) {2}	nd	nd	nd
**Fungi**	*Candida* *albicans* ATCC 10231	–	–	500 (>1000) {>2}	–	500 (>1000) {>2}	15.62 (31.25) {2}	500 (>1000) {>2}	0.48 * (0.48) {1}	na	na	na
	*Candida* *albicans* ATCC 2091	–	–	1000 (>1000) {>1}	–	1000 (>1000) {>1}	62.5 (62.5) {1}	1000 (>1000) {>1}	0.24 * (0.24) {1}	na	na	na
	*Candida* *parapsilosis* ATCC 22019	250 (>1000) {>8}	1000 (>1000) {>1}	1000 (>1000) {>1}	250 (>1000) {>8}	250 (>1000) {>4}	31.25 (62.5) {2}	500 (>1000) {>2}	0.24 * (0.48) {2}	na	na	na
	*Candida* *glabrata* ATCC 90030	–	–	250 (>1000) {>4}	–	1000 (>1000) {>1}	31.25 (62.5) {2}	1000 (>1000) {>1}	0.24 * (0.48) {2}	na	na	na
	*Candida* *krusei* ATCC 14243	–	–	–	–	500 (>1000) {>2}	62.5 (62.5) {1}	1000 (>1000) {>1}	0.24 * (0.24) {1}	na	na	na

The standard antimicrobial drugs used as positive controls: ciprofloxacin (CIP), nitrofurantoin (NIT), cefuroxime (CFX), ampicillin (APC) for bacteria and nystatin (NY*) for fungi; “–” no activity; nd, not determined; na, not applicable.

## Data Availability

Data are contained within this article and Supplementary Materials.

## Supplementary Materials

The following supporting information can be downloaded at https://www.mdpi.com/article/10.3390/molecules29163814/s1: Mechanochemical synthesis of acylhydrazones—Figure S1; X-ray crystallography—Table S1–S6, Figure S2–S5; Microbiology—in vitro antimicrobial activity assays; Cytotoxicity—cell proliferation—List of tables: Tables S7–S14, List of figures: Figures S6–S37; Examples of IR, ^1^H NMR and ^13^C NMR of synthesized hydrazides and acylhydrazones—List of figures: Figures S38–S55. References [46,47,48] are cited in the supplementary materials.
