# Supplementary material for: Synthesis, Structural Properties and Biological Activities of Novel Hydrazones of 2-, 3-, 4-Iodobenzoic Acid
Source: Molecules. 2024 Aug 11;29(16):3814. doi: 10.3390/molecules29163814 (PMC11356900; doi:10.3390/molecules29163814)
Supplement: Supplementary file 1 [file molecules-29-03814-s001.zip › molecules-3057036-supplementary.pdf]

Article

Synthesis, Structural Properties and Biological Activities of Novel Hydrazones of 2-, 3-, 4-Iodobenzoic Acid

Izabela Czyżewska [1], Liliana Mazur [2], Anna Biernasiuk [3], Anna Hordyjewska [4] and Łukasz Popiołek [1,*]

[1] Chair and Department of Organic Chemistry, Faculty of Pharmacy, Medical University of Lublin, 4A Chodźki Street, 20-093 Lublin, Poland; 50460@student.umlub.pl
[2] Institute of Chemical Sciences, Faculty of Chemistry, Maria Curie–Skłodowska University, Maria Curie–Skłodowska Square 2, 20-031 Lublin, Poland; liliana.mazur@mail.umcs.pl
[3] Chair and Department of Pharmaceutical Microbiology, Faculty of Pharmacy, Medical University of Lublin, 1 Chodźki Street, 20-093 Lublin, Poland; anna.biernasiuk@umlub.pl
[4] Chair and Department of Medicinal Chemistry, Faculty of Medical Sciences, Medical University of Lublin, 4A Chodźki Street, 20-093 Lublin, Poland; anna.hordyjewska@umlub.pl
* Correspondence: lukasz.popiolek@umlub.pl; Tel.: +48-81-448-72-43

Supplementary Materials

1. Synthesis, structural studies and spectral characteristics of the studied acylhydrazones

1.1. Mechanochemical synthesis of acylhydrazones

(I)

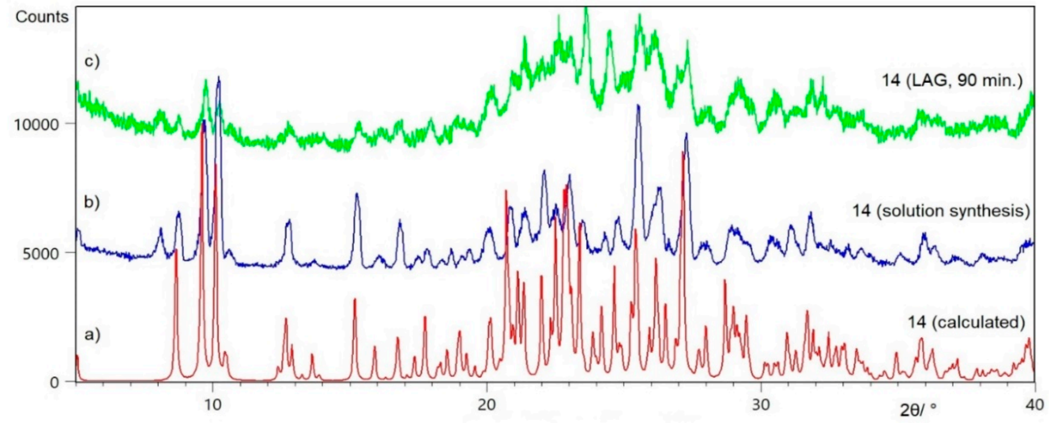

(II)

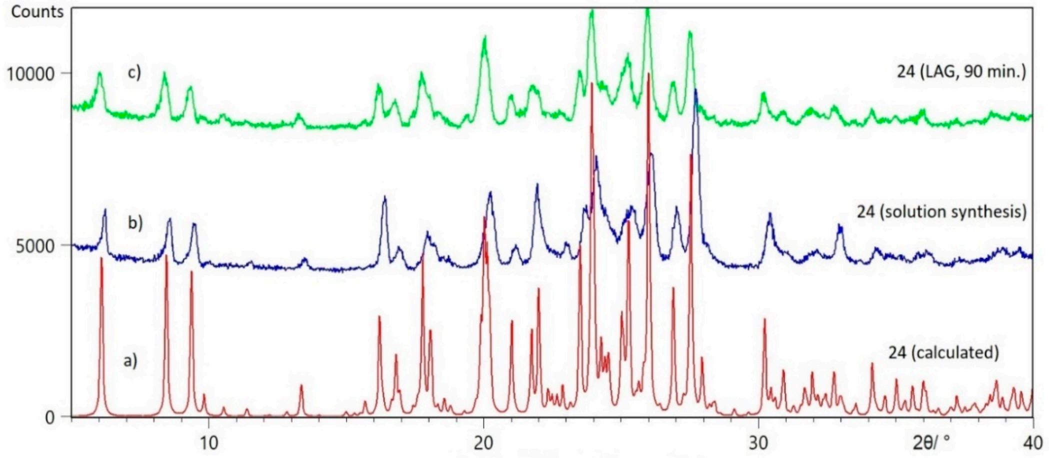

(III)

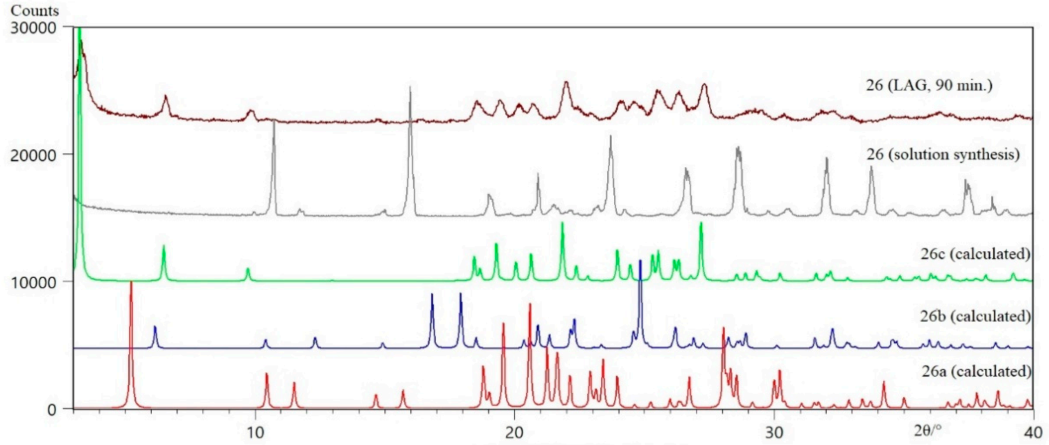

Figure S1. PXRD patterns of compound: (**I**) **14** – simulated from the SCXRD data, experimental after the synthesis from solution and after LAG for 90 min. using ethanol; (**II**) **24** – simulated from SCXRD data, experimental after the synthesis from solution, experimental after LAG for 90 min. using ethanol; (**III**) **26** – simulated from the SCXRD data (crystal structures **26a**, **26b**, **26c**), experimental after the solution synthesis and after LAG for 90 min. using ethanol.

1.2. *X-ray crystallography*

Table S1. Crystal data and structure refinement details for the studied acylhydrazones.

**Crystal structure**	**9**	**12**	**13**	**13·ACN**	**14**
Chemical formula	$C_{14}H_9N_2O_2ICl_2$	$C_{14}H_9N_2O_2I_2Cl$	$C_{14}H_9$ $N_2O_2IBr_2$	$C_{16}H_{12}N_3O_2IBr_2$ $\cdot CH_3CN$	$C_{14}H_9N_2O_2I_3$
Formula weight	435.03	526.48	523.95	565.01	617.93
T / K	293	293	293	293	293
Crystal system	monoclinic	monoclinic	monoclinic	orthorhombic	monoclinic
Space group	$P2_1/c$	$P2_1/n$	$P2_1/c$	*Pbca*	$P2_1/c$
a / Å	18.7006(4)	18.9192(4)	18.9820(7)	7.2352(1)	19.4686(3)
b/ Å	9.5210(2)	9.5250(1)	9.5183(3)	22.9495(2)	9.6874(1)
c / Å	20.2936(4)	20.7505(4)	20.4590(6)	23.2354(3)	20.4776(3)
α / °	90	90	90	90	90
β / °	115.229(2)	116.442(3)	115.753(4)	90	115.951(2)
γ/ °	90	90	90	90	90
V / Å^3	3268.6(1)	3348.2(1)	3329.3(2)	3858.1(1)	3472.7(1)
Z/ Z'	8/ 2	8/ 2	8/ 2	8/ 1	8/ 2
d_{calc} / g·cm^{-3}	1.768	2.089	2.091	1.945	2.364
Θ range / °	4.39–68.57	4.24–68.71	4.35–68.54	3.81–68.48	4.34–68.51
μ / mm^{-1}	18.45	31.03	20.79	18.01	42.462
Crystal size/ mm^3	0.35x0.05x0.03	0.3x0.06x0.03	0.27x0.04x0.02	0.42×0.06×0.04	0.25x0.04x0.03
Crystal color & form	yellow needle	yellow needle	yellow needle	colorless needle	yellow needle
R_{int}	0.052	0.074	0.050	0.050	0.079
Refl. coll./unique	31405/5944	33639/6162	33319/6110	70589/3550	36916; 6380
Refl. with I>2σ(I)	4483	4929	4538	3386	5419
Param./ restraints	387/0	395/0	387/0	226/0	379/ 0
R_1; wR_2 [I>2σ(I)]	0.060; 0.160	0.060; 0.160	0.053; 0.128	0.048; 0.122	0.055; 0.151
R_1; wR_2 [all data]	0.073; 0.170	0.072; 0.171	0.070; 0.138	0.049; 0.123	0.062; 0.157
GooF on F^2	1.11	1.03	1.02	1.07	1.09
$\varrho_{res}^{min/max}$ / e·Å^{-3}	-1.57/ 1.20	-1.57/ 1.53	-1.13/ 1.33	-1.73/ 2.65	-1.91/ 2.30

**Crystal structure**	**20**	**24**	**26a**	**26b**	**26c**
Chemical formula	$C_{14}H_9N_2O_2I_2Cl$ $\cdot H_2O$	$C_{14}H_9N_2O_2ICl_2$	$C_{14}H_{11}N_2O_3I$	$C_{14}H_{11}N_2O_3I$	$C_{14}H_{11}N_2O_3I$
Formula weight	544.50	435.03	382.15	382.15	382.15
T / K	293	293	293	293	293
Crystal system	monoclinic	monoclinic	orthorhombic	monoclinic	monoclinic
Space group	$P2_1/c$	$C2/c$	$Pca2_1$	$P2_1$	$P2_1$
a / Å	15.3068(3)	21.3618(3)	33.8455(4)	4.5303(1)	4.8826(1)
b/ Å	7.6017(1)	9.9728(2)	4.7071(1)	10.5368(2)	5.0674(1)
c / Å	15.2558(3)	29.6378(4)	8.6261(1)	14.3691(3)	27.2710(4)
α / °	90	90	90	90	90
β / °	110.106(2)	101.705(1)	90	90.737(2)	90.047(1)
γ/ °	90	90	90	90	90
V / Å^3	1666.95(5)	6182.7(2)	1374.26(4)	685.85(2)	674.74(2)
Z/ Z'	4/ 1	16/ 2	4/ 1	2/ 1	2/ 1
d_{calc} / g·cm^{-3}	2.170	1.869	1.847	1.850	1.881
Θ range / °	5.88–68.03	4.23–68.52	5.23–68.44	5.21–67.91	4.87–68.07
μ / mm^{-1}	31.23	19.51	18.40	18.44	18.74
Crystal size/ mm^3	0.32x0.14x0.08	0.35×0.03×0.02	0.42×0.03×0.02	0.29x0.15x0.06	0.33x0.14x0.03
Crystal color & form	colorless block	yellow needle	colorless needle	yellow plate	yellow plate
R_{int}	0.044	0.047	0.065	0.030	0.043
Refl. coll./unique	9334/3002	33752/5643	16738/2394	6043/2400	5558/2030
Refl. with I>2σ(I)	2821	4512	2271	2358	2000
Param./restraints	202/0	395/0	193/1	189/1	189/1
R_1; wR_2 [I>2σ(I)]	0.043; 0.116	0.036; 0.089	0.029; 0.071	0.031; 0.082	0.038; 0.100
R_1; wR_2 [all data]	0.045; 0.118	0.046; 0.095	0.032; 0.073	0.031; 0.082	0.038; 0.101
GooF on F^2	1.04	1.05	1.07	1.08	1.05
$\varrho_{res}^{min/max}$ / e·Å^{-3}	-2.25/ 1.40	-0.68/ 1.00	-0.39/ 0.34	-0.55/ 0.42	-0.68/ 1.35

9

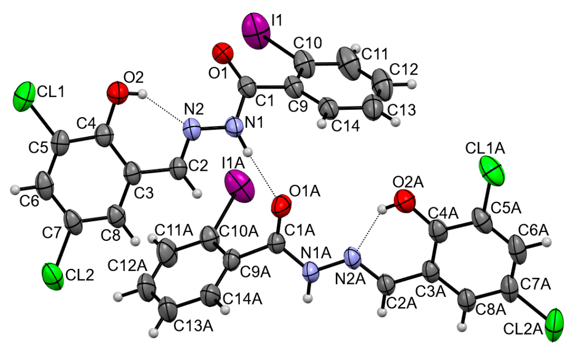

12

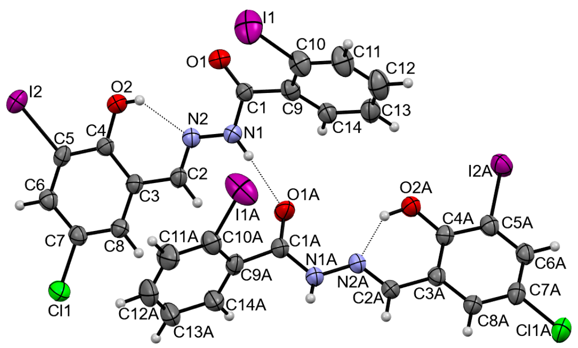

14

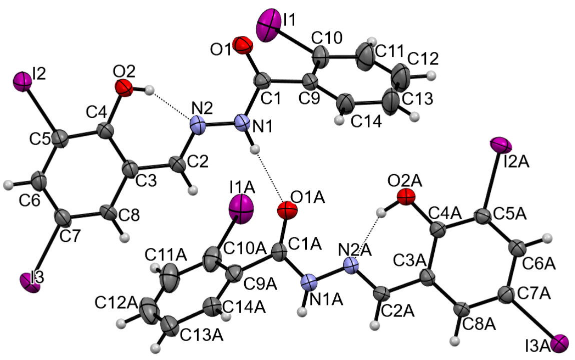

24

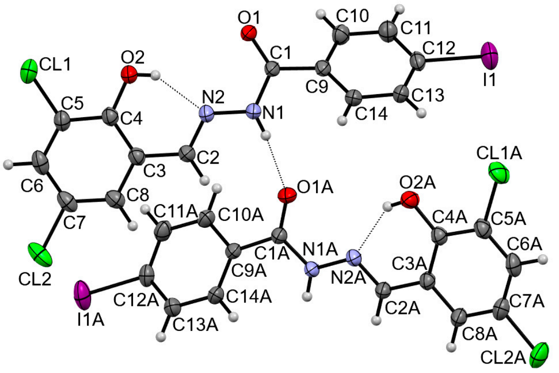

26b

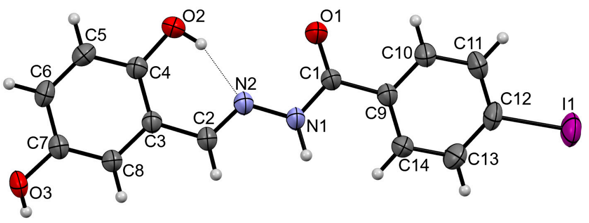

26c

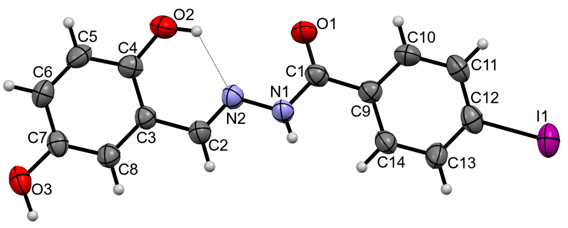

Figure S2. Perspective view of the molecules constituting the asymmetric part in crystals of **9**, **12**, **14**, **24**, **26b** and **26c** with labelling of atoms and estimation of their thermal motion parameters as ADPs (50% probability level). Dashed lines indicate hydrogen bonds.

Table S2. Selected bond distances (Å) in crystals of studied acylhydrazones.

Struct. / Bond	9		12		13		13·ACN	14	
	A	B	A	B	A	B		A	B
C1–O1	1.230(7)	1.211(7)	1.219(8)	1.217(9)	1.218(8)	1.215(9)	1.222(7)	1.213(10)	1.226(10)
C1–N1	1.342(8)	1.341(7)	1.356(9)	1.33(1)	1.344(9)	1.332(10)	1.342(8)	1.341(10)	1.336(10)
N1–N2	1.374(6)	1.377(6)	1.371(8)	1.378(8)	1.385(7)	1.378(8)	1.370(6)	1.373(9)	1.377(9)
N2–C2	1.284(7)	1.283(7)	1.27(1)	1.264(9)	1.288(9)	1.272(9)	1.276(7)	1.268(10)	1.267(10)
C2–C3	1.465(8)	1.453(7)	1.45(1)	1.465(9)	1.454(9)	1.458(9)	1.454(8)	1.451(11)	1.454(11)
C4–O2	1.342(7)	1.349(8)	1.330(9)	1.342(9)	1.342(9)	1.340(8)	1.344(7)	1.341(10)	1.345(9)
C10–I1	2.101 (7)	2.095(6)	2.116(9)	2.099(9)	2.096(8)	2.109(9)	2.102(5)	2.093(9)	2.124(12)
C5–Cl1	1.742(7)	1.721(7)	-	-	-	-	-	-	-
C5–Br1	-	-	-	-	1.879(8)	1.889(7)	1.890(6)	-	-
C5–I2	-	-	2.102(7)	2.089(8)	-	-	-	2.106(9)	2.110(7)
C7–Cl	1.740(7)	1.741(7)	1.748(8)	1.741(8)	-	-	-	-	-
C7–Br	-	-	-	-	1.898(8)	1.892(7)	1.889(6)	-	-
C7–I3	-	-	-	-	-	-	-	2.098(8)	2.100(8)

Structure / Bond	20	24		26a	26b	26c
		A	B			
C1–O1	1.215(6)	1.230(4)	1.223(4)	1.244(7)	1.230(7)	1.227(8)
C1–N1	1.364(6)	1.352(5)	1.351(4)	1.330(7)	1.361(7)	1.355(7)
N1–N2	1.370(6)	1.375(4)	1.379(4)	1.392(6)	1.363(7)	1.376(9)
N2–C2	1.269(7)	1.282(4)	1.273(4)	1.276(7)	1.285(7)	1.277(7)
C2–C3	1.455(7)	1.452(5)	1.448(4)	1.452(7)	1.447(8)	1.485(10)
C4–O2	1.359(6)	1.344(4)	1.341(4)	1.366(8)	1.370(8)	1.361(8)
C11–I1	2.097(6)	-	-	-	-	-
C12–I1	-	2.101(3)	2.095(3)	2.102(4)	2.083(6)	2.103(7)
C5–Cl1	-	1.736(4)	1.730(4)	-	-	-
C5–I2	2.095(5)	-	-	-	-	-
C7–Cl	1.729(5)	1.739(4)	1.737(4)	-	-	-

Table S3. Selected bond angles (°) in crystals of studied acylhydrazones.

Structure / Angle	9		12		13		13·ACN	14	
	A	B	A	B	A	B		A	B
O1–C1–N1	122.2(5)	122.2(5)	121.5(7)	122.4(7)	122.7(6)	122.3(7)	121.3(5)	122.0(7)	122.7(7)
O1–C1–C9	123.4(5)	123.0(5)	124.0(7)	122.1(7)	122.5(6)	123.4(7)	121.1(5)	123.2(7)	123.3(8)
N1–C1–C9	114.4(5)	114.8(5)	114.5(6)	115.5(7)	114.7(6)	114.2(7)	117.6(5)	114.8(7)	114.0(7)
C1–N1–N2	117.9(5)	116.9(5)	117.6(6)	118.0(7)	116.2(6)	118.8(7)	121.3(5)	118.9(7)	117.4(6)
C2–N2–N1	117.1(5)	117.7(5)	117.6(6)	117.9(6)	116.6(6)	118.2(6)	116.5(5)	117.9(7)	118.8(6)
N2–C2–C3	119.0(5)	119.6(5)	120.0(6)	119.9(7)	119.8(7)	119.2(7)	120.8(5)	120.7(8)	120.6(7)
C2–C3–C4	121.3(5)	121.8(5)	121.4(7)	121.8(6)	121.1(7)	121.3(6)	121.3(5)	121.6(7)	120.7(7)
C1–C9–C10	122.1(6)	120.8(5)	122.7(7)	122.0(7)	121.6(7)	121.4(7)	123.9(5)	120.7(7)	121.7(9)

Structure / Angle	20	24		26a	26b	26c
		A	B			
O1–C1–N1	120.2(5)	122.0(3)	121.8(3)	121.5(5)	120.3(5)	122.3(6)
O1–C1–C9	122.6(5)	122.5(3)	122.5(3)	122.4(5)	121.4(5)	122.6(6)
N1–C1–C9	117.2(4)	115.5(3)	115.7(3)	116.1(5)	118.2(5)	115.0(5)
C1–N1–N2	117.0(4)	117.8(3)	118.2(3)	119.4(5)	115.7(5)	116.9(5)
C2–N2–N1	120.4(4)	118.4(3)	117.6(3)	116.7(5)	119.7(5)	116.8(6)
N2–C2–C3	118.8(4)	119.0(3)	120.3(3)	120.0(5)	119.3(5)	117.8(6)
C2–C3–C4	122.0(4)	121.2(3)	121.5(3)	121.9(5)	122.1(5)	122.1(5)
C1–C9–C10	116.5(5)	119.0(3)	118.4(3)	118.9(5)	117.4(5)	118.4(5)

Table S4. Selected torsion angles (°) in crystals of studied acylhydrazones.

Structure / Angle	9		12		13		13·ACN	14	
	A	B	A	B	A	B		A	B
C9–C1–N1–N2	179.4(5)	-179.4(5)	-178.0(6)	179.2(7)	-179.4(6)	178.2(5)	0.7(8)	-179.8(7)	-179.1(7)
O1–C1–N1–N2	-0.2(9)	-0.2(9)	0.0(10)	0.6(12)	-2.5(10)	1.4(10)	-176.4(6)	1.0(13)	-0.8 (12)
C1–N1–N2–C2	173.6(5)	-176.9(5)	-174.7(6)	175.0(7)	-175.1(6)	173.8(6)	-176.9(5)	172.5(8)	-174.2(8)
N1–N2–C2–C3	176.6(5)	-179.5(5)	178.8(6)	-179.0(6)	179.6(6)	-176.4(5)	-177.7(5)	-178.4(7)	176.5(7)
C2–C3–C4–O2	-3.4(8)	-0.5(9)	3.1(10)	-0.5(11)	-0.5(11)	-3.5(9)	-2.9(8)	0.3(13)	4.5(12)
N1–C1–C9–C10	140.7(6)	-116.6(6)	-142.4(8)	116.9(9)	-117.9(8)	142.2(7)	83.0(7)	122.0(9)	-140.9(9)
O2–C4–C3–C2	-3.4(8)	-0.5(9)	3.1(10)	-0.5(11)	-0.5(11)	-3.5(9)	-2.9(8)	0.3(13)	4.5(12)
C4–C3–C2–N2	-3.0(8)	1.7(8)	0.6(11)	-0.4(11)	1.9(10)	-3.2(9)	2.6(8)	-0.9(13)	1.5(12)

Structure / Angle	20	24		26a	26b	26c
		A	B			
C9–C1–N1–N2	176.8(4)	179.9(3)	-175.3(3)	179.1(5)	-172.6(5)	176.7(6)
O1–C1–N1–N2	-3.5(7)	0.0(6)	5.8(5)	-1.1(9)	5.5(9)	-2.5(10)
C1–N1–N2–C2	-178.8(5)	169.6(3)	175.9(3)	165.6(5)	173.5(5)	167.2(7)
N1–N2–C2–C3	179.6(4)	174.8(3)	-178.6(3)	179.6(4)	-178.4(5)	177.3(6)
C2–C3–C4–O2	-0.1(7)	-3.8(5)	2.7(6)	2.2(8)	0.1(8)	0.6(10)
N1–C1–C9–C10	-178.3(5)	146.5(4)	-152.5(3)	-154.5(6)	-163.0(6)	-150.6(6)
O2–C4–C3–C2	-0.1(7)	-3.8(5)	2.7(6)	2.2(8)	0.1(8)	0.6(10)
C4–C3–C2–N2	0.6(7)	-5.1(5)	-0.3(6)	-7.4(8)	-6.1(8)	-6.2(11)

Table S5. The dihedral angles (°) between the best planes of phenyl rings in the studied crystals.

Structure	Molecule A	Molecule B
**9**	51.6	71.7
**12**	48.1	71.4
**13**	71.4	50.8
**13·ACN**	81.9	-
**14**	69.8	49.7
**20**	3.7	-
**24**	51.5	28.0
**26a**	4.9	-
**26b**	16.8	-
**26c**	10.3	-

Table S6. Geometries of proposed hydrogen bonds and selected short intermolecular contacts.

D–H···A	d(D–H)/Å	d(H···A)/Å	d(D···A)/Å	<DHA/°
**9**				
N1–H1n···O1A	0.86(6)	1.88(6)	2.741(6)	174(4)
N1A–H1nA···O1$^{(i)}$	0.86(6)	1.98(6)	2.799(6)	159(5)
O2–H2o···N2	0.78	1.89	2.582(7)	149
O2A–H20A···N2A	0.79	1.91	2.597(8)	146
C6A–H6A···Cl2$^{(ii)}$	0.93	2.83	3.750(9)	170
C13A–H13A···O2$^{(i)}$	0.93	2.63	3.253(9)	125
**12**				
N1–H1n···O1A	0.82(8)	1.94(8)	2.752(9)	170(8)
N1A–H1nA···O1$^{(i)}$	0.75(9)	2.10(9)	2.806(9)	158(9)
O2–H2o···N2	0.75(11)	1.98(11)	2.589(8)	137(11)
O2A–H2oA···N2A	0.88(10)	1.85(10)	2.592(8)	141(9)
C6A–H6A···Cl1$^{(iii)}$	0.93	2.86	3.761(8)	162
C13–H13···O2A	0.93	2.67	3.434(9)	141
C14–H14···O1A	0.93	2.66	3.136(10)	111
C13A–H13A···O2$^{(i)}$	0.93	2.63	3.253(11)	125
C2A–H2A···C9$^{(i)}$	0.93	2.73	3.656(9)	172
C8A–H8A···C13$^{(i)}$	0.93	2.87	3.784(9)	167
**13**				
N1–H1n···O1A	0.95	1.88	2.782(7)	158
N1A–H1nA···O1$^{(i)}$	0.77(6)	1.99(6)	2.759(8)	177(5)
O2–H2o···N2	0.83(8)	1.83(8)	2.601(9)	154(5)
O2A–H2oA···N2A	1.00	1.767	2.576(8)	136
C6–H6···Br2A$^{(iv)}$	0.93	2.919	3.822(10)	164
C13–H13···O2A	0.93	2.669	3.277(11)	124
C14A–H14A···O1A$^{(i)}$	0.93	2.711	3.161(11)	111
**13·ACN**				
N1–H1n···O1$^{(v)}$	0.77(6)	2.11(6)	2.843(6)	161(4)
O2–H2o···N2	0.84(9)	1.96(9)	2.602(7)	132(7)
C8–H8···N3	0.93	2.728	3.574(8)	151
C16–H16c···O1$^{(vi)}$	0.96	2.645	3.44(2)	141
C11–H11···O2$^{(vii)}$	0.93	2.686	3.306(9)	125
C12–H12···O2$^{(vii)}$	0.93	2.795	3.363(9)	120
C13–H13···O2$^{(viii)}$	0.93	2.599	3.467(9)	156
**14**				
N1–H1n···O1A	0.88	1.99	2.840(10)	172
N1A–H1nA···O1$^{(ix)}$	0.87	1.93	2.794(9)	174
O2–H2o···N2	0.80	1.88	2.611(7)	152
O2A–H2oA···N2A	0.90	1.76	2.582(9)	150
C13A–H13A···O2$^{(ix)}$	0.93	2.70	3.479(13)	141
C14A–H14A···O1A$^{(ix)}$	0.93	2.67	3.144(13)	112
C6–H6···I3A$^{(x)}$	0.93	3.04	3.925(11)	160
**20**				
N1–H1n···O1w	0.84	2.08	2.906(6)	169
O2–H2o···N2	0.88	1.79	2.563(6)	146
O1w–H1w···O2$^{(xi)}$	0.85	2.18	3.016(7)	166
O1w–H2w···O2$^{(xii)}$	0.85	2.27	3.071(7)	157
O1w–H2w···I2$^{(xii)}$	0.85	3.23	3.699(7)	118

C6–H6···I1(x)	0.93	3.30	4.176(7)	158
C8–H8···O1w(xiii)	0.93	2.67	3.375(8)	131
C14–H14···O1w	0.93	3.40	2.502(8)	163
**24**				
N1–H1n···O1A	0.81(5)	2.03(5)	2.821(4)	168(3)
N1A–H1nA···O1(i)	0.83(4)	2.11(4)	2.919(4)	164(3)
O2–H2o···N2	0.77(5)	1.89(5)	2.566(4)	146(4)
O2A–H2oA···N2A	0.76(5)	1.94(5)	2.591(4)	144(4)
C2A–H2A···O1(i)	0.93	2.54	3.320(4)	142
C6–H6···Cl1A(xiv)	0.93	2.92	3.674(6)	139
C6A–H6A···Cl1(xv)	0.93	2.90	3.820(6)	172
C10–H10···O1(xvi)	0.93	2.64	3.430(5)	143
C13–H13···O2A	0.93	2.51	3.095(5)	121
C13A–H13A···O2(i)	0.93	2.54	3.254(5)	134
**26a**				
N1–H1n···O1(ix)	0.77(8)	2.22(8)	2.903(7)	149(6)
O3–H3o···O1(xvii)	0.74(8)	2.10(8)	2.820(8)	166(7)
O2–H2o···N2	0.89(9)	1.83(9)	2.620(8)	147(7)
C10–H10···O3(xviii)	0.93	2.74	3.629(8)	162
C2–H2···O2(xvii)	0.93	2.76	3.658(7)	163
C10–H10···I1(xix)	0.93	3.18	3.866(8)	132
C11–H11···I1(xx)	0.93	3.32	3.929(8)	125
C13–H13···I1(xxi)	0.93	3.27	4.019(8)	139
**26b**				
N1–H1n···O2(xxii)	1.00(7)	2.12(7)	3.085(6)	160(6)
O2–H2o···N2	0.81(11)	1.84(12)	2.599(7)	155(11)
O3–H3o···O1(xxiii)	0.81	1.91	2.705(6)	168
C8–H8···O1(xxiii)	0.93	2.74	3.304(8)	120
C8–H8···O1(xxii)	0.93	2.74	3.332(8)	122
C2–H2···O2(xxiv)	0.93	2.74	3.327(7)	122
**26c**				
N1–H1n···O1(xxv)	0.798(8)	2.193(8)	2.927(9)	153(8)
O2–H2o···N2	0.87(13)	2.01(13)	2.611(8)	125(8)
O3–H3o···O3(xxiv)	1.03	1.82	2.774(7)	153
C6–H6···O3(xxii)	0.93	2.65	3.424(10)	141
C13–H13···O1(xxvi)	0.93	2.56	3.341(10)	142

Symmetry codes: (i) *x*, *y*-1, *z*; (ii) *x*+1, -*y*+1/2, *z*+1/2; (iii) *x*+1/2, -*y*+1/2, *z*-1/2; (iv) *x*-1, -*y*+1/2, *z*-1/2; (v) -*x*, -*y*+1, -*z*+1; (vi) -*x*+1/2, -*y*+1, *z*+1/2; (vii) *x*+1/2, -*y*+3/2, -*z*+1; (viii) *x*-1/2, -*y*+3/2, -*z*+1; (ix) *x*, *y*+1, z; (x) *x*-1, -*y*+3/2, *z*-1/2; (xi) -*x*+1, -*y*+1, -*z*+1; (xii) *x*, -*y*+3/2, *z*+1/2; (xiii) -*x*+1, *y*+1/2, -*z*+3/2; (xiv) *x*+1/2, -*y*+3/2, *z*+1/2; (xv) *x*-1/2, -*y*+3/2, *z*-1/2; (xvi) -*x*+1, *y*, -*z*+3/2; (xvii) -*x*+1/2, *y*+1, *z*-1/2; (xviii) -*x*+1/2, *y*-1, *z*+1/2; (xix) -*x*, -*y*, *z*+1/2; (xx) -*x*, -*y*, *z*+1/2; (xxi) -*x*, -*y*+1, *z*-1/2; (xxii) -*x*+1, *y*-1/2, -*z*+1; (xxiii) -*x*, *y*-1/2, -*z*+1; (xxiv) -*x*, *y*+1/2, -*z*+1; (xxv) *x*-1, *y*, *z*; (xvii) *x*-1, *y*+1, *z*

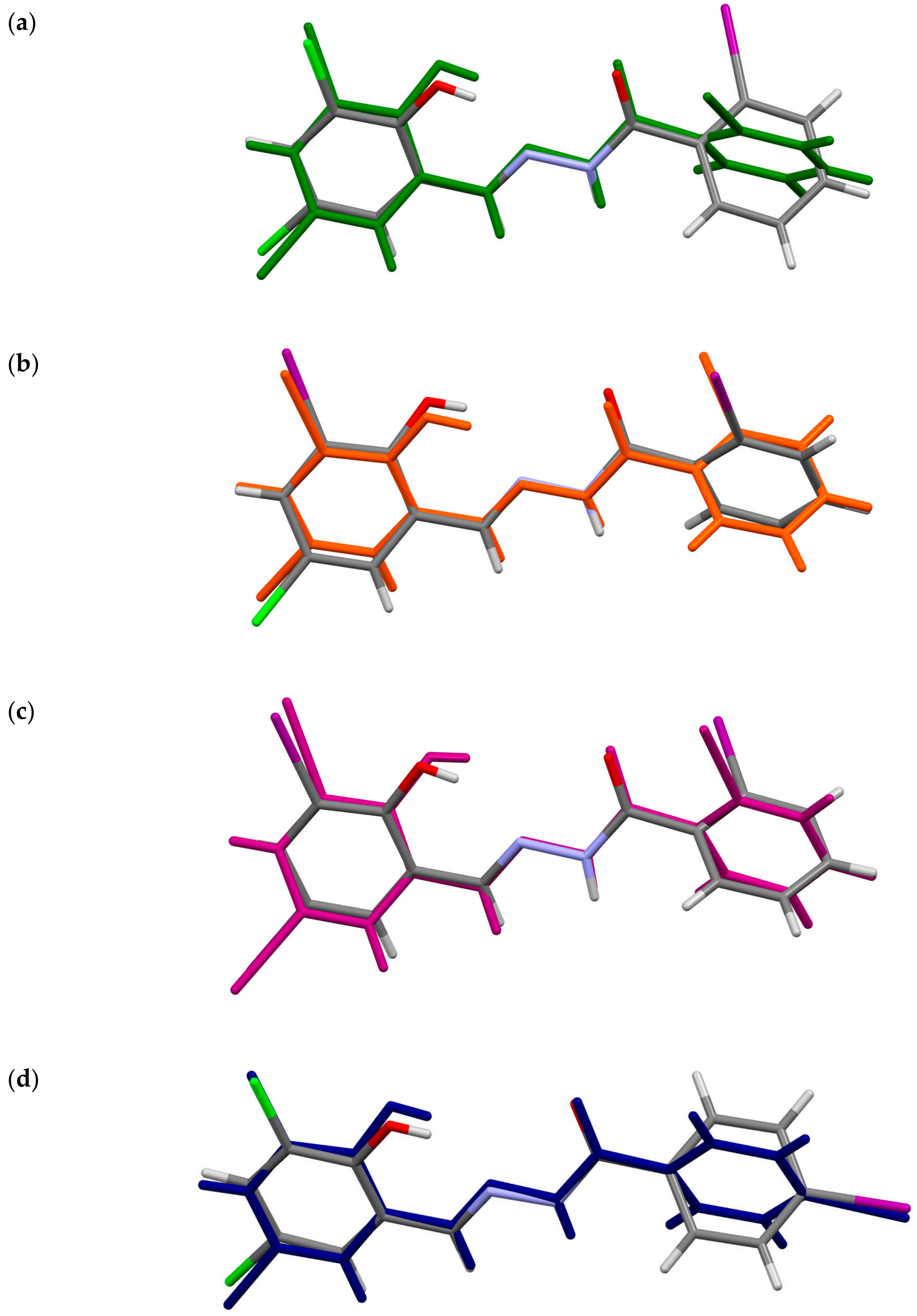

Figure S3. The overlay of two conformers present in the asymmetric part of the unit cell in crystals: (**a**) **9** (**9-A**: grey line; **9-B**: green line), (**b**) **12** (**12-A**: grey line; **12-B**: orange line), (**c**) **14** (**14-A**: grey line; **14-B**: violet line), (**d**) **24** (**24-A**: grey line; **24-B**: blue line).

(**a**) (**b**)

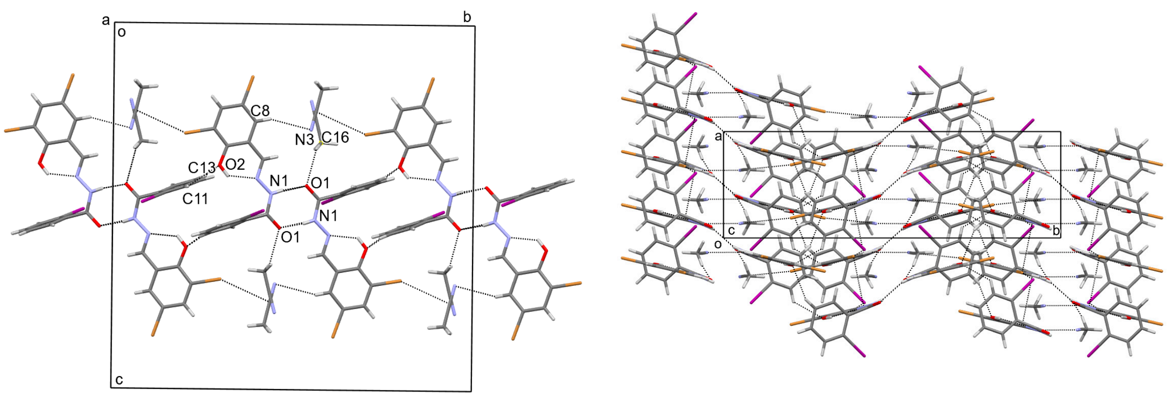

Figure S4. Part of the crystal structure of **13·ACN** showing: (**a**) centrosymmetric dimers stabilized by strong N1–H1n···O1 (-*x*, -*y*+1, -*z*+1) hydrogen bonds being parts of supramolecular chains *via* weak C–H···O/N interactions; (**b**) crystal packing viewed along the *c* axis. Dashed lines indicate hydrogen bonds.

(**a**) (**b**)

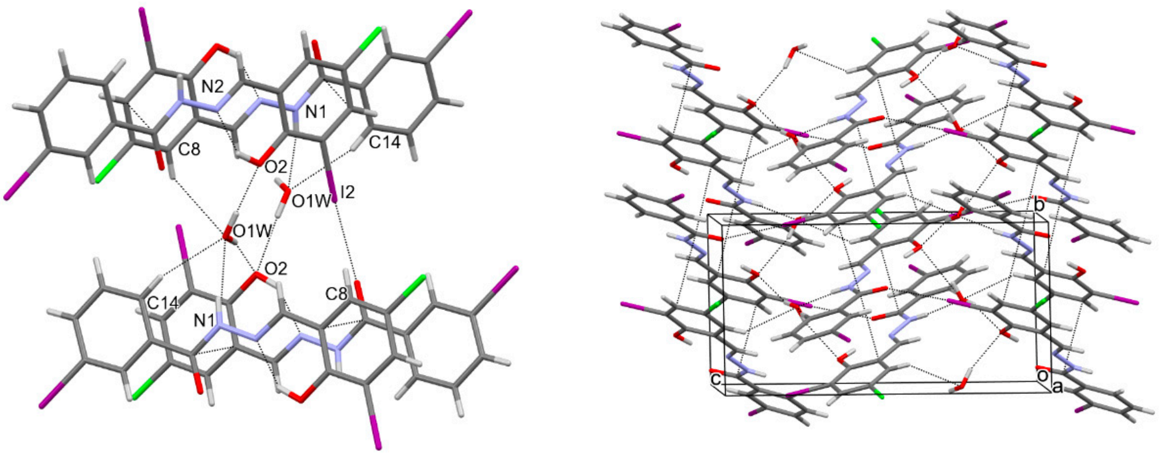

Figure S5. Part of the crystal structure of **20** showing: (**a**) intermolecular interactions involving water molecules; (**b**) crystal packing viewed along the *a* axis. Dashed lines indicate hydrogen bonds.

2. Microbiology – *in vitro* antimicrobial activity assays

The studied compounds **4–28** were tested under the *in vitro* conditions for antibacterial and antifungal activities using the broth microdilution method according to the European Committee on Antimicrobial Susceptibility Testing (EUCAST) [1] and Clinical and Laboratory Standards Institute guidelines [2] against a panel of reference and clinical or saprophytic strains of microorganisms, including the Gram-positive bacteria: *Staphylococcus aureus* ATCC 43300 (Methicillin Resistant *S. aureus* – MRSA), *Staphylococcus aureus* ATCC 29213 (Methicillin Susceptible *S. aureus* – MSSA), *Staphylococcus epidermidis* ATCC 12228, *Micrococcus luteus* ATCC 10240, *Bacillus subtilis* ATCC 6633 and *Bacillus cereus* ATCC 10876), Gram-negative bacteria: *Bordetella bronchiseptica* ATCC 4617, *Escherichia coli* ATCC 25922, *Klebsiella pneumoniae* ATCC 13883, *Proteus mirabilis* ATCC 12453, *Salmonella typhimurium* ATCC 14028 and *Pseudomonas aeruginosa* ATCC 9027, and fungi belonging to yeasts: *Candida albicans* ATCC 10231, *Candida albicans* ATCC 2091, *Candida parapsilosis* ATCC 22019, *Candida glabrata* ATCC 90030 and *Candida krusei* ATCC 14243. The microorganisms belonging to ATCC originated from American Type Culture Collection, routinely used for the evaluation of antimicrobials. All the used microbial cultures were first subcultured on nutrient agar or Sabouraud agar at 35°C for 18-24 h or 30°C for 24-48 h for bacteria and fungi, respectively.

The surface of Mueller-Hinton agar (for bacteria) and RPMI 1640 with MOPS (for fungi) were inoculated with the suspensions of bacterial or fungal species. Microbial suspensions were prepared in sterile saline with an optical density of McFarland standard scale 0.5. The samples containing the compounds were dissolved in dimethyl sulfoxide (DMSO). Furthermore, the bacterial and fungal suspensions put onto the Petri dishes with solid media containing 2 mg/mL of the tested compounds followed were incubated at 37°C for 24h and 30°C for 48h for bacteria and fungi, respectively. The inhibition of microbial growth was estimated comparing with a control culture prepared without any tested samples. Ciprofloxacin, nitrofurantoin, cefuroxime, ampicillin or nystatin (Sigma) were used as a reference antibacterial or antifungal compounds, respectively.

Subsequently, MIC (Minimal Inhibitory Concentration) of the compounds was examined by the microdilution broth method, using their two-fold dilutions in Mueller-Hinton broth (for bacteria) and RPMI 1640 broth with MOPS (for fungi) prepared in the 96-well polystyrene plates. The final concentrations of the compounds ranged from 1000 to 0.488 μg/mL. Microbial suspensions were prepared in 0.85% NaCl with an optical density of 0.5 McFarland standard. Next each bacterial or fungal suspension was added per each well containing broth and various concentrations of the examined compounds. After incubation, the MIC was assessed spectrophotometrically as the lowest concentration of the samples exhibiting complete bacterial or fungal growth inhibition. Appropriate DMSO, growth and sterile controls were carried out. The medium with no tested substances was used as a control

The MBC (Minimal Bactericidal Concentration) and MFC (Minimal Fungicidal Concentration) are defined as the lowest concentration of the compounds that is required to kill a particular bacterial or fungal species. The MBC/MFC was determined by removing the culture used for the MIC determination from each well and spotted onto the appropriate agar medium. The plates were incubated under appropriate conditions for bacteria and fungi. The lowest compounds concentration with no visible growth observed was assessed as a bactericidal/fungicidal concentration. All the experiments were repeated three times and the representative data are presented [3,5].

In this study, no bioactivity was defined as a MIC > 1000 μg/mL, mild bioactivity as a MIC in the range 501 – 1000 μg/mL, moderate bioactivity with MIC from 126 to 500 μg/mL, good bioactivity as a MIC in the range 26 – 125 μg/mL, strong bioactivity with MIC between 10 and 25 μg/mL and very strong bioactivity as a MIC < 10 μg/mL [4]. The MBC/MIC or MFC/MIC ratios was calculated in order to determine bactericidal/fungicidal (MBC/MIC ≤ 4, MFC/MIC ≤ 4) or bacteriostatic/fungistatic (MBC/MIC > 4, MFC/MIC > 4) effect of the tested compounds.

References:

1. European Committee for Antimicrobial Susceptibility Testing (EUCAST) Determination of minimum inhibitory concentrations (MICs) of antibacterial agents by broth dilution. EUCAST discussion document E. Dis 5.1. *Clin. Microbiol. Infect.* **2003**, *9*, 1–7.
2. Clinical and Laboratory Standards Institute. Reference method for broth dilution antifungal susceptibility testing of yeasts. M27-S4. Clinical and Laboratory Standards Institute, Wayne, PA, USA, **2012**.
3. Popiołek Ł.; Biernasiuk A.; Malm A. Synthesis and antimicrobial activity of new 1,3-thiazolidin-4-one derivatives obtained from carboxylic acid hydrazides. *Phosphorus Sulfur Silicon Relat. Elem.* **2015**, *190(2)*, 251–260.

4. O'Donnell F.; Smyth T.J.; Ramachandran V.N.; Smyth W.F. A study of the antimicrobial activity of selected synthetic and naturally occurring quinolines. *Int. J. Antimicrob. Agents.* **2010**, *35*, 30–38.
5. Wiegand I.; Hilpert K.; Hancock R.E.W. Agar and broth dilution methods to determine the minimal inhibitory concentration (MIC) of antimicrobial substances. *Nat. Protoc.* **2008**, *3(2)*, 163–175.

3. Cytotoxicity

Table S7. The cell proliferation in % after 24h exposition on studied compounds in L929 cell line.

Concentration (µM) / Compound No	10		25		50		75		100		control	
	Repeat		Repeat		Repeat		Repeat		Repeat		Repeat	
	No 1	No 2	No 1	No 2	No 1	No 2	No 1	No 2	No 1	No 2	No 1	No 2
**14**	94	94	100	89	85	73	77	69	91	97	99	105
**20**	99	104	89	95	87	112	103	96	86	94	96	92
**21**	96	102	101	97	100	106	118	109	89	96	109	87
**27**	101	89	96	92	95	81	103	107	92	91	93	111

Table S8. The cell proliferation in % after 48h exposition on studied compounds in L929 cell line.

Concentration (µM) / Compound No	10		25		50		75		100		control	
	Repeat		Repeat		Repeat		Repeat		Repeat		Repeat	
	No 1	No 2	No 1	No 2	No 1	No 2	No 1	No 2	No 1	No 2	No 1	No 2
**14**	75	99	106	99	83	98	83	89	94	101	104	98
**20**	91	102	88	93	94	99	94	99	87	84	102	94
**21**	94	98	89	97	89	88	104	96	89	84	99	87
**27**	92	89	95	99	92	91	99	92	87	92	102	100

Table S9. The cell proliferation in % after 24h exposition on studied compounds in A549 cell line.

Concentration (µM) / Compound No	10		25		50		75		100		control	
	Repeat		Repeat		Repeat		Repeat		Repeat		Repeat	
	No 1	No 2	No 1	No 2	No 1	No 2	No 1	No 2	No 1	No 2	No 1	No 2
**14**	86	77	103	98	89	84	87	89	82	74	88	92
**20**	87	92	83	91	83	87	91	95	90	93	88	96
**21**	88	99	88	88	92	101	103	99	87	94	82	103
**27**	95	92	97	89	94	87	87	92	109	103	89	98

Table S10. The cell proliferation in % after 48h exposition on studied compounds in A549 cell line.

Concentration (µM) / Compound No	10		25		50		75		100		control	
	Repeat		Repeat		Repeat		Repeat		Repeat		Repeat	
	No 1	No 2	No 1	No 2	No 1	No 2	No 1	No 2	No 1	No 2	No 1	No 2
**14**	93	79	87	91	91	83	89	89	90	96	86	90
**20**	88	84	79	83	88	92	99	95	91	93	101	97
**21**	92	99	94	91	97	91	83	87	91	87	99	84
**27**	84	87	87	82	88	89	91	87	98	95	95	92

Table S11. The cell proliferation in % after 24h exposition on studied compounds in HeLa cell line.

**Concentration (µM) / Compound No**	**10**		**25**		**50**		**75**		**100**		**control**	
	Repeat		Repeat		Repeat		Repeat		Repeat		Repeat	
	No 1	No 2	No 1	No 2	No 1	No 2	No 1	No 2	No 1	No 2	No 1	No 2
**14**	88	81	93	91	88	82	98	89	82	89	89	93
**20**	87	92	81	88	78	83	85	91	81	79	92	104
**21**	89	88	89	92	86	104	104	87	87	91	98	87
**27**	74	83	69	73	61	67	72	69	63	59	87	95

Table S12. The cell proliferation in % after 48h exposition on studied compounds in HeLa cell line.

**Concentration (µM) / Compound No**	**10**		**25**		**50**		**75**		**100**		**control**	
	Repeat		Repeat		Repeat		Repeat		Repeat		Repeat	
	No 1	No 2	No 1	No 2	No 1	No 2	No 1	No 2	No 1	No 2	No 1	No 2
**14**	76	79	82	89	85	84	77	71	81	93	89	101
**20**	88	91	73	75	87	91	99	96	93	94	100	103
**21**	86	93	101	97	81	87	91	89	88	92	109	104
**27**	77	82	82	71	61	49	51	43	63	57	95	103

Table S13. The cell proliferation in % after 24h exposition on studied compounds in T47D cell line.

**Concentration (µM) / Compound No**	**10**		**25**		**50**		**75**		**100**		**control**	
	Repeat		Repeat		Repeat		Repeat		Repeat		Repeat	
	No 1	No 2	No 1	No 2	No 1	No 2	No 1	No 2	No 1	No 2	No 1	No 2
**14**	86	89	71	74	79	75	87	80	79	73	91	87
**20**	78	81	76	72	77	69	77	73	87	82	91	93
**21**	82	84	83	88	78	76	71	79	73	78	99	89
**27**	96	91	91	82	83	79	84	87	86	91	90	87

Table S14. The cell proliferation in % after 48h exposition on studied compounds in T47D cell line.

**Concentration (µM) / Compound No**	**10**		**25**		**50**		**75**		**100**		**control**	
	Repeat		Repeat		Repeat		Repeat		Repeat		Repeat	
	No 1	No 2	No 1	No 2	No 1	No 2	No 1	No 2	No 1	No 2	No 1	No 2
**14**	71	79	81	79	81	79	72	69	74	69	89	93
**20**	80	78	79	75	76	73	83	86	81	79	94	97
**21**	79	71	79	81	81	79	69	74	78	71	89	94
**27**	82	75	88	84	81	80	79	72	83	81	99	92

Figure S6. The cell proliferation in % after 24h exposition on the compound **14** in L929 cell line.

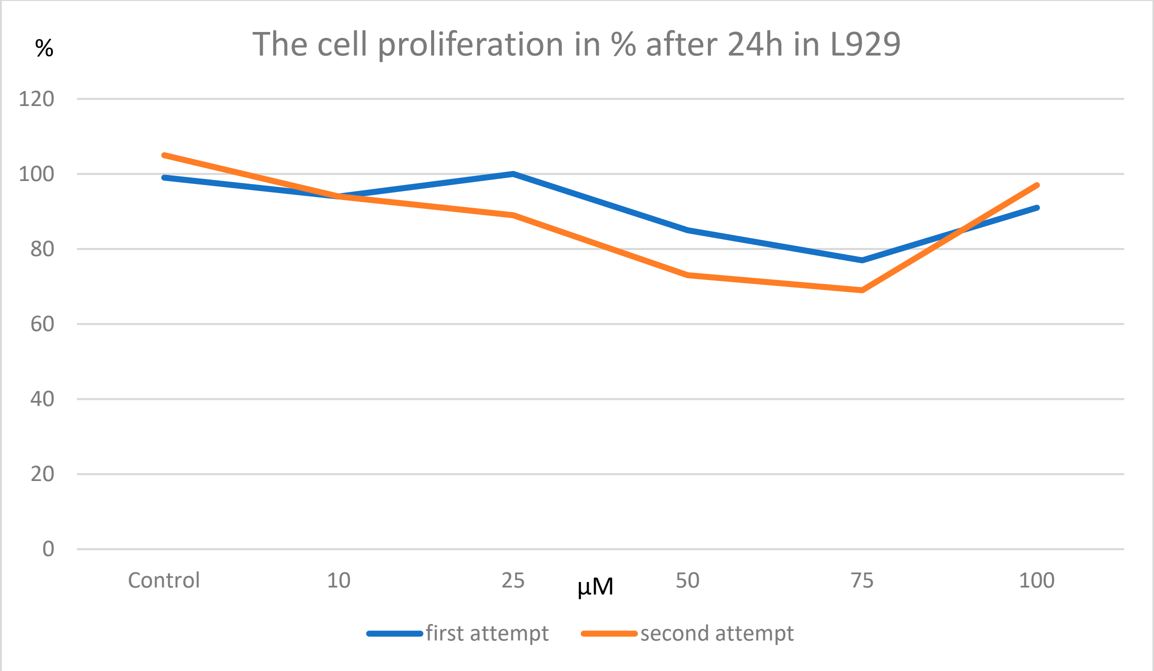

Figure S7. The cell proliferation in % after 24h exposition on the compound **20** in L929 cell line.

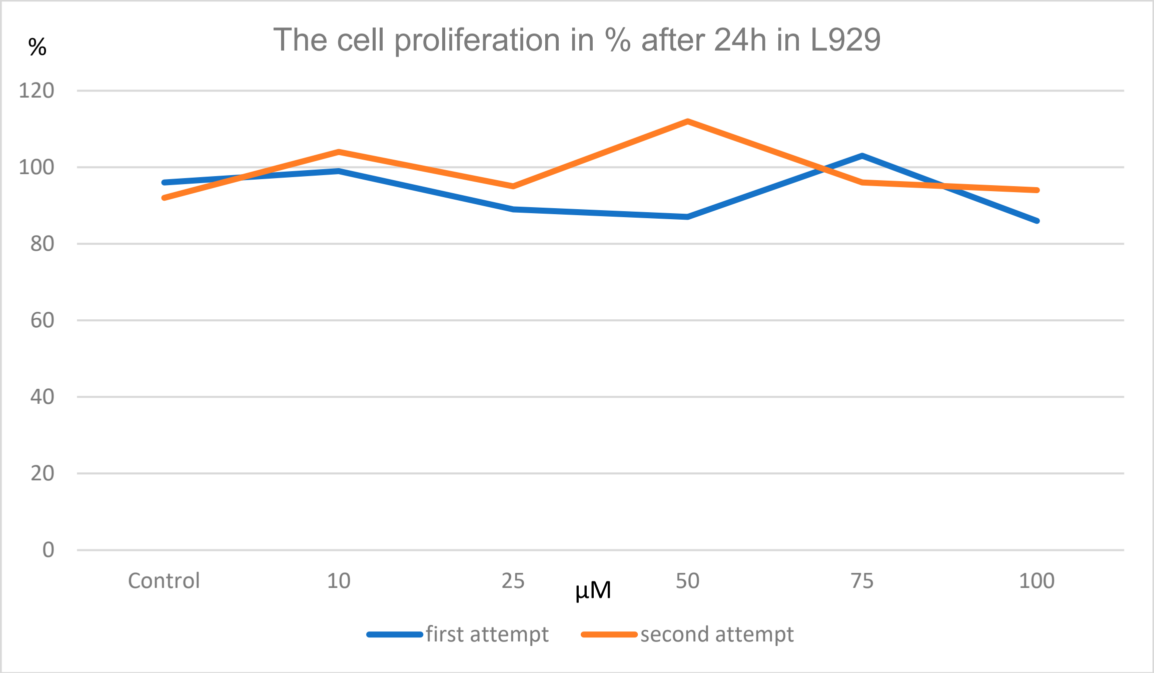

Figure S8. The cell proliferation in % after 24h exposition on the compound **21** in L929 cell line.

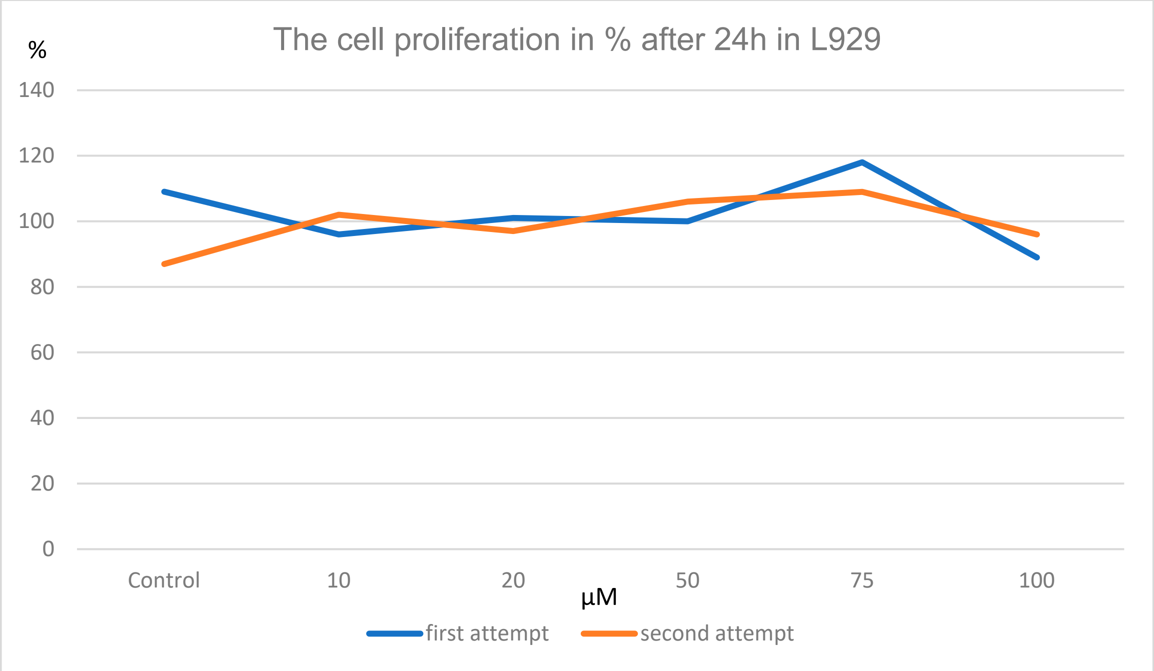

Figure S9. The cell proliferation in % after 24h exposition on the compound **27** in L929 cell line.

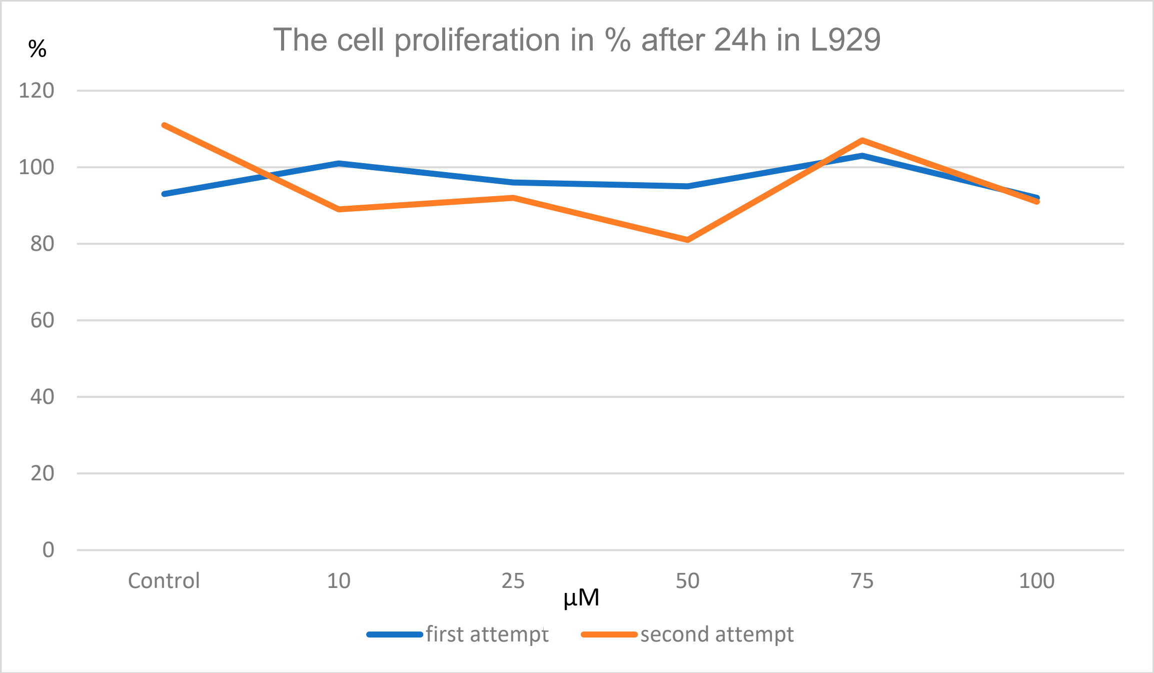

Figure S10. The cell proliferation in % after 48h exposition on the compound **14** in L929 cell line.

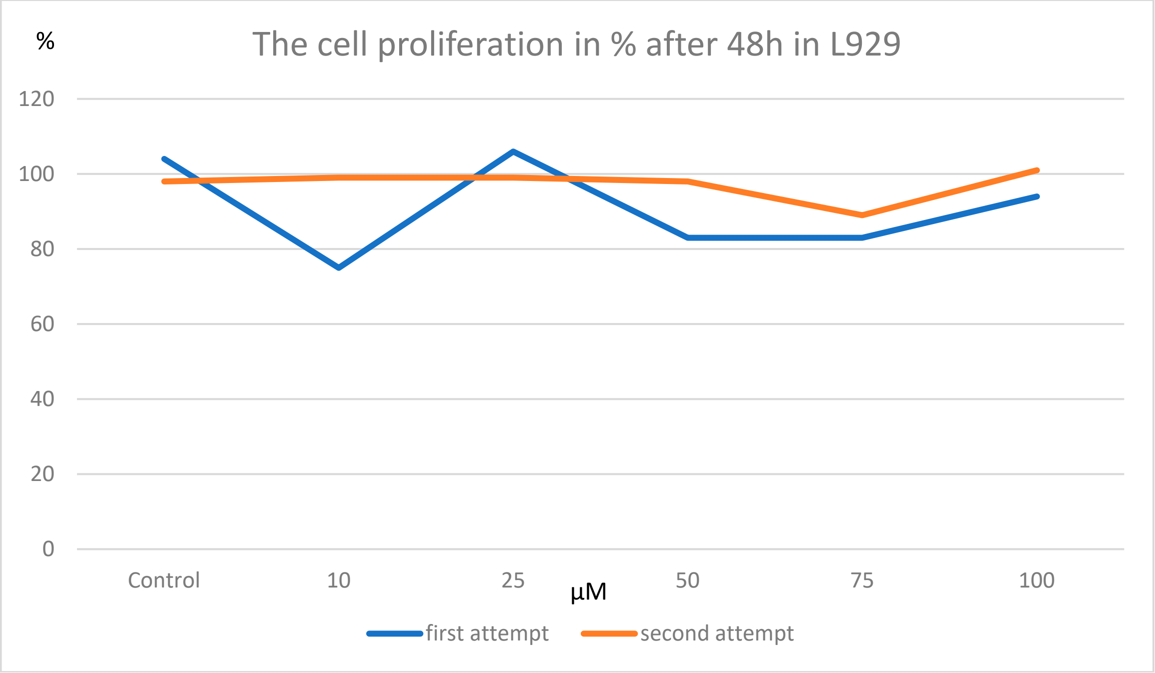

Figure S11 The cell proliferation in % after 48h exposition on the compound **20** in L929 cell line.

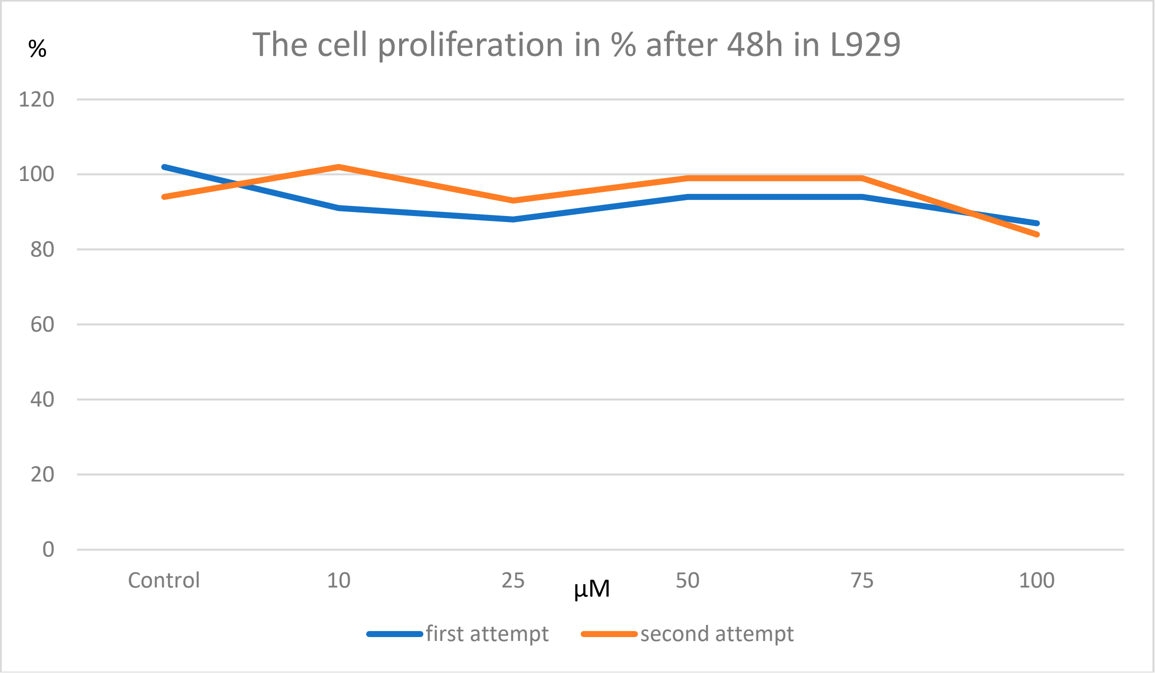

Figure S12. The cell proliferation in % after 48h exposition on the compound **21** in L929 cell line.

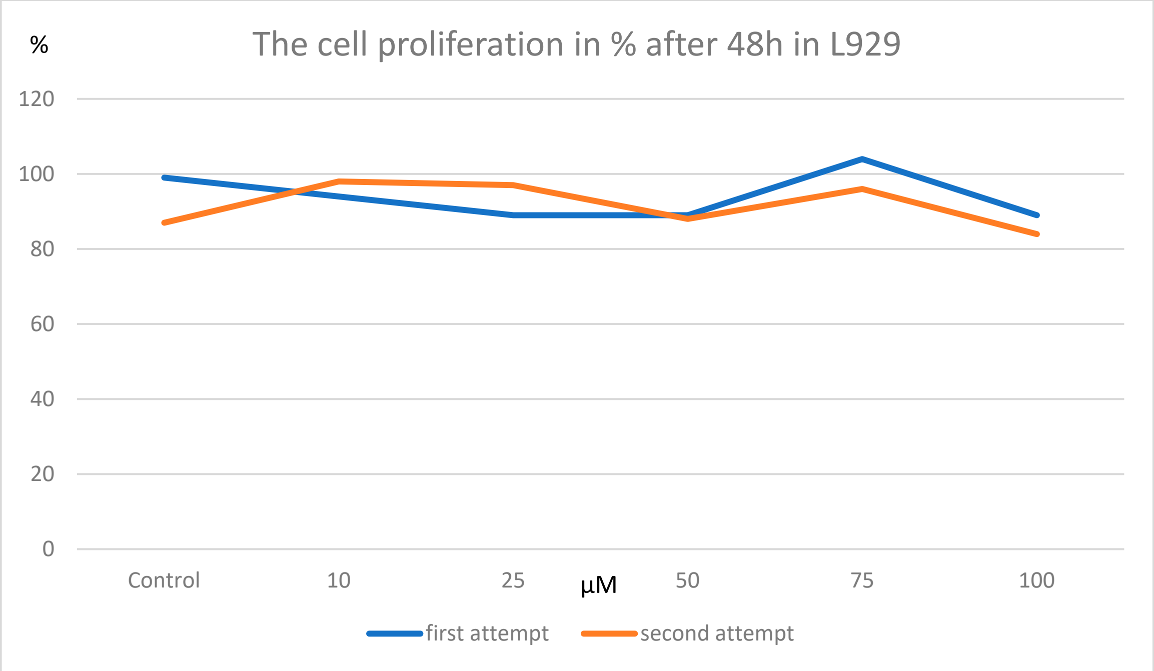

Figure S13. The cell proliferation in % after 48h exposition on the compound **27** in L929 cell line.

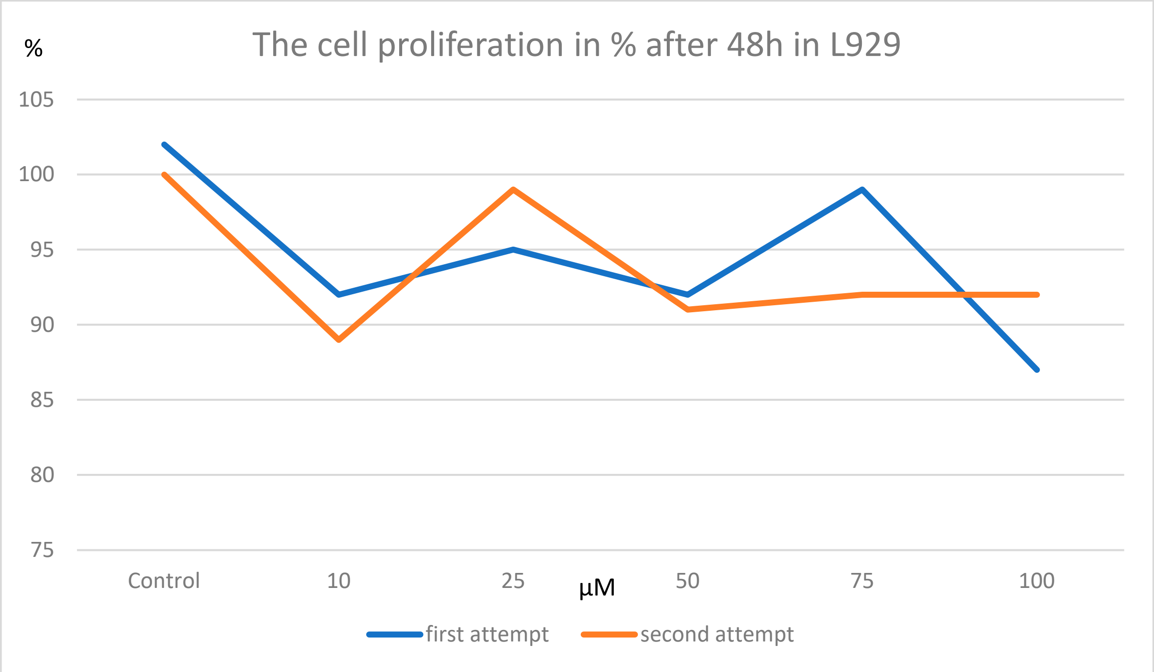

Figure S14. The cell proliferation in % after 24h exposition on the compound **14** in A549 cell line.

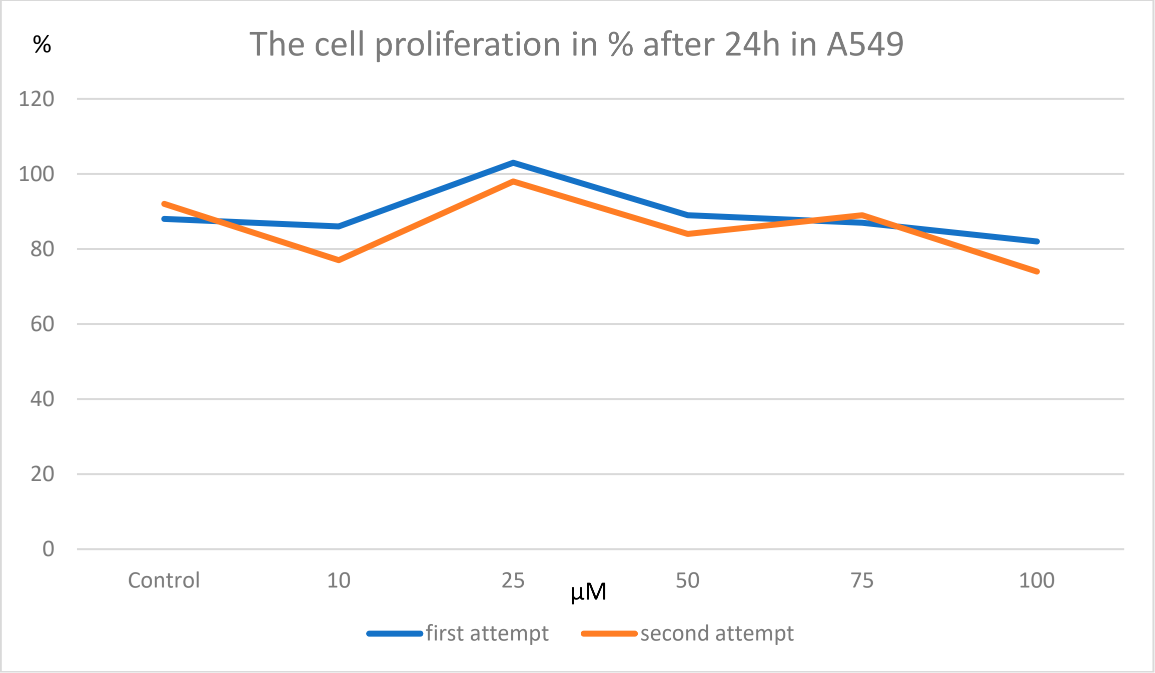

Figure S15. The cell proliferation in % after 24h exposition on the compound **20** in A549 cell line.

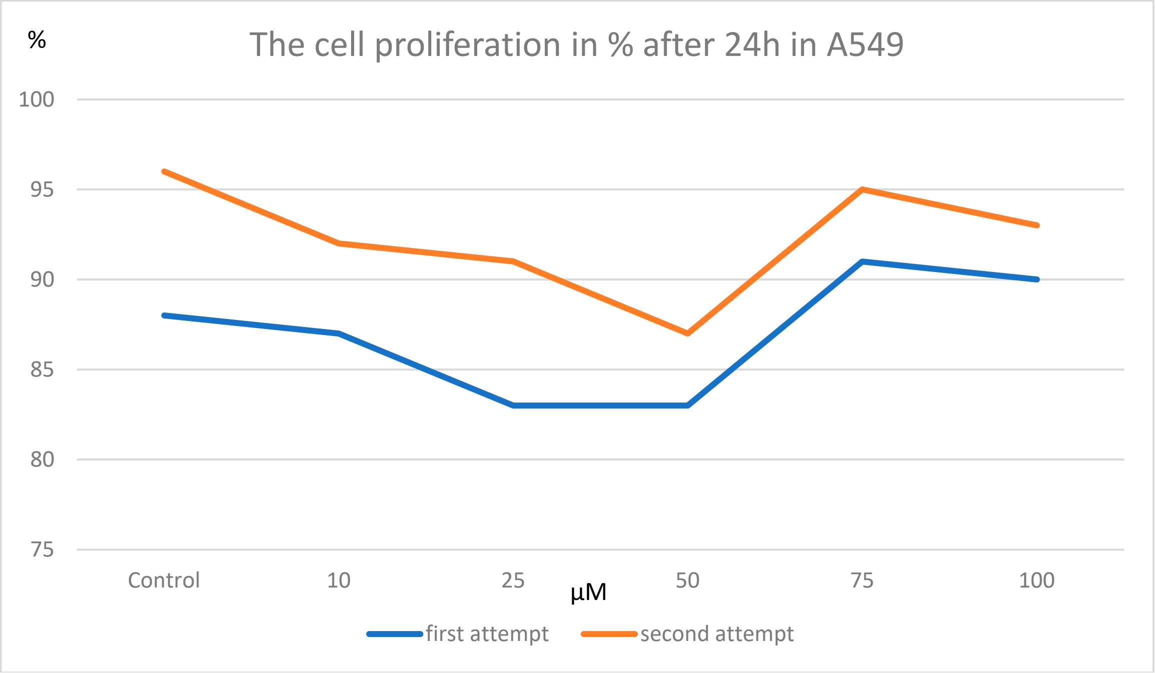

Figure S16. The cell proliferation in % after 24h exposition on the compound **21** in A549 cell line.

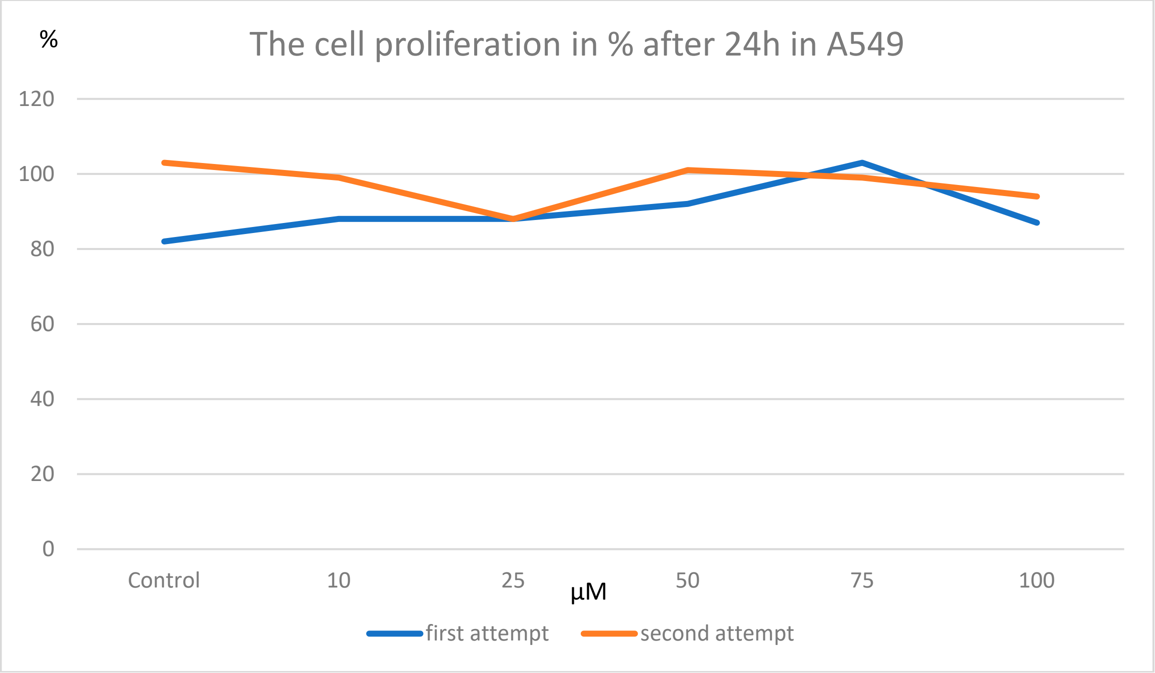

Figure S17. The cell proliferation in % after 24h exposition on the compound **27** in A549 cell line.

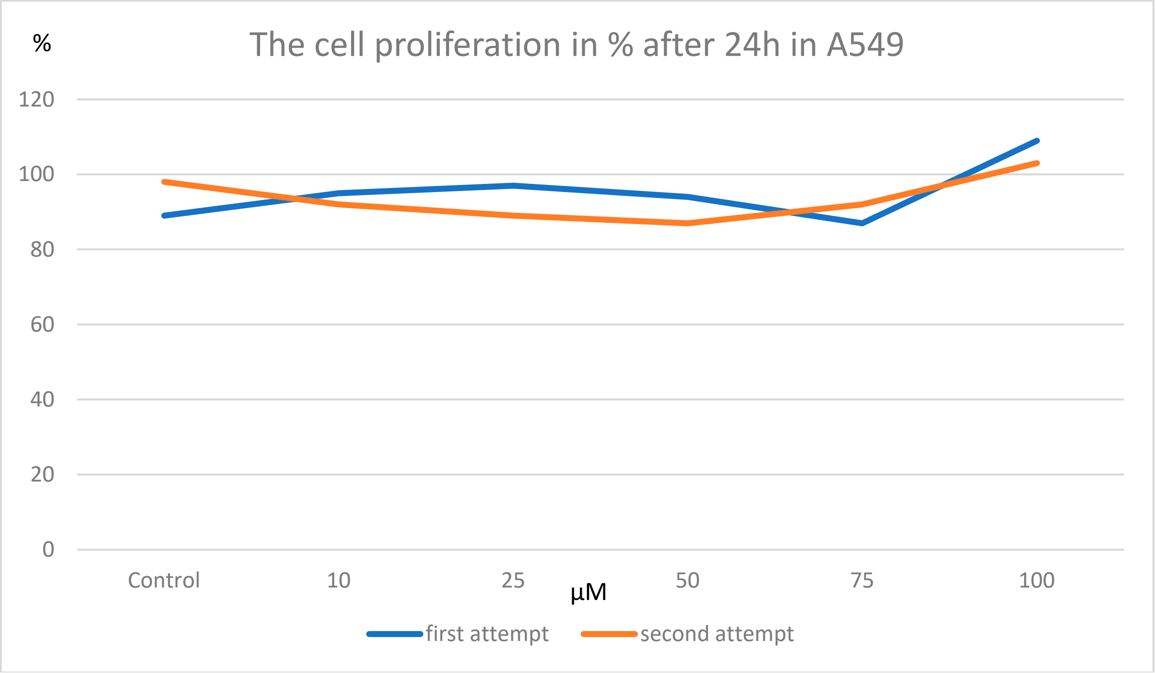

Figure S18. The cell proliferation in % after 48h exposition on the compound **14** in A549 cell line.

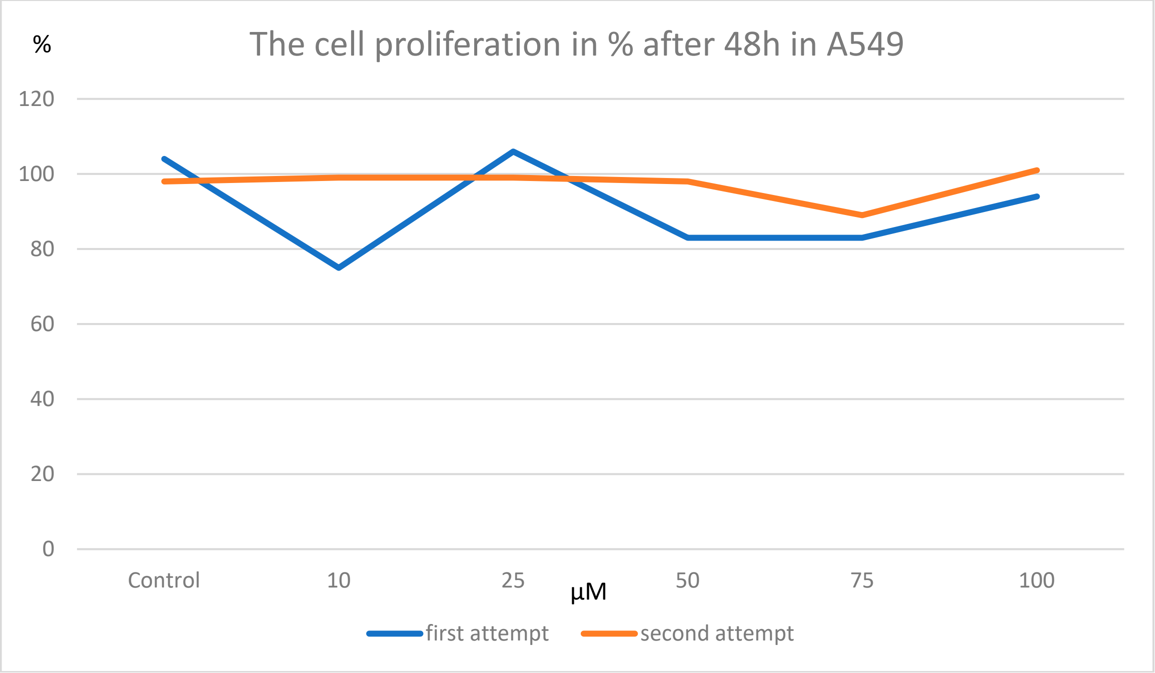

Figure S19. The cell proliferation in % after 48h exposition on the compound **20** in A549 cell line.

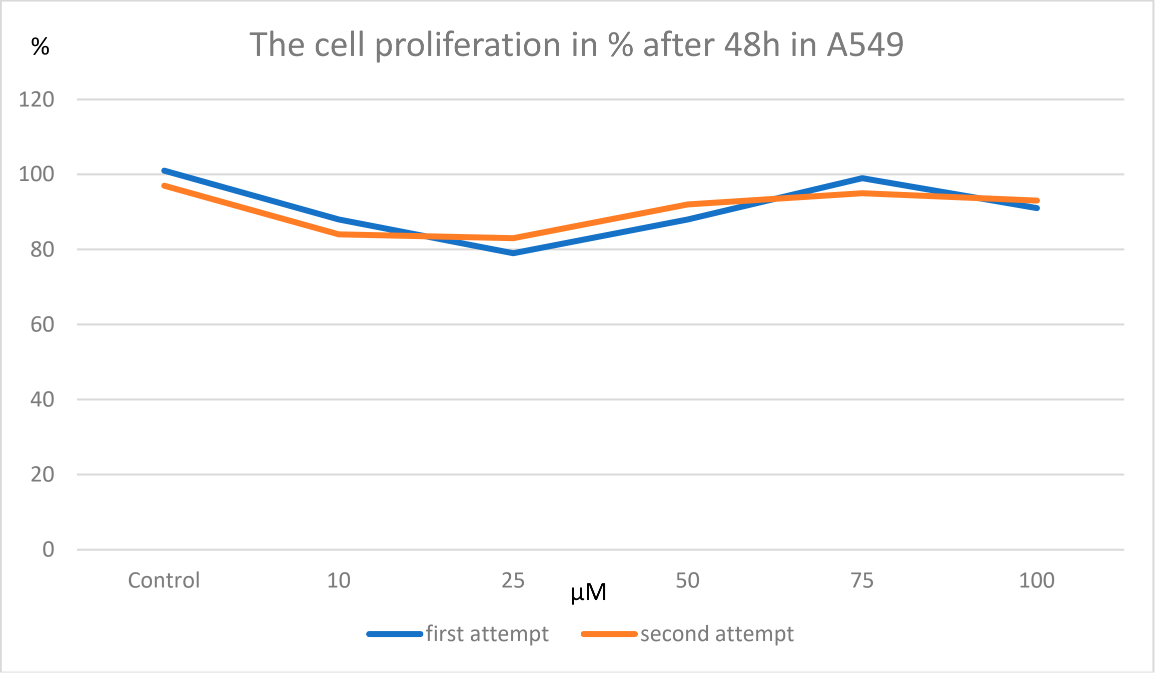

Figure S20. The cell proliferation in % after 48h exposition on the compound **21** in A549 cell line.

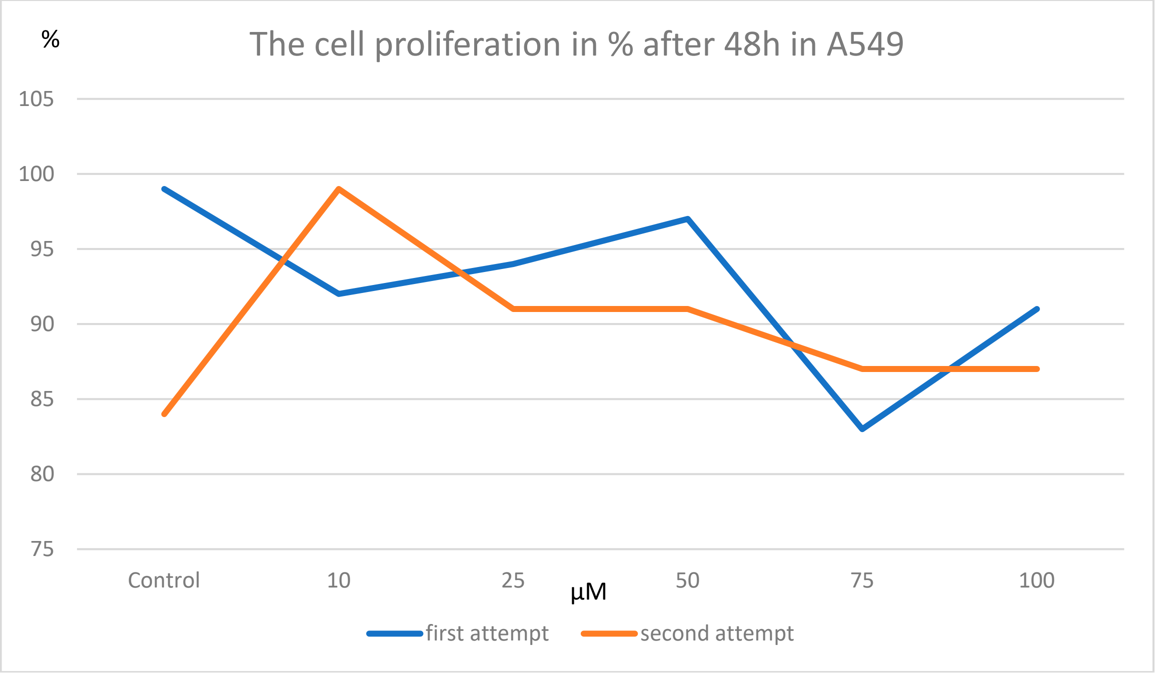

Figure S21. The cell proliferation in % after 48h exposition on the compound **27** in A549 cell line.

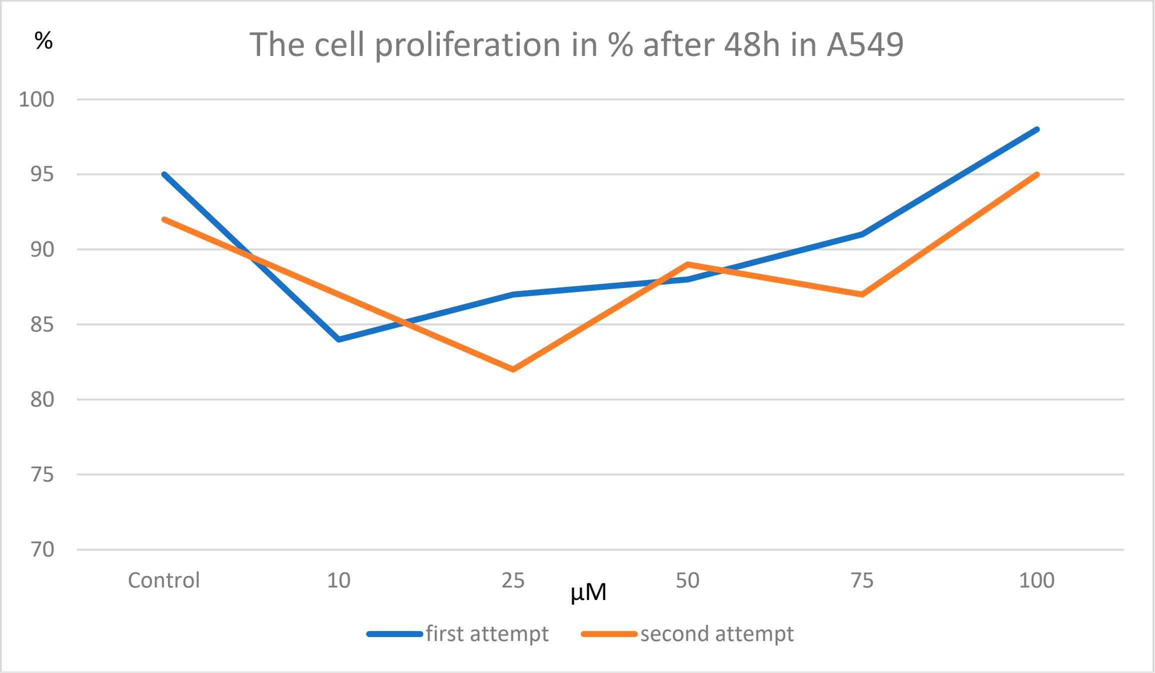

Figure S22. The cell proliferation in % after 24h exposition on the compound **14** in HeLa cell line.

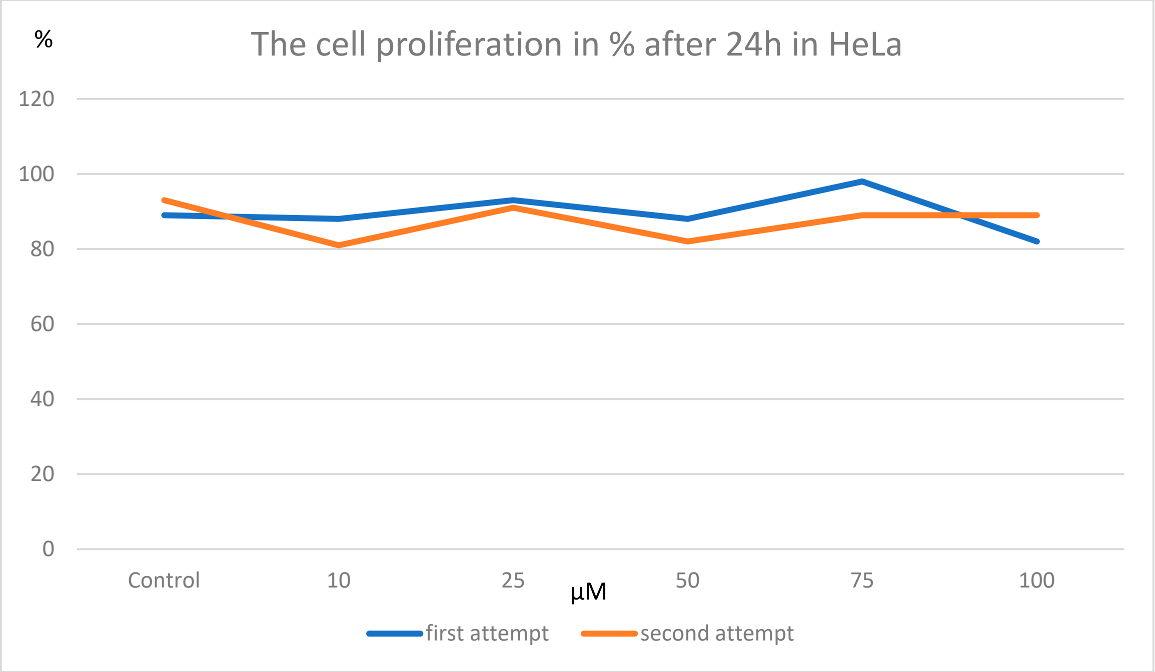

Figure S23. The cell proliferation in % after 24h exposition on the compound **20** in HeLa cell line.

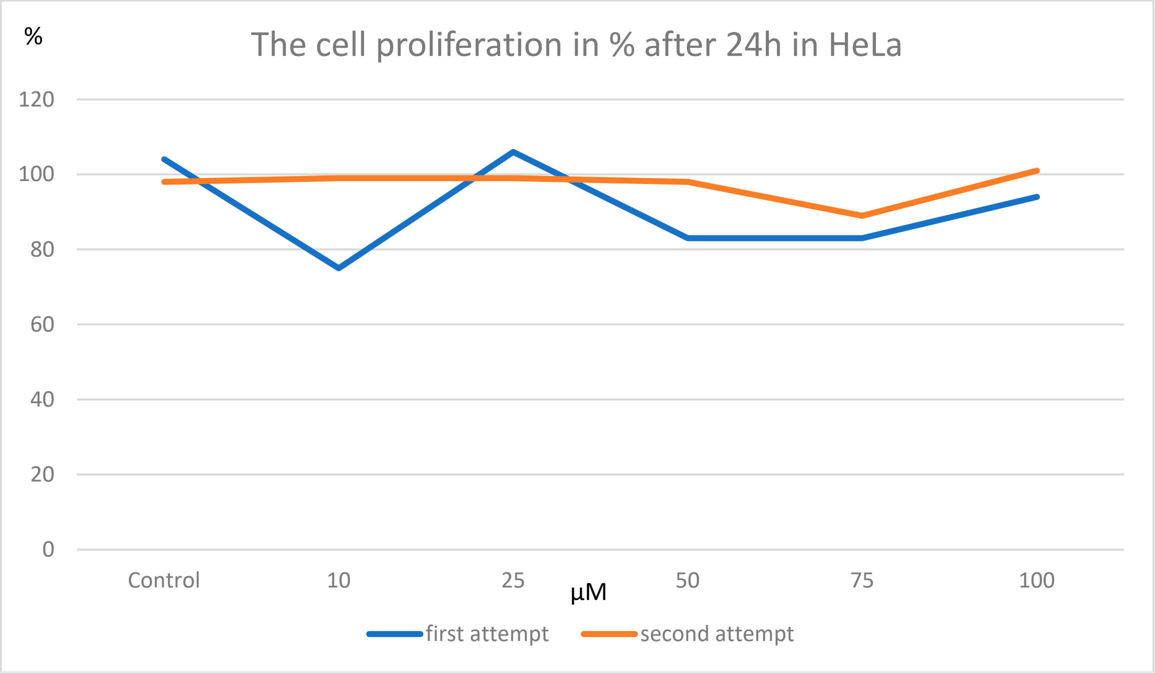

Figure S24. The cell proliferation in % after 24h exposition on the compound **21** in HeLa cell line.

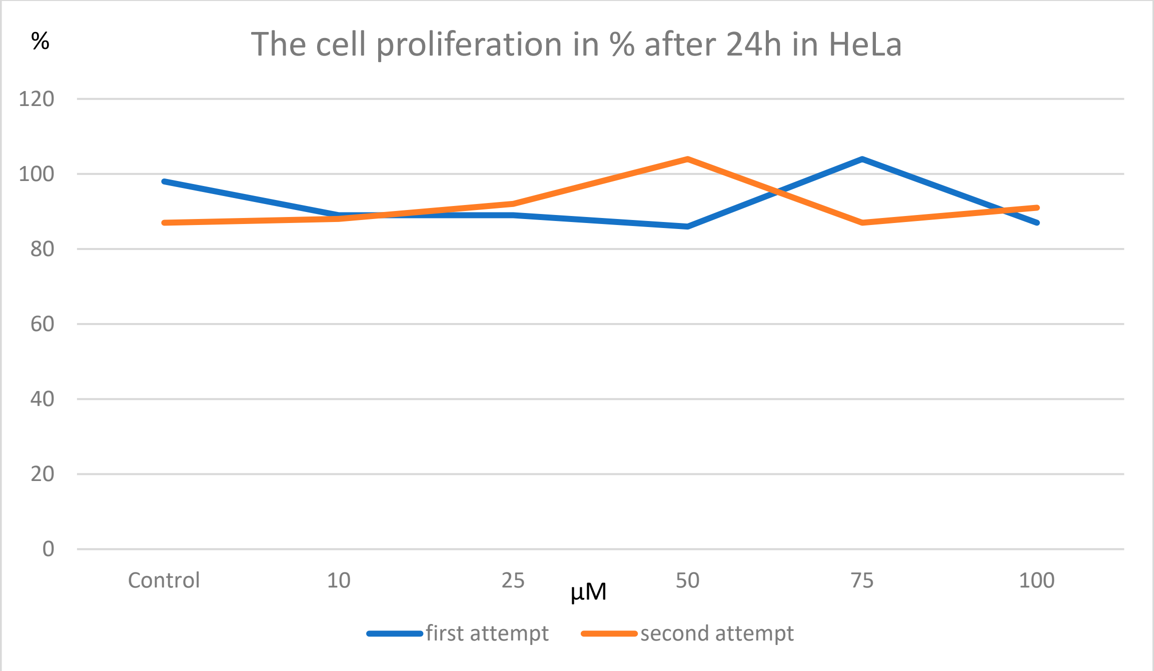

Figure S25. The cell proliferation in % after 24h exposition on the compound **27** in HeLa cell line.

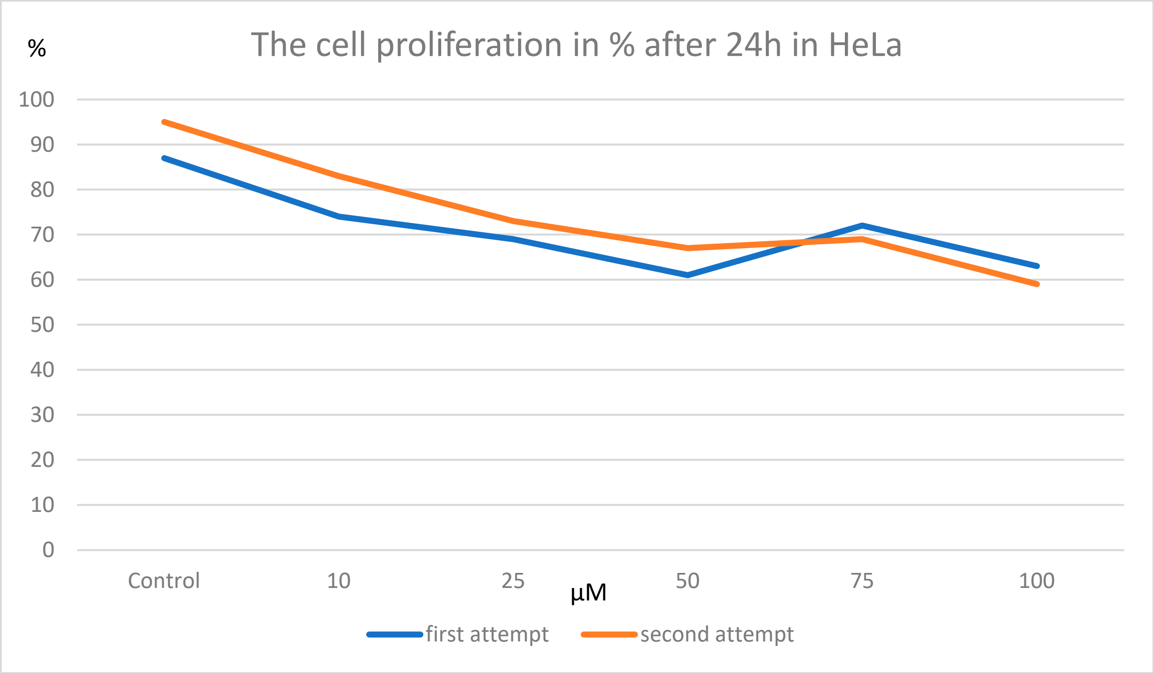

Figure S26. The cell proliferation in % after 48h exposition on the compound **14** in HeLa cell line.

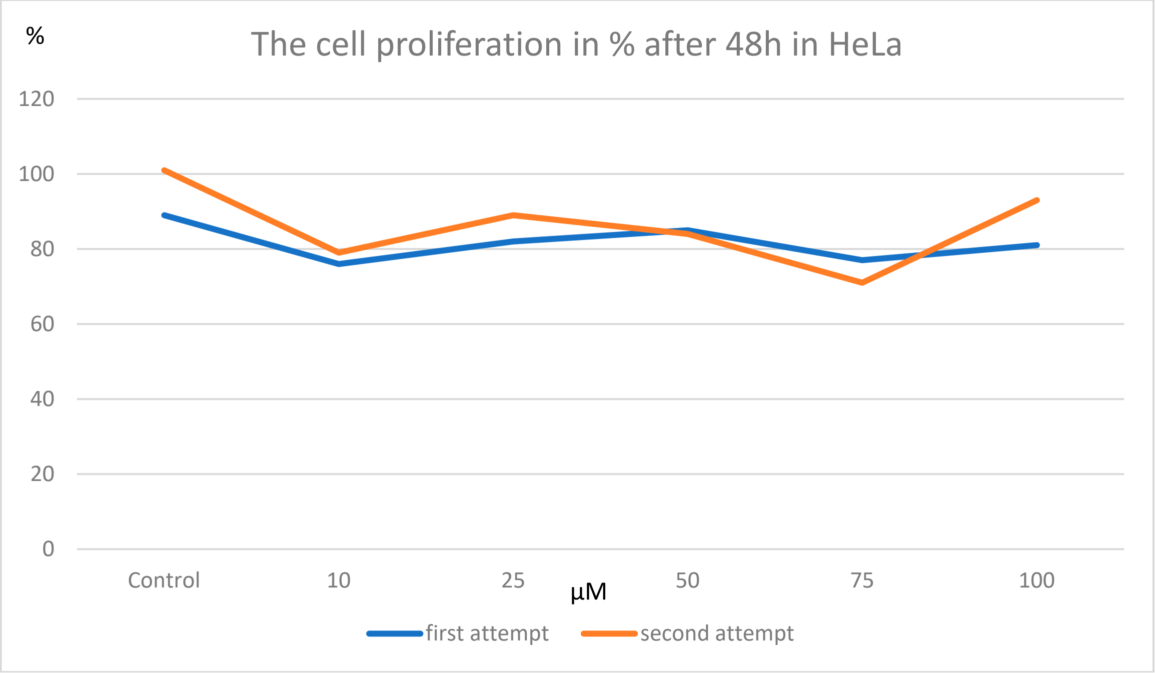

Figure S27. The cell proliferation in % after 48h exposition on the compound **20** in HeLa cell line.

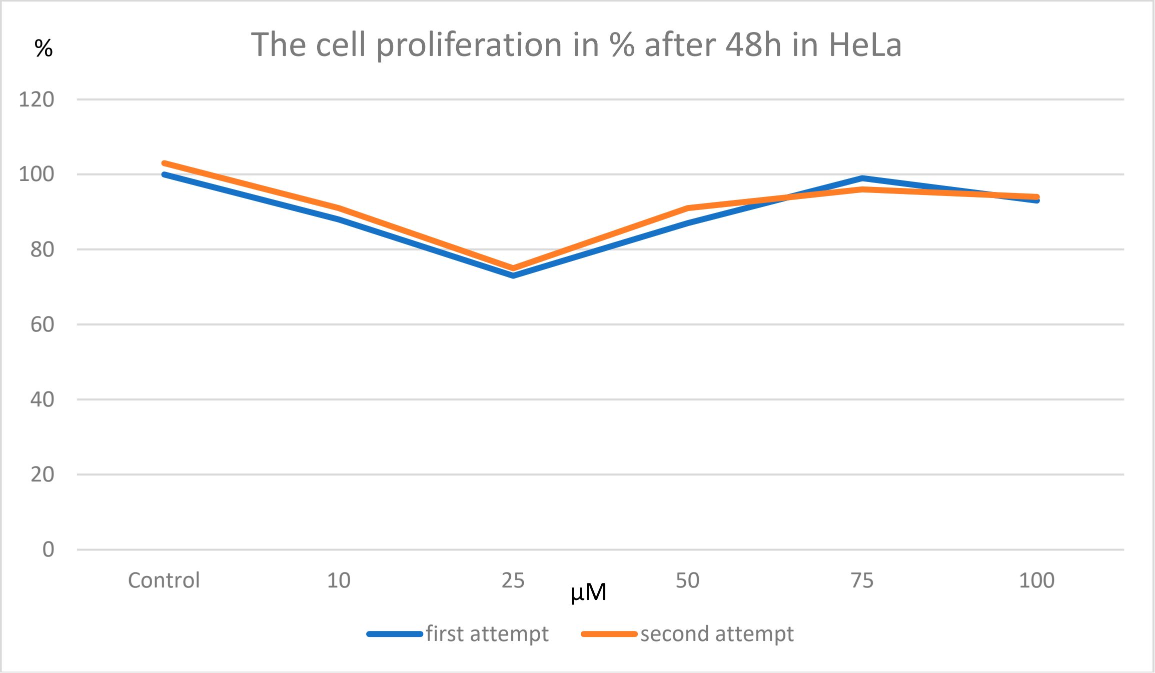

Figure S28. The cell proliferation in % after 48h exposition on the compound **21** in HeLa cell line.

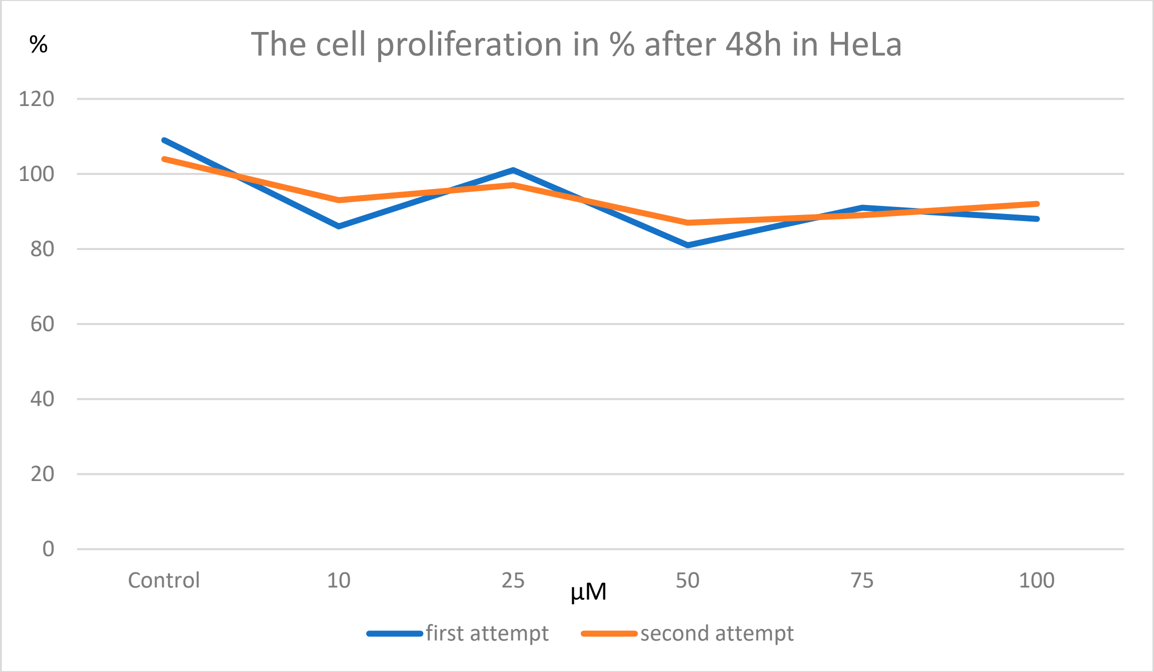

Figure S29. The cell proliferation in % after 48h exposition on the compound **27** in HeLa cell line.

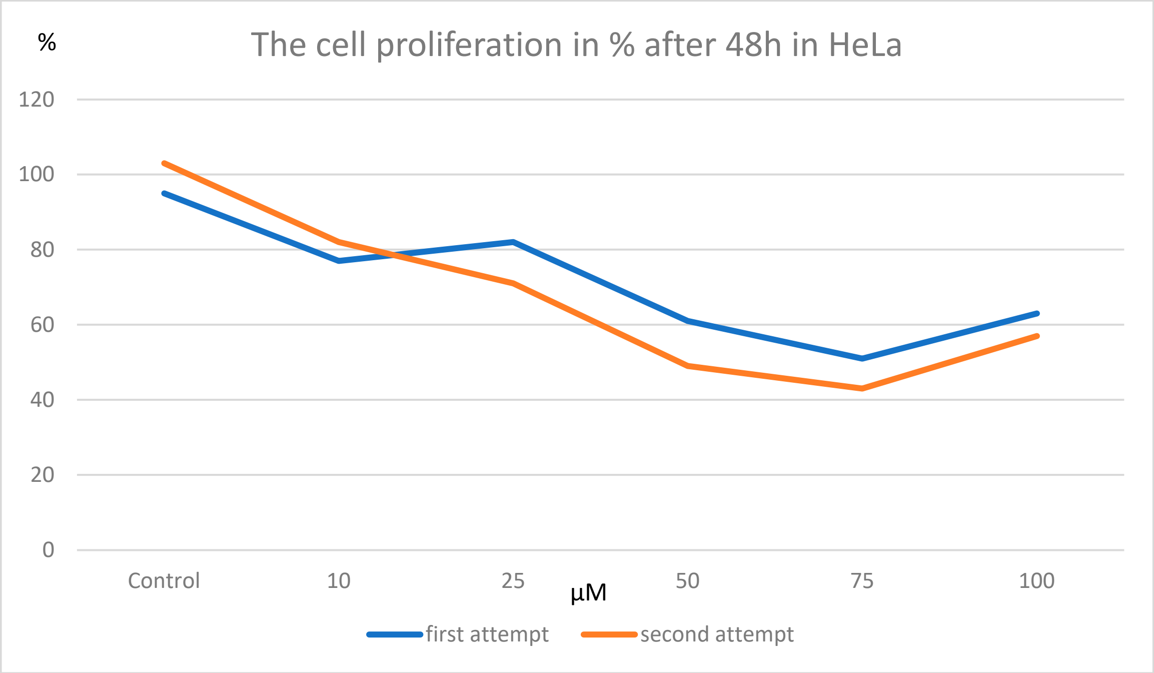

Figure S30. The cell proliferation in % after 24h exposition on the compound **14** in T47D cell line.

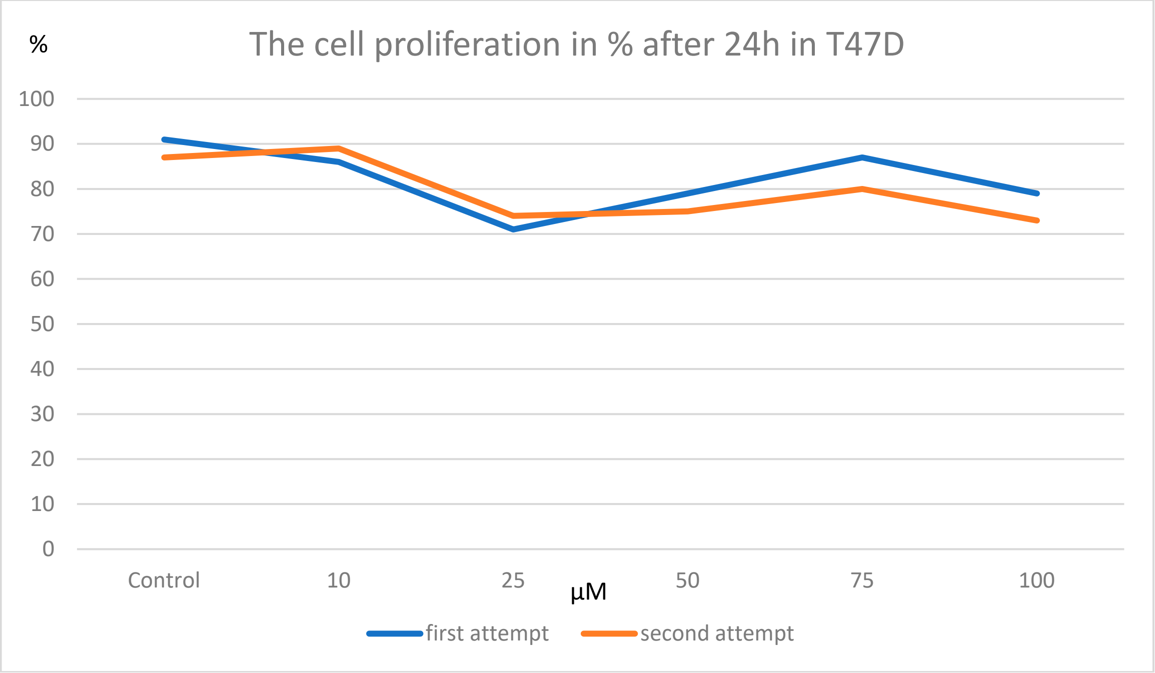

Figure S31. The cell proliferation in % after 24h exposition on the compound **20** in T47D cell line.

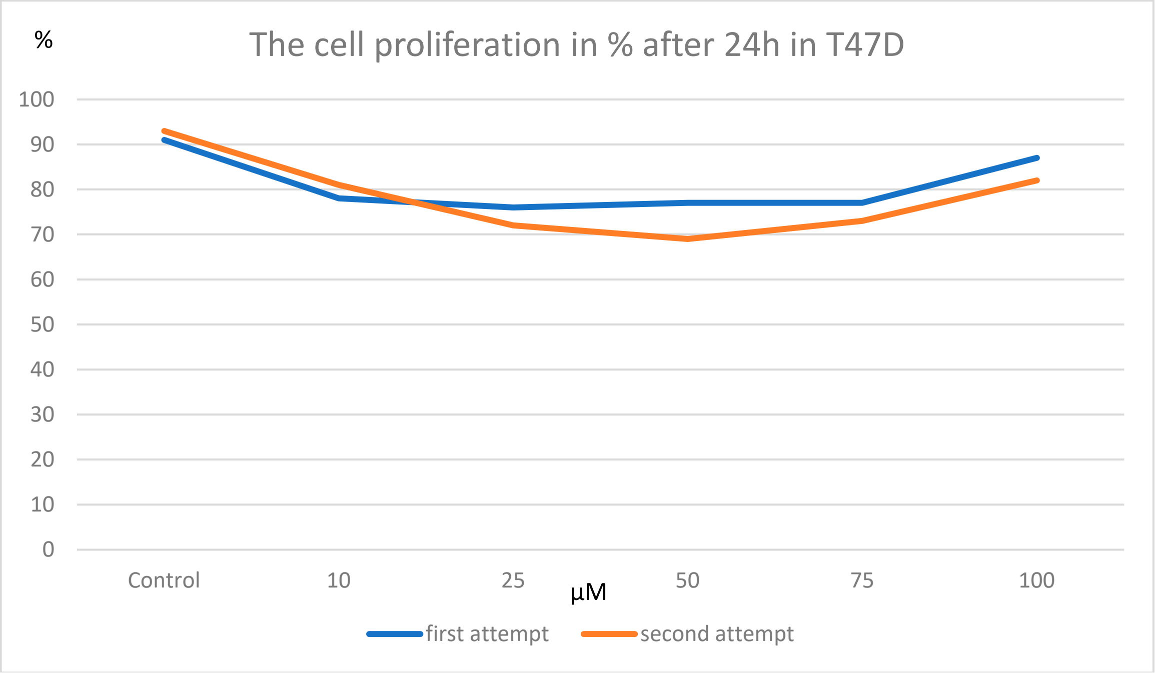

Figure S32. The cell proliferation in % after 24h exposition on the compound **21** in T47D cell line.

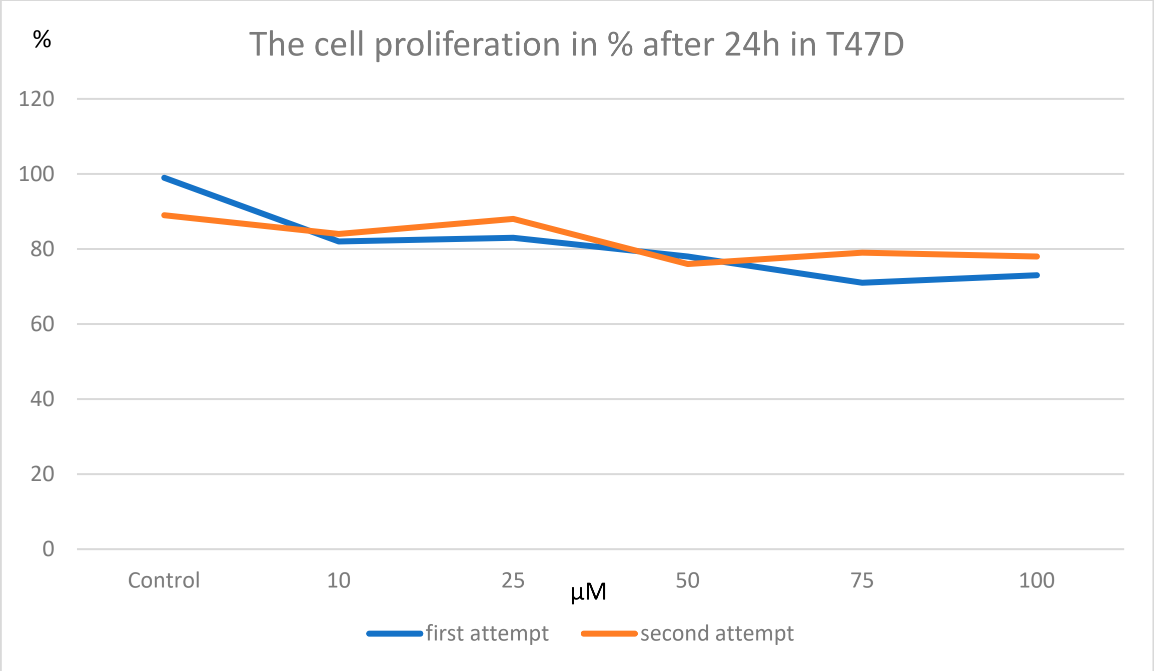

Figure S33. The cell proliferation in % after 24h exposition on the compound **27** in T47D cell line.

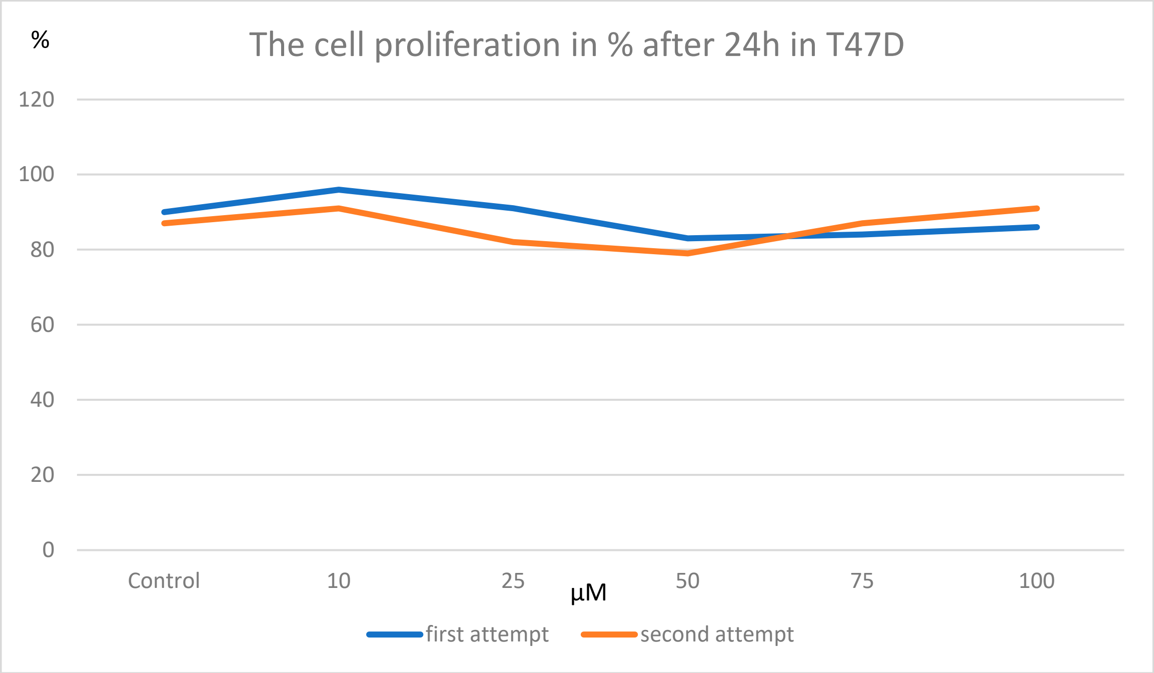

Figure S34. The cell proliferation in % after 48h exposition on the compound **14** in T47D cell line.

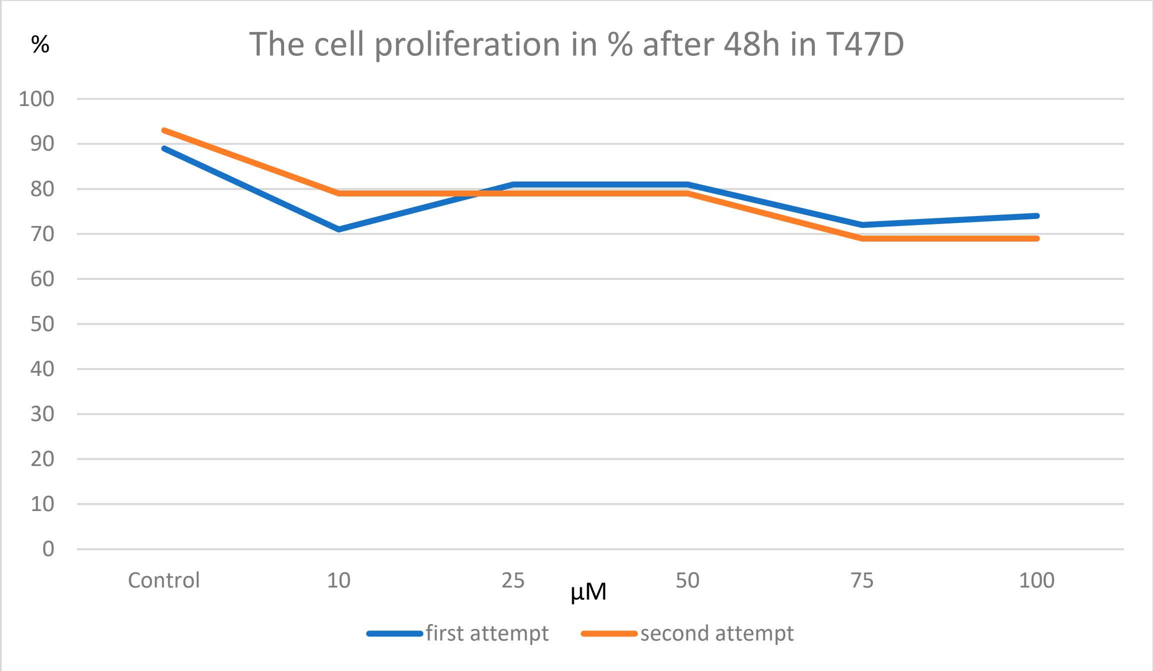

Figure S35. The cell proliferation in % after 48h exposition on the compound **20** in T47D cell line.

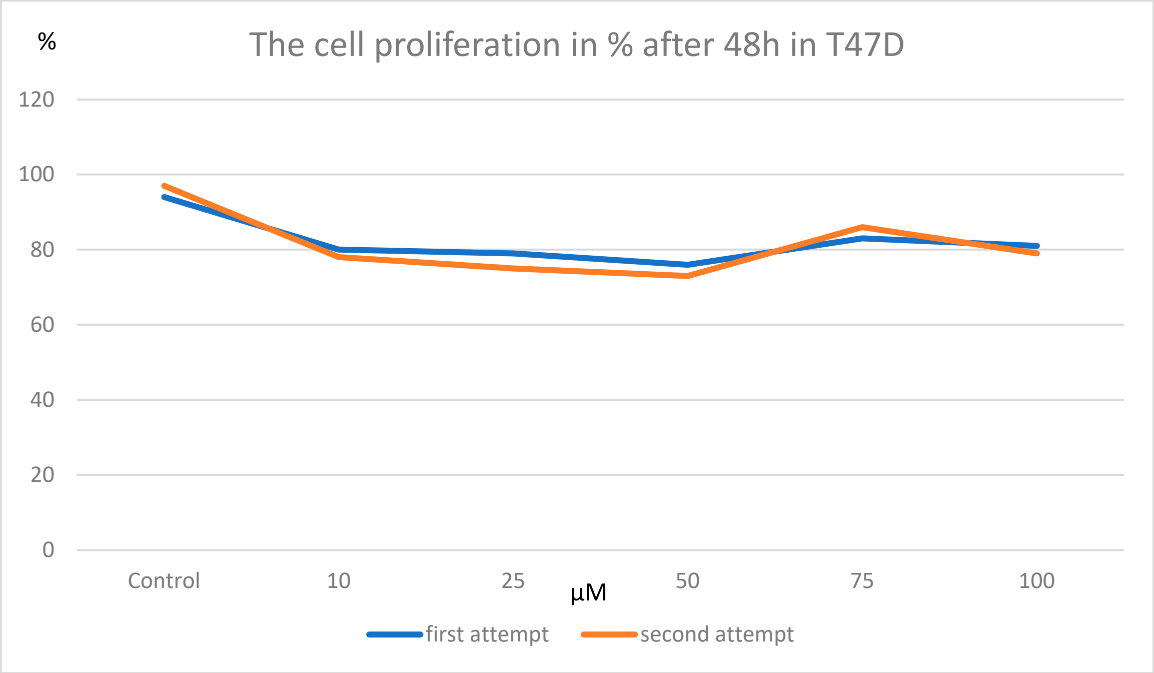

Figure S36. The cell proliferation in % after 48h exposition on the compound **21** in T47D cell line.

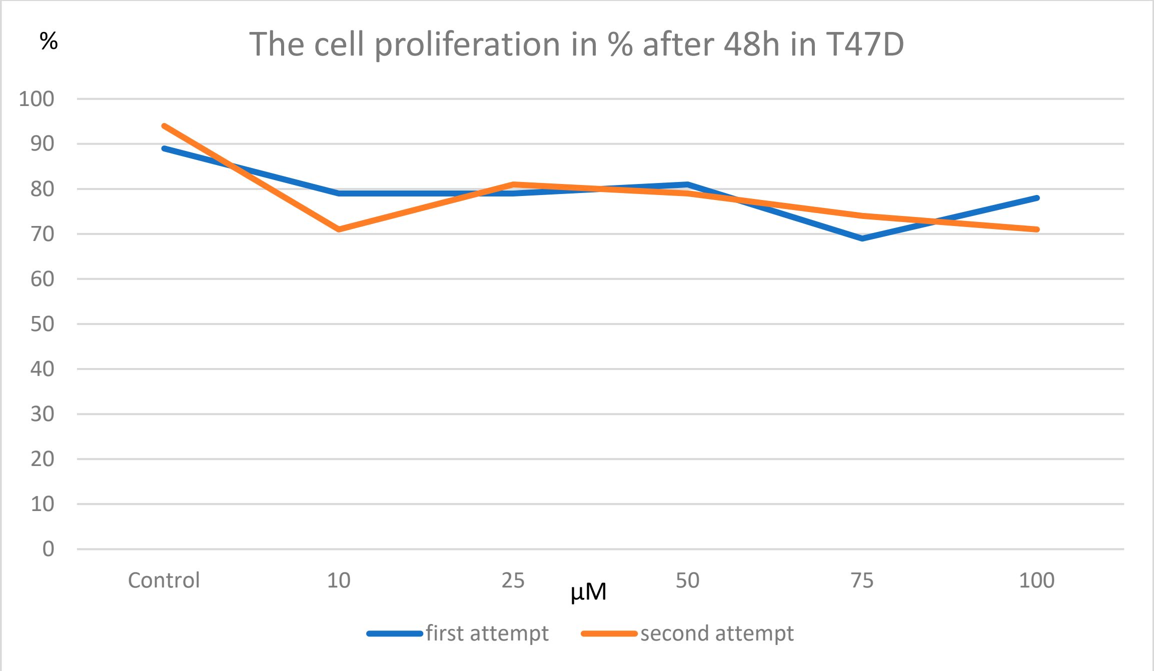

Figure S37. The cell proliferation in % after 48h exposition on the compound **27** in T47D cell line.

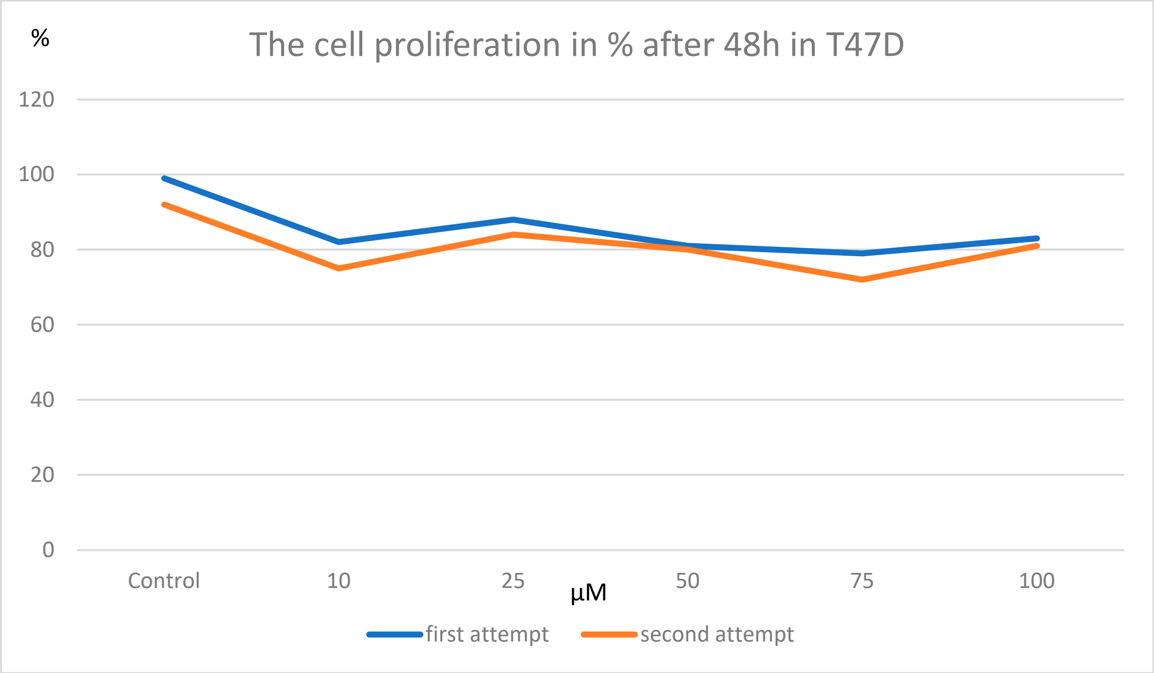

4. Examples of IR, ^{1}H NMR and ^{13}C NMR of synthesized hydrazides and acylhydrazones

Figure S38. The IR spectra of compound **4**: 2-iodobenzohydrazide

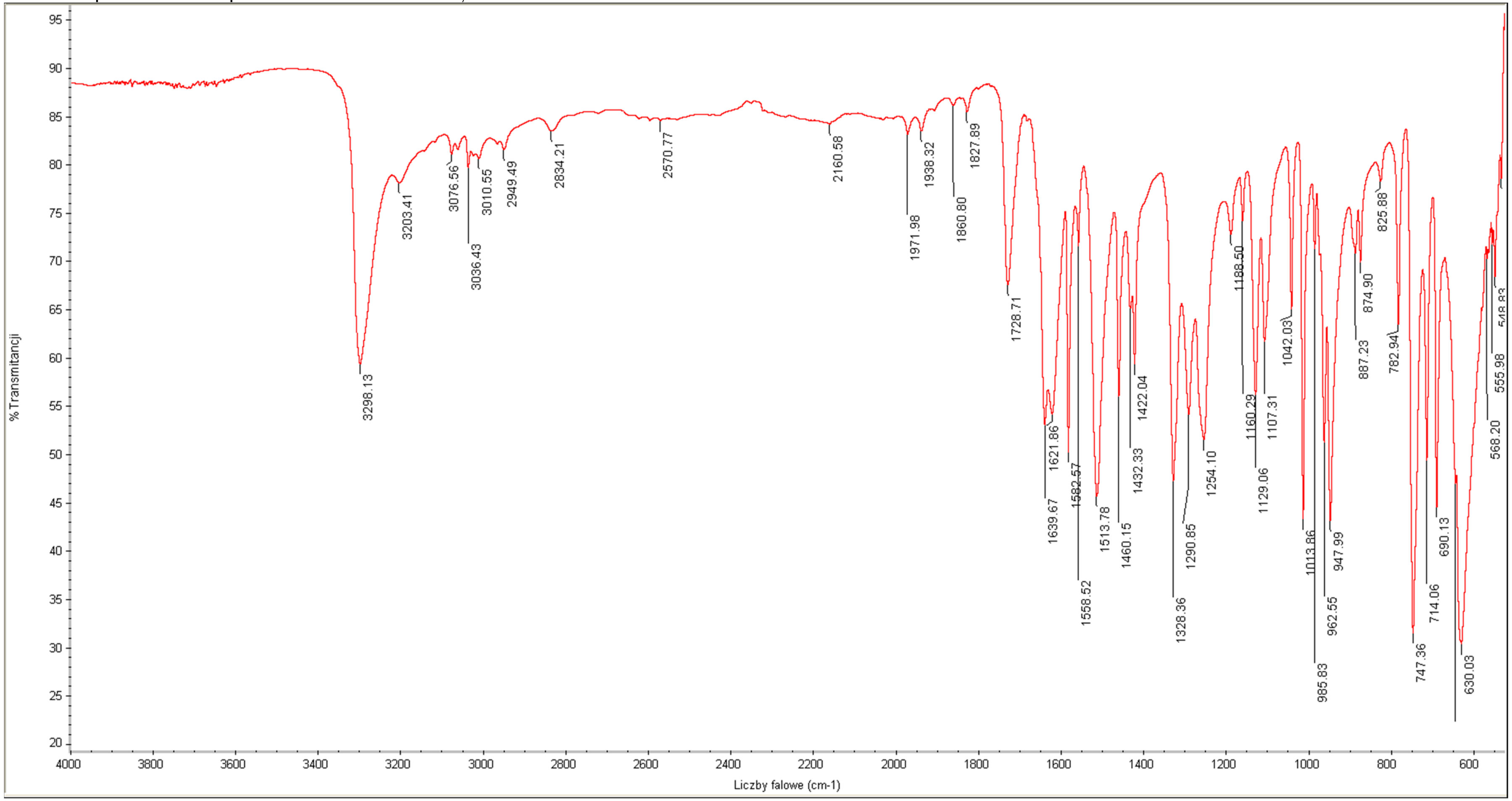

Figure S39. The ¹H NMR spectra of compound **4**: 2-iodobenzohydrazide

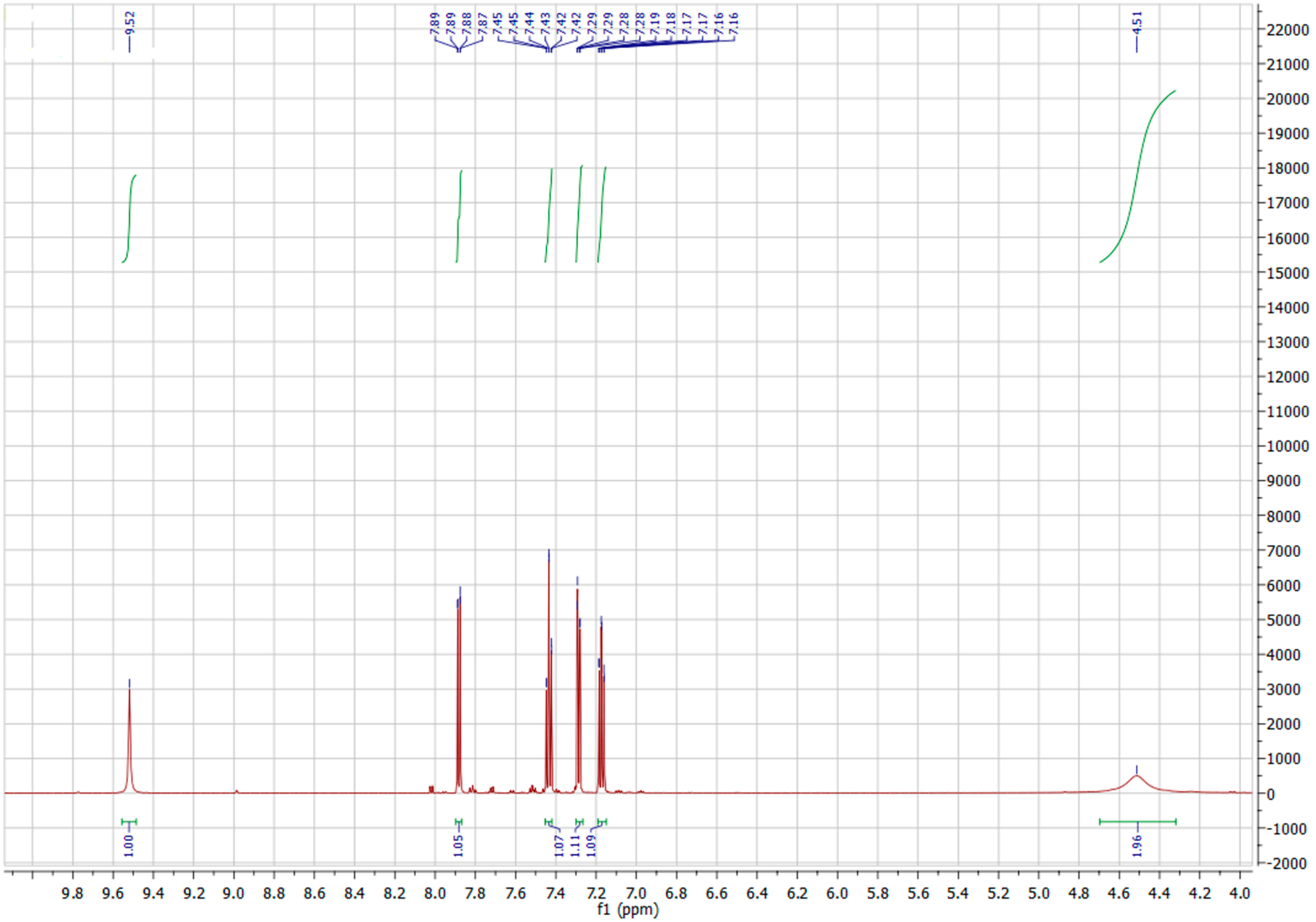

Figure S40. The ^{13}C NMR spectra of compound **4**: 2-iodobenzohydrazide

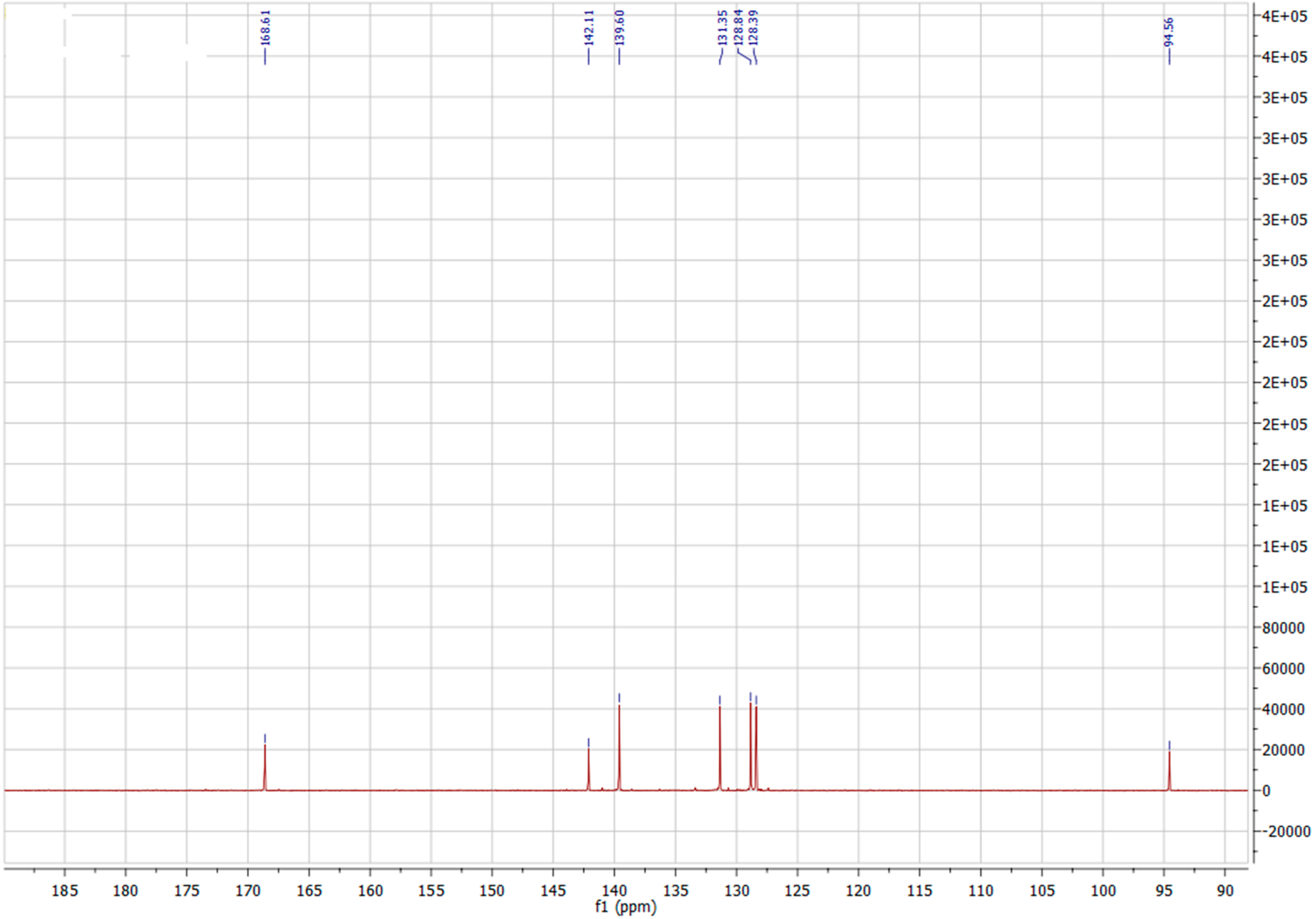

Figure S41. The IR spectra of compound **5**: 3-iodobenzohydrazide

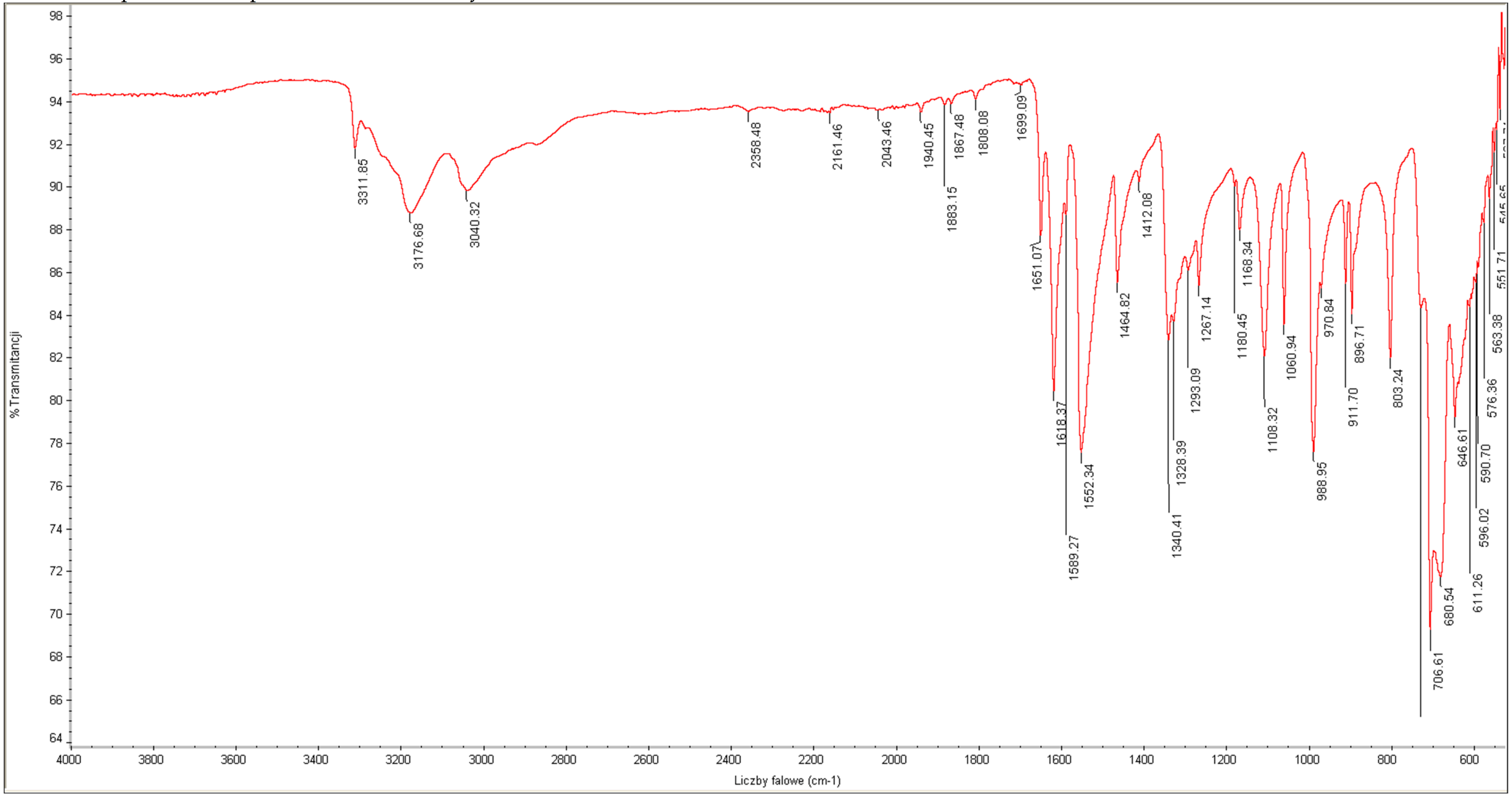

Figure S42. The ^{1}H NMR spectra of compound **5**: 3-iodobenzohydrazide

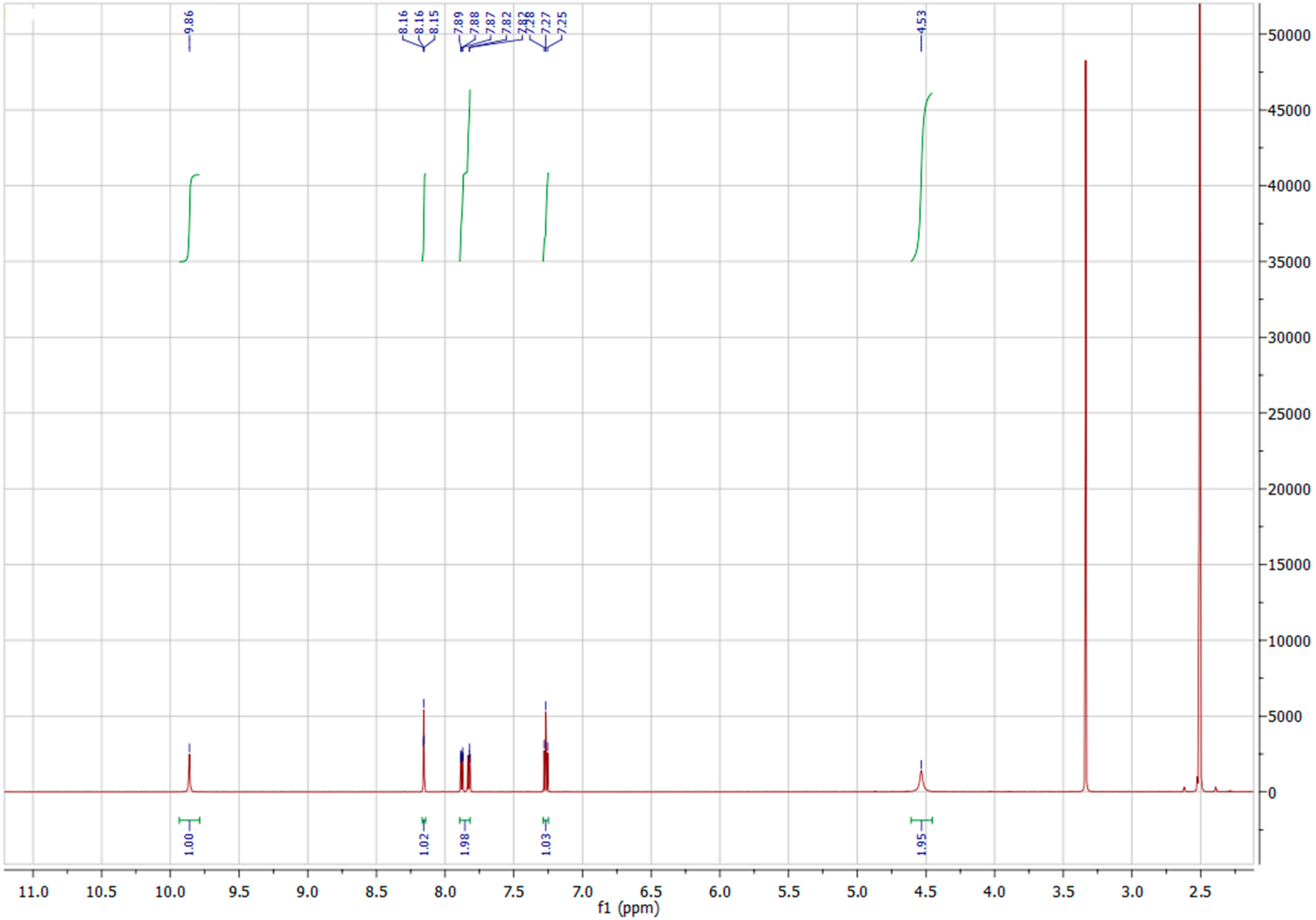

Figure S43. The ^{13}C NMR spectra of compound **5**: 3-iodobenzohydrazide

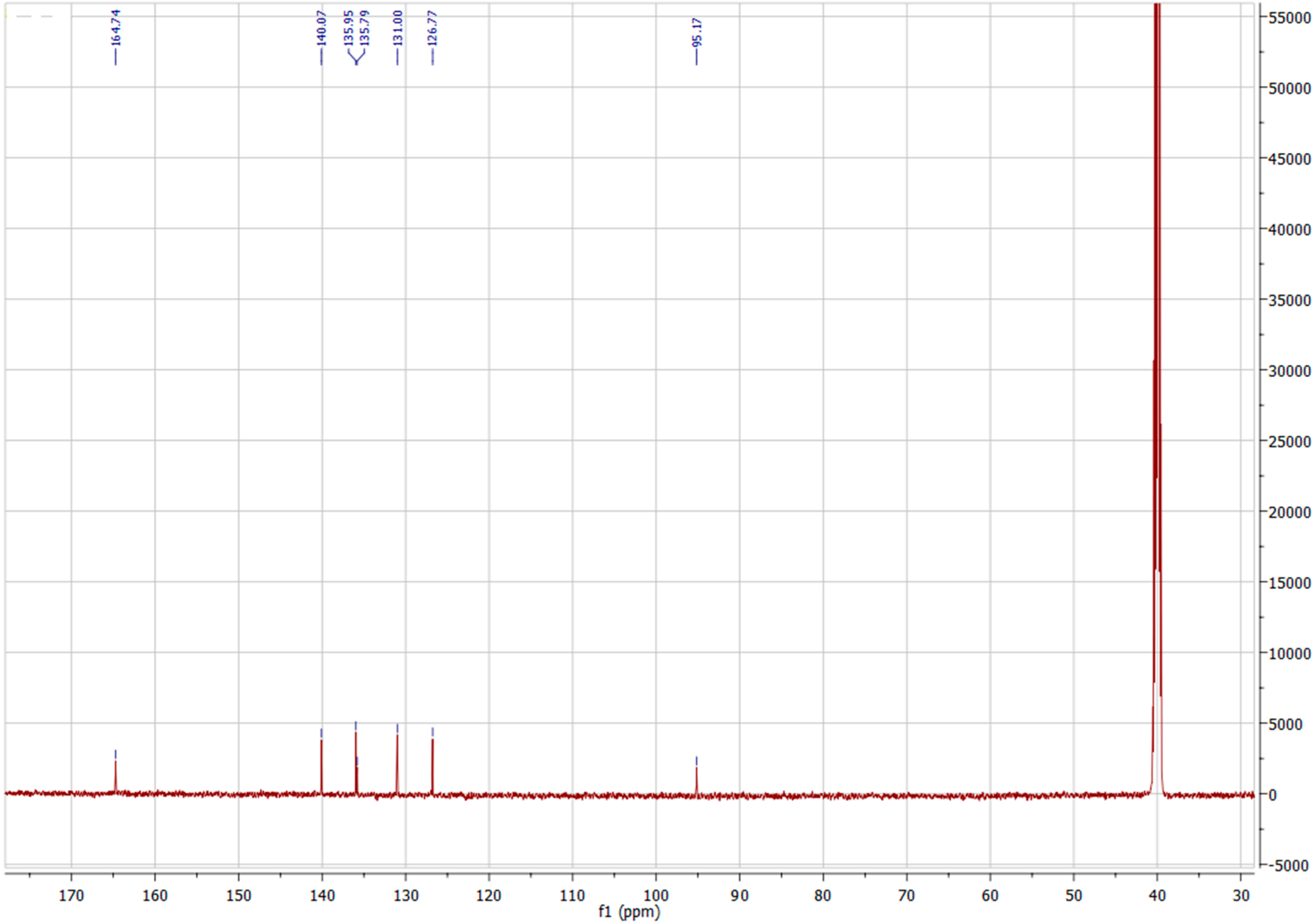

Figure S44. The IR spectra of compound **6**: 4-iodobenzohydrazide

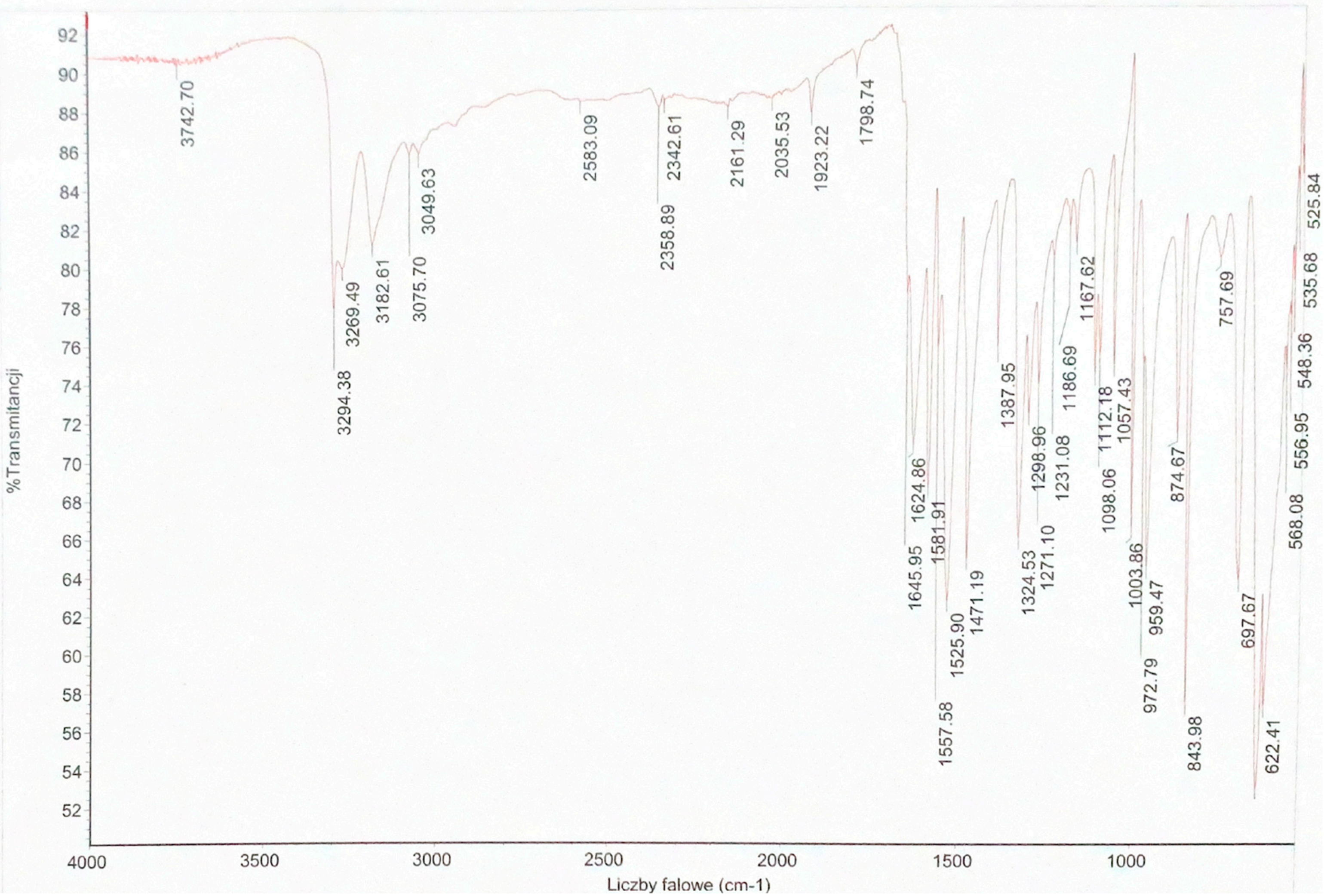

Figure S45. The ^{1}H NMR spectra of compound **6**: 4-iodobenzohydrazide

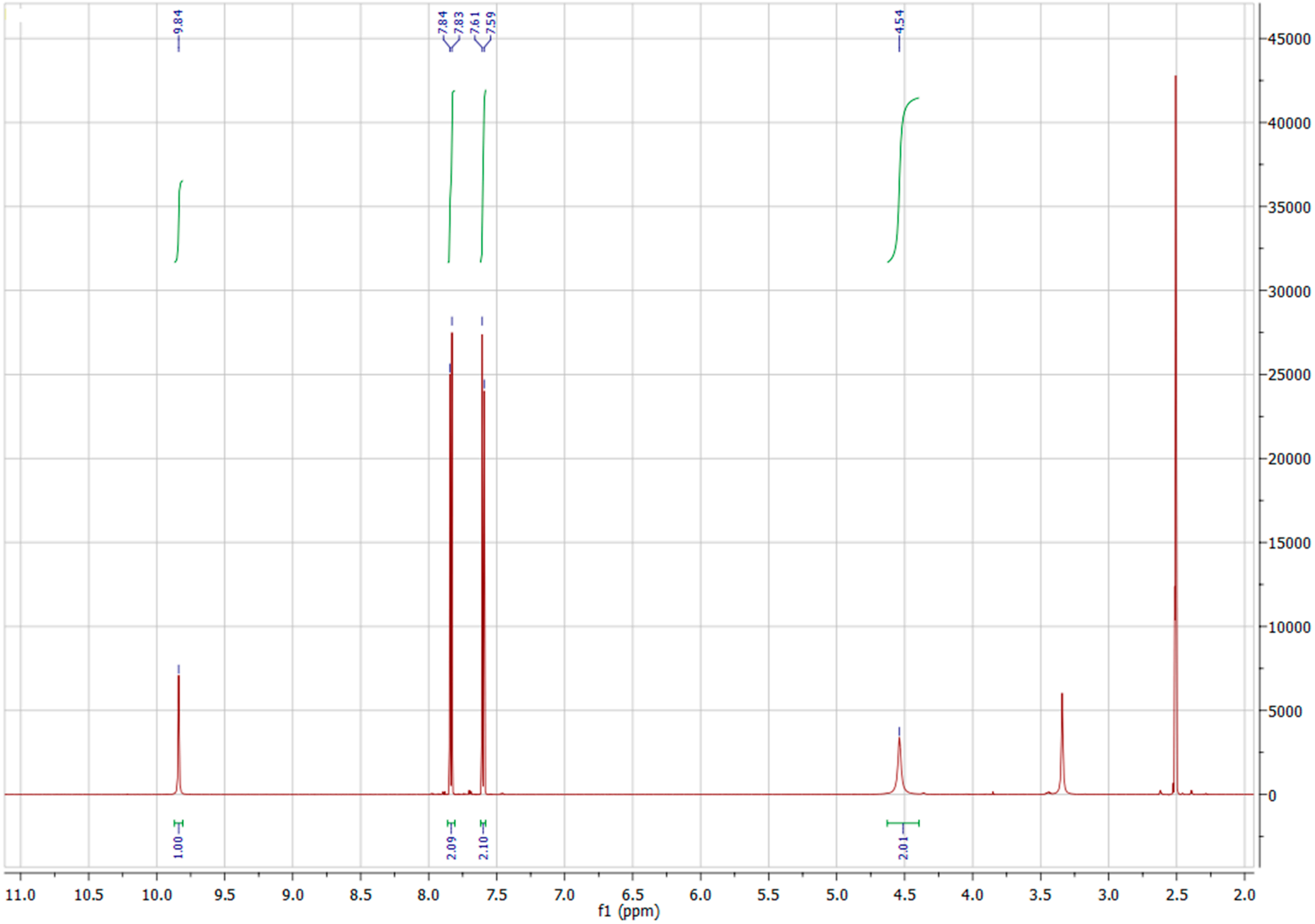

Figure S46. The ^{13}C NMR spectra of compound **6**: 4-iodobenzohydrazide

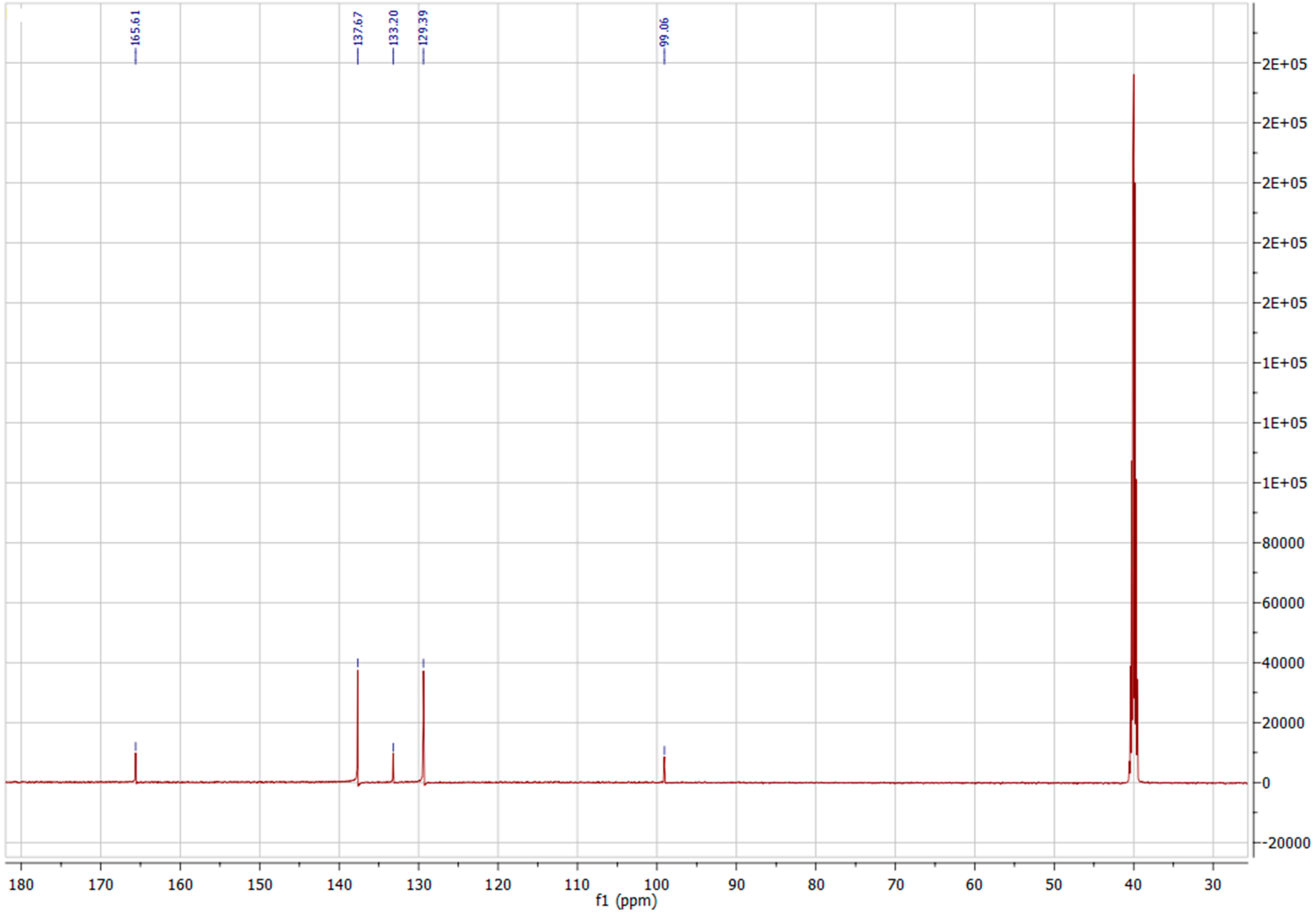

Figure S47. The IR spectra of compound **20**: *N*-[(5-chloro-2-hydroxy-3-iodophenyl)methylidene]-3-iodobenzohydrazide

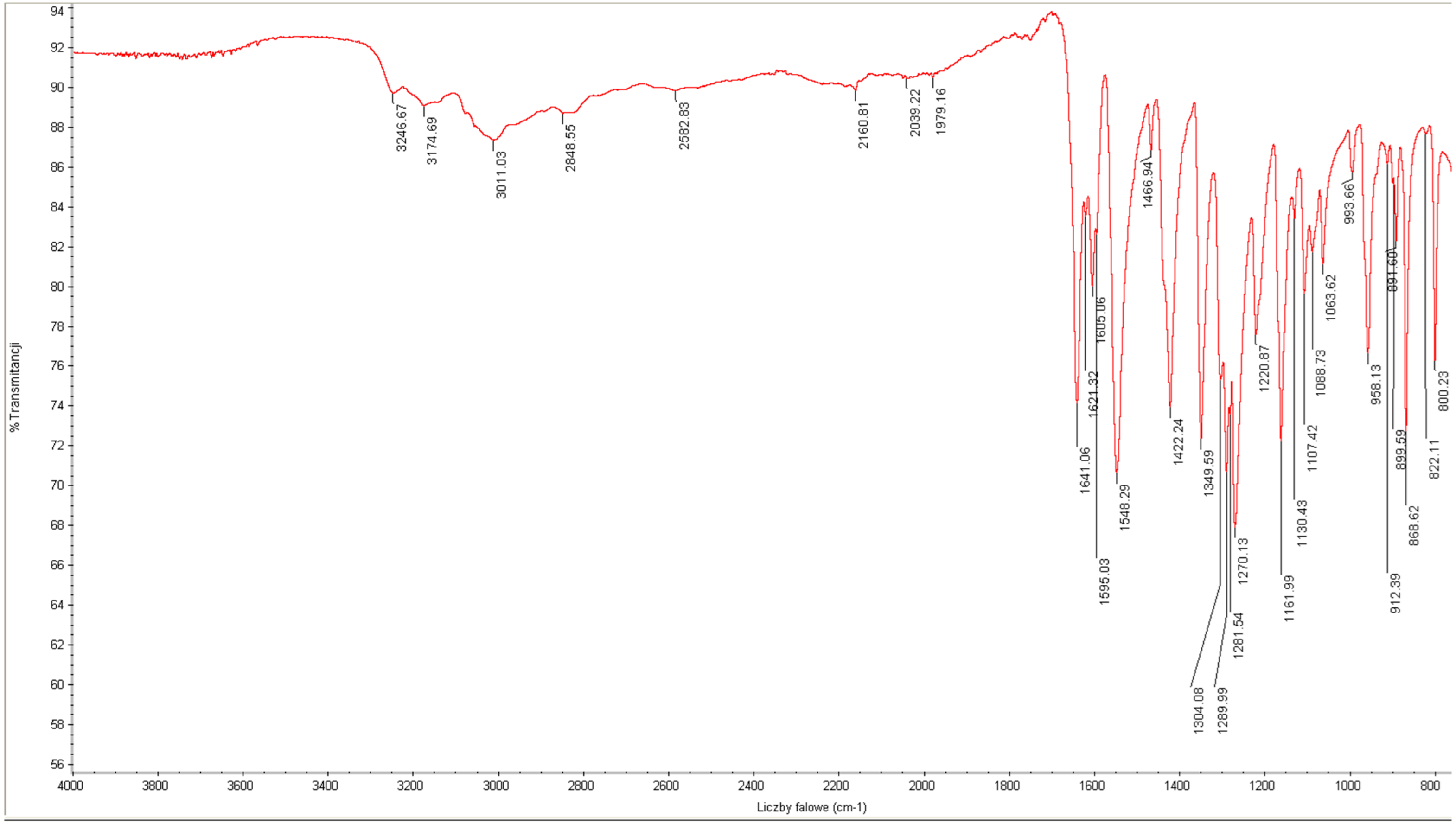

Figure S48. The ^{1}H NMR spectra of compound **20**: *N*-[(5-chloro-2-hydroxy-3-iodophenyl)methylidene]-3-iodobenzohydrazide

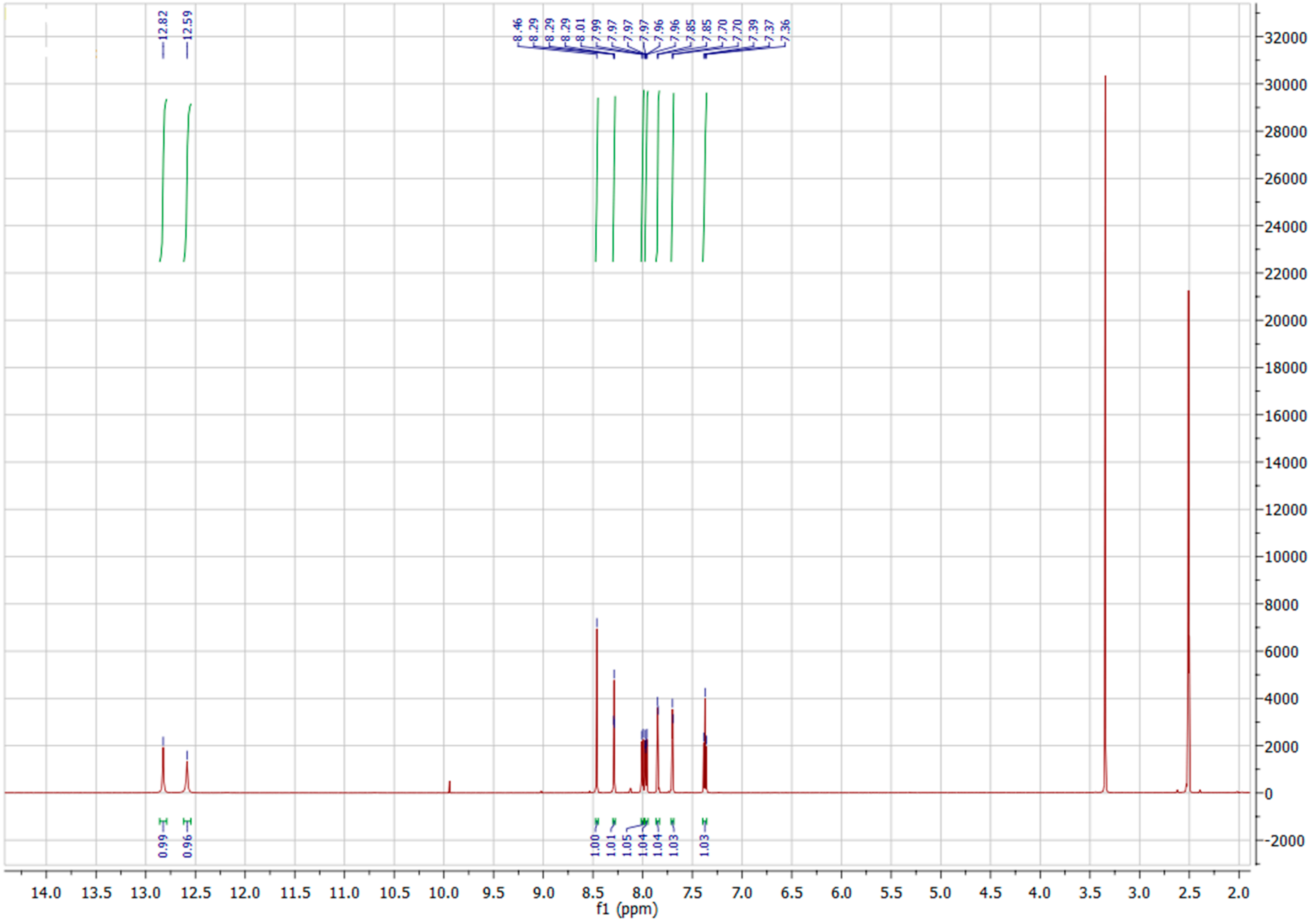

Figure S49. The ^{13}C NMR spectra of compound **20**: *N*-[(5-chloro-2-hydroxy-3-iodophenyl)methylidene]-3-iodobenzohydrazide

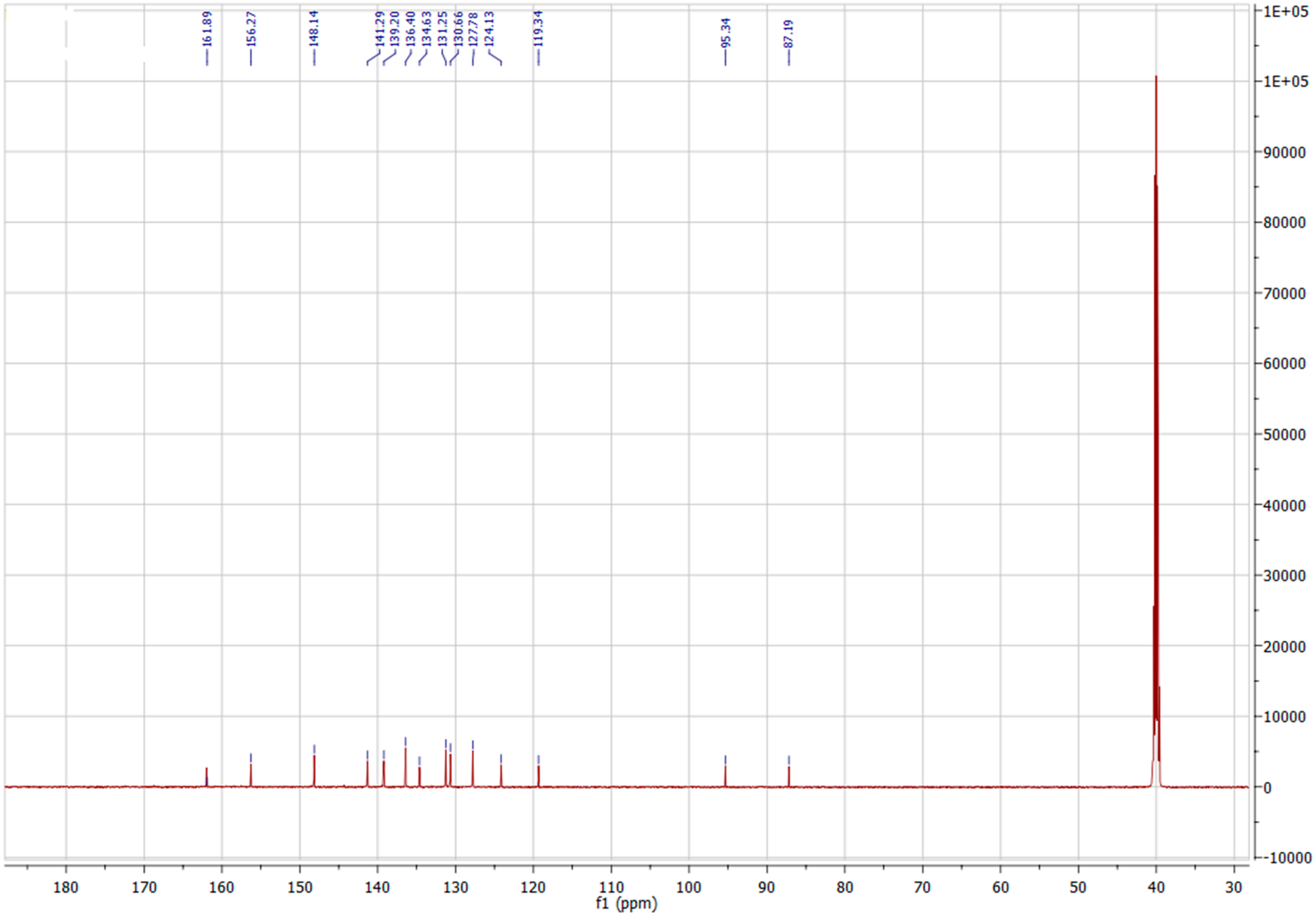

Figure S50. The IR spectra of compound **24**: *N*-[(3,5-dichloro-2-hydroxyphenyl)methylidene]-4-iodobenzohydrazide

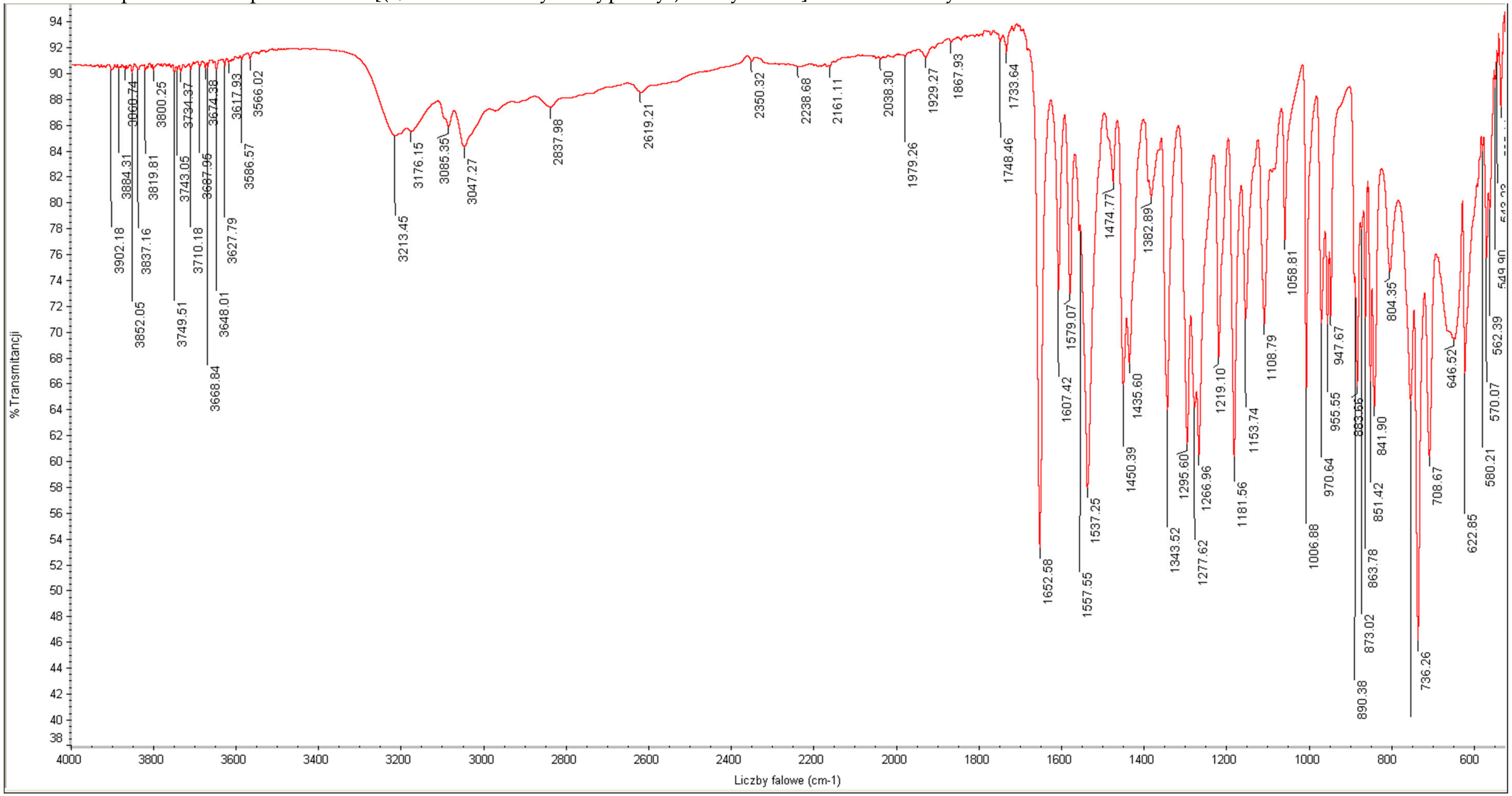

Figure S51. The ^{1}H NMR spectra of compound **24**: *N*-[(3,5-dichloro-2-hydroxyphenyl)methylidene]-4-iodobenzohydrazide

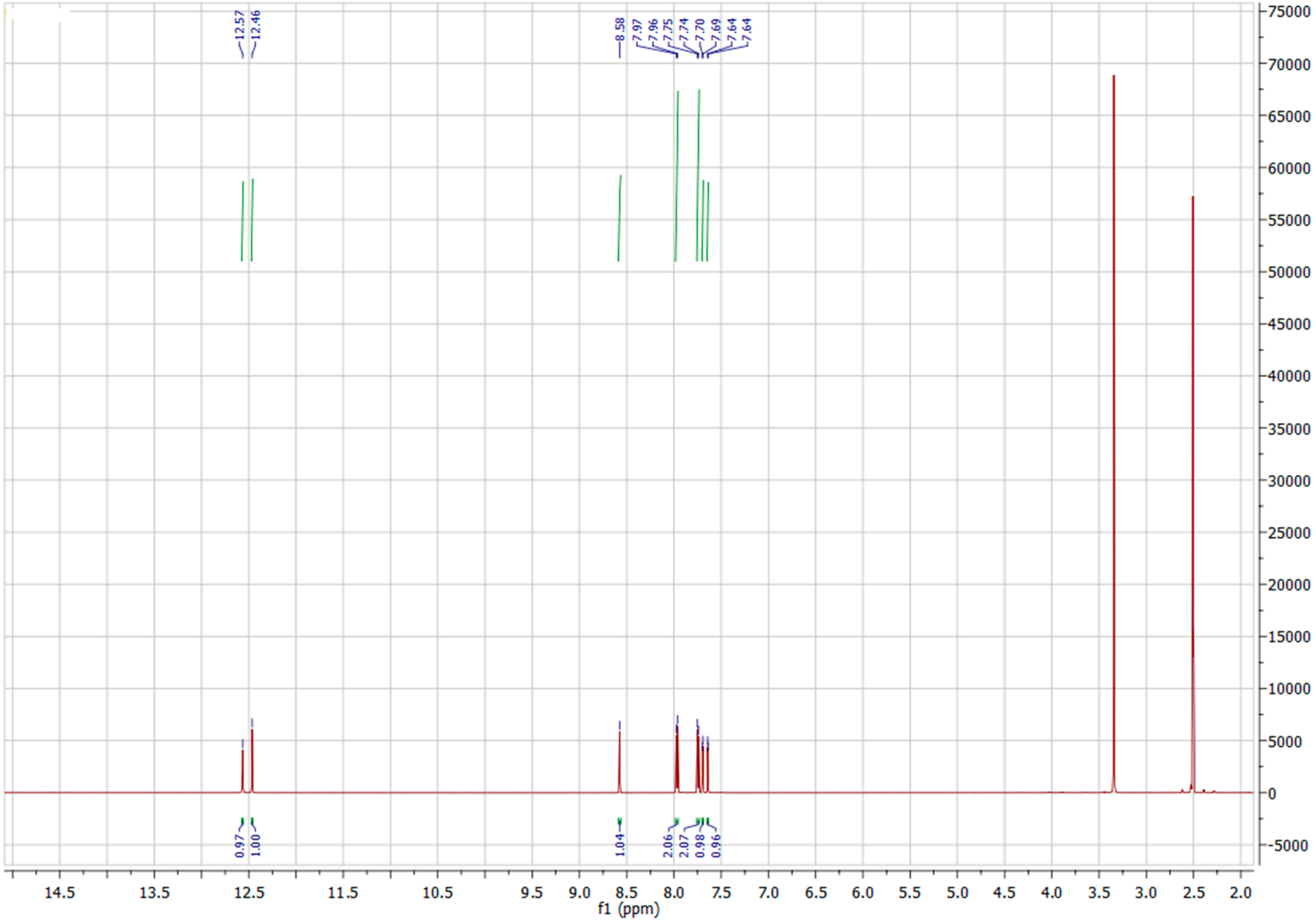

Figure S52. The ^{13}C NMR spectra of compound **24**: *N*-[(3,5-dichloro-2-hydroxyphenyl)methylidene]-4-iodobenzohydrazide

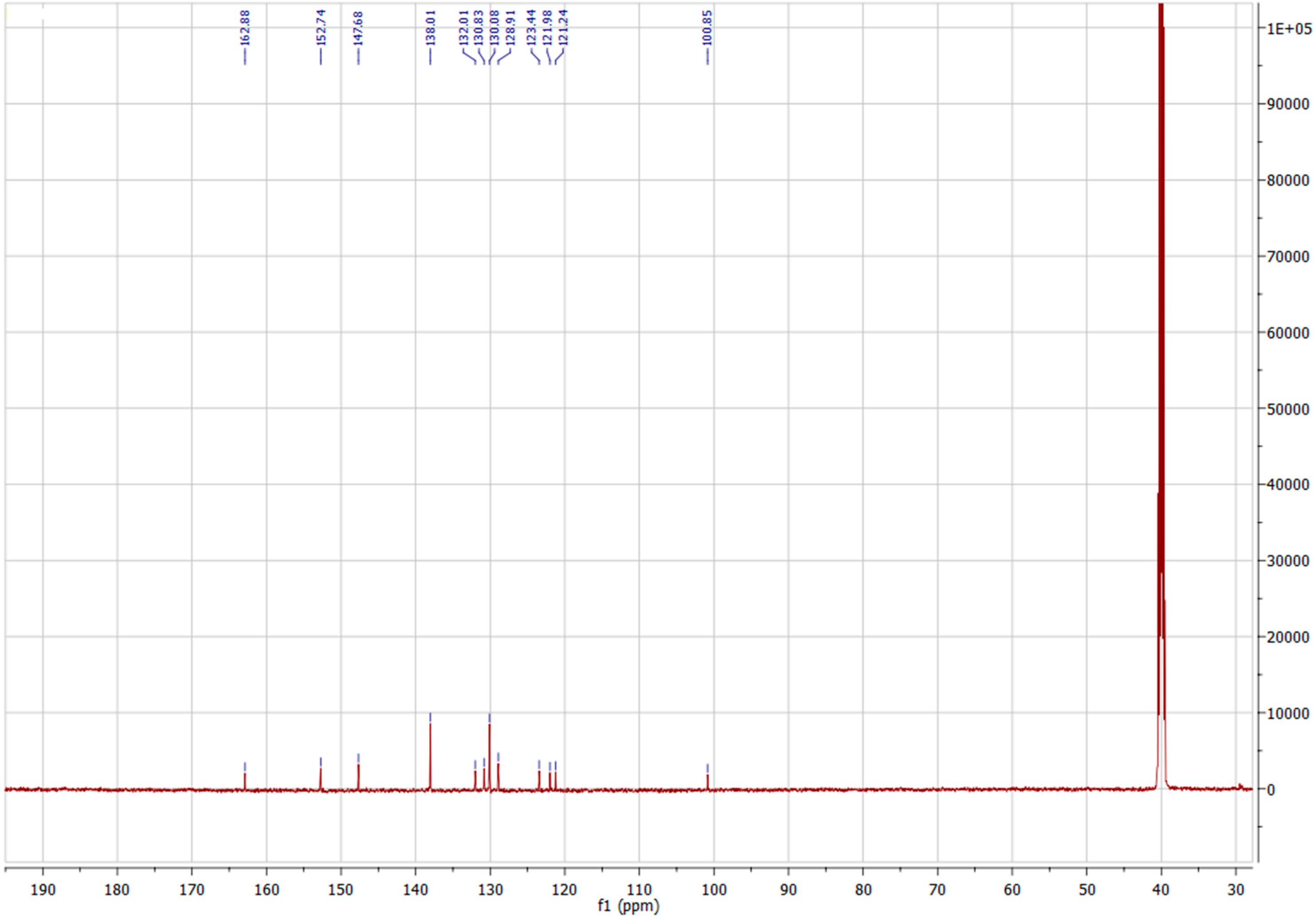

Figure S53. The IR spectra of compound **26**: *N*-[(2,5-dihydroxyphenyl)methylidene]-4-iodobenzohydrazide

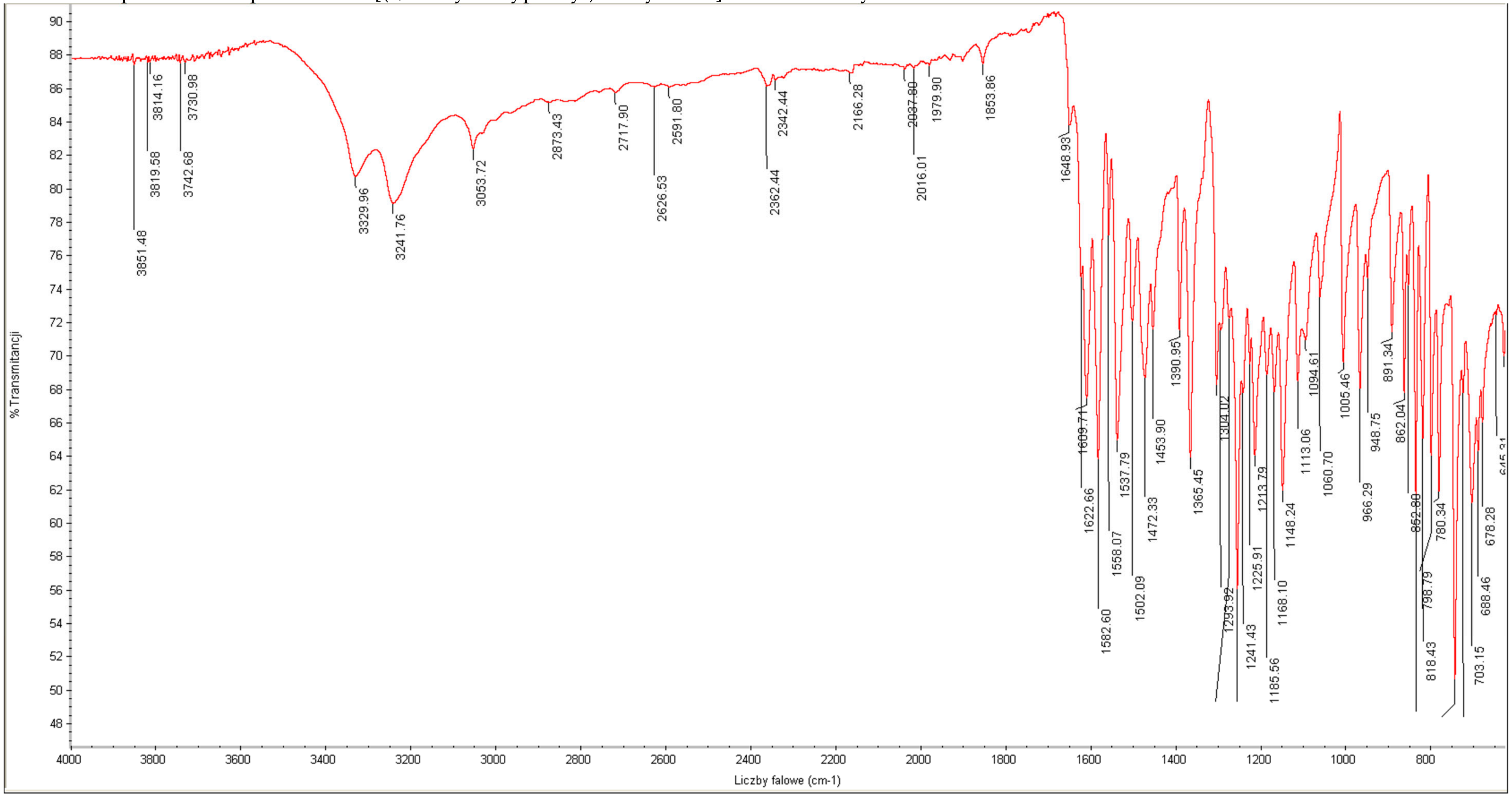

Figure S54. The ^{1}H NMR spectra of compound **26**: *N*-[(2,5-dihydroxyphenyl)methylidene]-4-iodobenzohydrazide

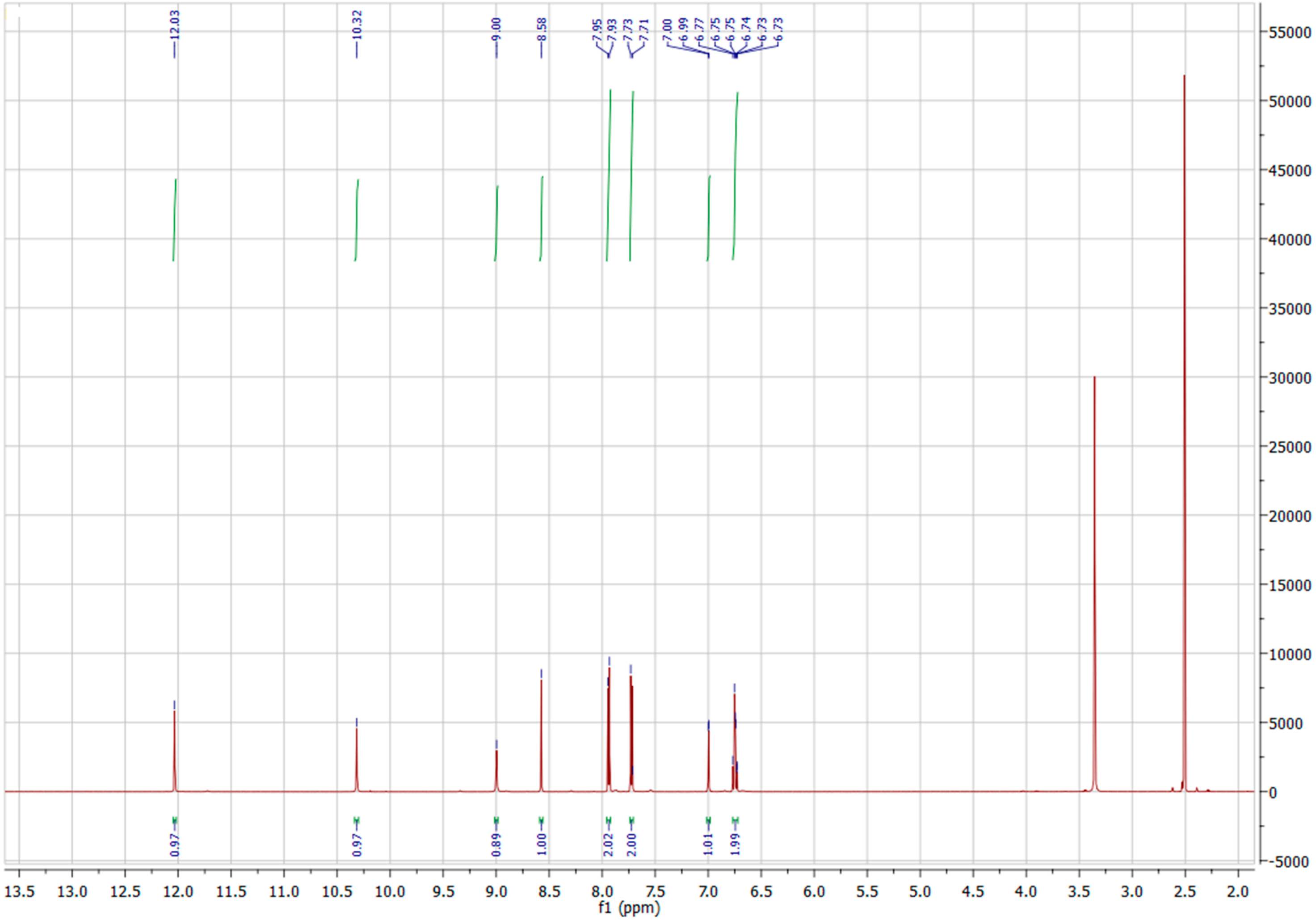

Figure S55. The ^{13}C NMR spectra of compound **26**: *N*-[(2,5-dihydroxyphenyl)methylidene]-4-iodobenzohydrazide

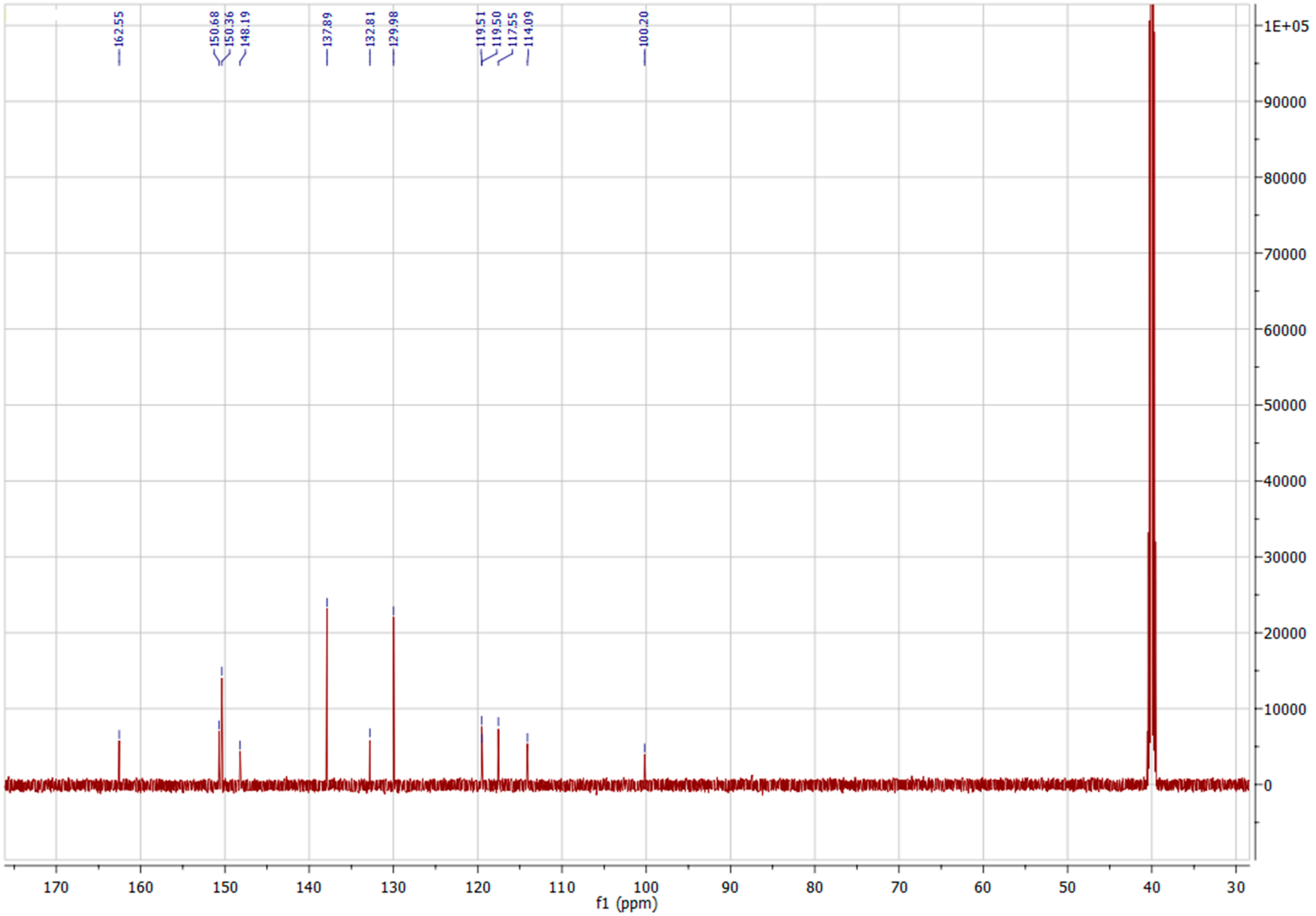

Disclaimer/Publisher's Note: The statements, opinions and data contained in all publications are solely those of the individual author(s) and contributor(s) and not of MDPI and/or the editor(s). MDPI and/or the editor(s) disclaim responsibility for any injury to people or property resulting from any ideas, methods, instructions or products referred to in the content.
